# Contagion in Mass Killings and School Shootings

**DOI:** 10.1371/journal.pone.0117259

**Published:** 2015-07-02

**Authors:** Sherry Towers, Andres Gomez-Lievano, Maryam Khan, Anuj Mubayi, Carlos Castillo-Chavez

**Affiliations:** 1 Arizona State University, Tempe, AZ, USA; 2 Northeastern Illinois University, Chicago, IL, USA; Tulane University School of Public Health and Tropical Medicine, UNITED STATES

## Abstract

**Background:**

Several past studies have found that media reports of suicides and homicides appear to subsequently increase the incidence of similar events in the community, apparently due to the coverage planting the seeds of ideation in at-risk individuals to commit similar acts.

**Methods:**

Here we explore whether or not contagion is evident in more high-profile incidents, such as school shootings and mass killings (incidents with four or more people killed). We fit a contagion model to recent data sets related to such incidents in the US, with terms that take into account the fact that a school shooting or mass murder may temporarily increase the probability of a similar event in the immediate future, by assuming an exponential decay in contagiousness after an event.

**Conclusions:**

We find significant evidence that mass killings involving firearms are incented by similar events in the immediate past. On average, this temporary increase in probability lasts 13 days, and each incident incites at least 0.30 new incidents (*p* = 0.0015). We also find significant evidence of contagion in school shootings, for which an incident is contagious for an average of 13 days, and incites an average of at least 0.22 new incidents (*p* = 0.0001). All *p*-values are assessed based on a likelihood ratio test comparing the likelihood of a contagion model to that of a null model with no contagion. On average, mass killings involving firearms occur approximately every two weeks in the US, while school shootings occur on average monthly. We find that state prevalence of firearm ownership is significantly associated with the state incidence of mass killings with firearms, school shootings, and mass shootings.

## Introduction

In recent years, tragedies involving mass killings in the US, such as the Aurora, CO movie theater shooting in July 2012, and the Newtown, CT school shooting in December 2012, have intensified societal focus on trying to understand the dynamics and contributing factors that underlie such events, particularly since the per capita incidence of such events and other firearm related mortality is significantly higher in the US than in any other industrialized country [1–4].

Statistics are not readily available on the incidence of mass killings and school shootings in other industrialized countries, however studies have shown that the firearm homicide and suicide rates in the US are several times higher than that of any other industrialized country [4], and the patterns appear to be due to higher rates of firearm ownership in the US compared with other industrialized countries [5]. Overall, the pediatric firearm mortality rate is five times higher in the United States compared with any other industrialized country [3], and 87% of all children age 0 to 14 worldwide killed by firearms are children living in the US [4], despite the fact that less than 5% of the world’s children live in the US [6]. In September, 2014, the Federal Bureau of Investigation (FBI) released a report “A Study of Active Shooter Incidents in the United States Between 2000 and 2013” [7]. In their report, they note that the incidence of mass shooting incidents has been growing over the past 14 years.

Ready access to weaponry has been implicated in these trends. For instance, it has been found that two thirds of school shooters obtained their firearms from their own home, or the home of a relative [8], and youths are significantly more likely to die by firearm homicide or suicide in states where gun ownership is more prevalent. In addition, household gun ownership has been shown to significantly raise the risk for homicide in the home [9], and homicide rates in general [10]. Studies have also shown that the more access people have to firearms, the lower the levels of social trust, and the higher the levels of homicide [11, 12]. A strong association has also been found between overall state suicide rates and firearm ownership rates [13, 14],

Access to mental health care has also been viewed as a factor in at least some types of incidents. For example, it has been shown that access to health care and the state rates of federal aid for mental health services are strongly associated with reduced state suicide rates [15].

Beyond the exongenous factors like mental health issues and ease of access to weaponry that may be contributing to the frequency of events, there is also the possibility that the stressed individuals may have, consciously or sub-consciously, been inspired to act on previously suppressed urges by exposure to details of similar events. Such contagious ideation is not implausible; for example, vulnerable youth have been found to be susceptible to suicide ideation brought on by influence of reports and portrayal of suicide in mass media [16–18], and media reports on suicides and homicides have been found to apparently subsequently increase the incidence of similar incidents in the community [19, 20]. In addtion, in a broader public health context, the temporal and geospatial clustering of many types of violence and crime have been shown to be similar to the patterns seen in the spread of infectious disease [21].

To study this issue, we examine data on mass killings (four or more people murdered, by any means) in the US between 2006 to 2013 compiled by USA Today (*N* = 232, 176 of which involved firearms), data on school shootings from 1998 to 2013 inclusive from the Brady Campaign to Prevent Gun Violence (*N* = 188), and data on mass shootings (at least three people shot, not necessarily killed) from February 2005 to January 2013, also from the Brady Campaign (*N* = 477).

We fit a mathematical contagion model to the data sets, with model terms that take into account temporal trends due to possible exogenous non-contagion factors, and a contagion term that takes into account the fact that a school shooting or mass murder may temporarily increase the probability of a similar event in the immediate future. We model the contagion process assuming an exponential decay in contagiousness after an event. Contagion models have been applied to financial markets [22], spread of YouTube videos on social networks [23], burglary [24–27], civilian deaths in Iraq [28], and terrorist attacks [29, 30], but this is the first instance in which these models have been applied in the context of mass murders and school shootings.

Because contagion is just one potential aspect contributing to these events, we also examine the relationship between the incidence of these tragedies, and state prevalence of mental illness and firearm ownership, and the rankings of the strength of state firearm legislation.

In the following sections we describe the sources of data used in this analysis, and the modeling and statistical data analysis methods used. We then present results, with discussion and summary.

## Methods

### Data

Here we describe the mass killing and school shooting sources of data used in this analysis, along with a description of the data sources use to examine the relationship of such events to state prevalence of mental illness and firearm ownership, and estimates of the strength of state gun laws. There are currently no comprehensive federal repositories of data on mass killings and school shootings in the US, thus for these studies we relied on data compiled by private organizations. Data are available at http://tinyurl.com/oql4lpy.

#### USA Today mass killing data

Data on mass killings in the US between 2006 to December 2013 were obtained from a USA Today study that examined Federal Bureau of Investigation (FBI) data from the FBI Supplemental Homicide Reports, and hundreds of media reports and police documents to compile a list of incidents that involved four or more people being killed, not including the killer (data are available from, and described at, masskillings.usatoday.com, accessed March 2014). The USA Today study did not rely solely upon the FBI data, in part because the FBI data are based on voluntary reports by local police agencies and are thus an incomplete tally of mass murders in the US, and also because the data were found to only have a 61% accuracy rate when compared with data available from local police documents, apparently largely due to mistakes in transcription. For more details about the USA Today data set, see www.usatoday.com/story/news/nation/2013/12/03/fbi-mass-killing-data-inaccurate/3666953/ (accessed January 30, 2015).

**Fig 1:**
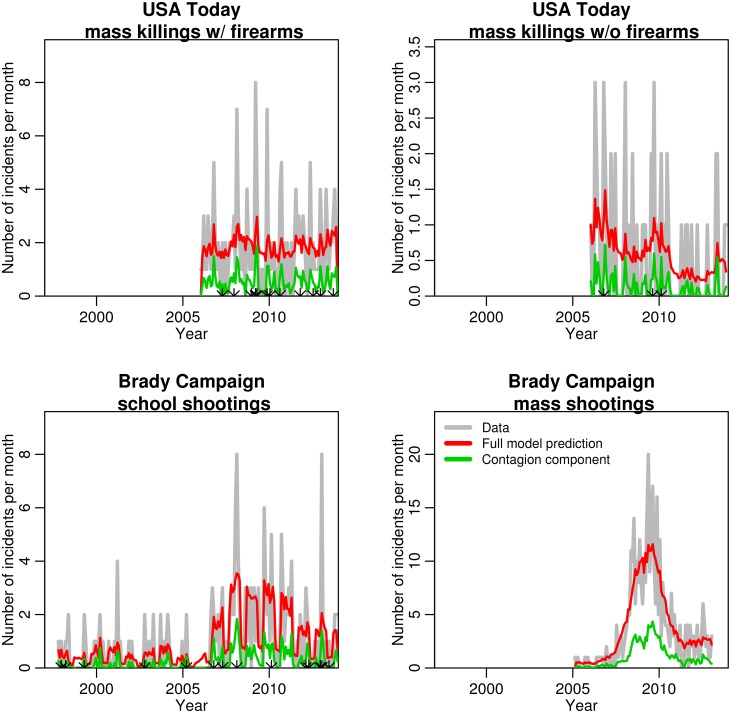
Number of mass killings, school shootings, and mass shootings over time for the data samples used in these studies. For clarity of presentation, all data samples are shown over the same time frame. Overlaid is the fit of the full model of Eq 4 that also contains a contagion component (red). The green line indicates the estimated portion of the data due to contagion. The points along the *x* axis for the first three samples indicate the date of events that had number of people killed in the top 5^th^ percentile for that sample.

The data consist of 232 events, of which firearms were involved in 176 (76%). Of these 232 incidents, the authors found only three apparent errors, where the date was accidentally transcribed incorrectly by one day. The dates were corrected for this analysis. The data are shown in Fig 1.

Incidents showed no significant dependence on month of the year, but were significantly more likely to occur on Saturdays (Pearson *χ* ^2^ *p* = 0.04). For the 176 incidents that involved mass killings with firearms, 46% of the killers committed suicide (either by themselves or by “suicide by cop”). This is significantly higher than the estimated 5% to 10% suicide rate of killers in all murders [31]. On average, 22% more people were killed (not including the killer) in events where the murderer committed suicide (Student-t two-sided *p* = 0.038).

This is in contrast to the 56 events involving mass killings without firearms, where only 8% of such killers committed suicide, and there was no significant difference in the number killed when the murderer committed suicide (Student-t two-sided *p* = 0.74).

#### Brady Campaign School Shooting Data

Data on school shootings in the US were obtained from the Brady Campaign to Prevent Gun Violence, who examined media reports to compile a list of 220 school shootings in the US between 1997 to 2013, inclusive. We excluded from this data set foiled plots where individuals were caught actively planning a shooting, but had not yet carried out their plan, incidents that did not actually occur on a school campus or school bus, or incidents that occurred outside of school hours or outside the context of school-related events (such as sports games), leaving 188 events. Of these 188 events, upon review of the media reports the authors found four incidents for which the date was apparently mis-transcribed by one day, and the dates were corrected for this analysis. The school shootings resulted in an average of one person being killed per incident; only six of the school shootings thus overlap with the USA Today mass killing data set, and the two data sets are nearly independent. The data are shown in Fig 1.

The incidents were significantly more likely to occur between September to April compared with May to August (*p* < 0.001), and on weekdays compared with weekends (*p* < 0.001).

Of all events, 18% of the shooters committed suicide, with the fraction rising to 23% in incidents in which at least one person was killed (not including the shooter). Again, this is significantly higher than the estimated 5% to 10% suicide rate of killers in all homicides [31]. There was no significant difference in the number of people killed in incidents where the shooter did, and did not, commit suicide.

#### Brady Campaign Mass Shooting Data

Additional data on 477 mass shootings in the US between February 2005 to January 2013 were also obtained from the Brady Campaign. These data include shootings where at least three people were shot or injured (but not necessarily killed).

Incidents were excluded that did not mention a verifiable source, or were an agglomeration of events rather than a single event (such as a mention of the total number of people killed by firearms in particular city over a weekend), and we were unable to identify news stories containing the details of the separate events. In addition, two of the entries appeared to be repetitions of a previous entry and were thus excluded from the analysis. A total of 468 incidents remained after these selections. Ten of the events had the date of the incident mis-transcribed by a day or two, and the dates were corrected for this analysis. Of the 468 mass shootings, 92 involve 4 four or more people killed, 83 of which are also included in the 154 mass killings by firearms in the USA Today data set that spans the same time frame. Because of this large overlap of the two data sets, in order to ensure that the Brady Campaign mass shooting data is independent of the USA Today mass killing data we thus examine only the mass shootings in the Brady Campaign data that have less than four people killed, yielding a total of 376 incidents. The data are shown in Fig 1.

Incidents showed no significant dependence on month of the year, but were significantly more likely to occur on Saturday and Sunday (*p* < 0.001).

Of all events, 17% of the shooters committed suicide, with the fraction rising to 25% in incidents in which at least one person was killed (not including the shooter). Again, this is significantly higher than the estimated 5% to 10% suicide rate of killers in all homicides [31]. On average, 2.3 times more people were killed in events where the shooter committed suicide, compared with events where the shooter did not (Student-t *p* < 0.001).

#### Correlation of data samples to exogenous variables

No official government statistics are available for firearm ownership by state. In order to estimate the relative firearm ownership by state, we thus examined the fraction of all suicides within each state that involved firearms (past firearm suicide rates have been shown to be directly correlated to firearm ownership rates [13, 14], and are a good proxy for firearm ownership rates [32]). Suicide data from 1999 to 2010 by state and cause used to calculate these estimates were obtained from the Centers for Disease Control Web-based Injury Statistics Query and Reporting System (http://www.cdc.gov/injury/wisqars/index.html, accessed January 30, 2015). State populations in 2012 were obtained from the US Census Bureau www.census.gov (accessed January 30, 2015).

State prevalence rates of serious mental illness for all 50 states were obtained from the from the National Alliance on Mental Illness (NAMI) [33, 34].

The Brady Campaign to Prevent Gun Violence regularly publishes rankings of the states on the basis of laws that can prevent gun violence, such as background checks on all gun sales, permit-to-purchase requirements, limiting handgun purchases to one a month, and retention of sales records. The most recent state rankings were obtained from bradycampaign.org.

To study how the state variations in incidence of mass killings, school shootings, and mass shootings may be related to exogenous variables such as state rates of firearm ownership, prevalence of mental illness, etc, we first calculated the national average rate of such incidents, *μ*. For a state with observed number of incidents, *k*_*i*_, and population *N*_*i*_, we determined the significance of the observed number of incidents by calculating the Binomial CDF

$$\begin{array}{c} F\left(k_{i}|N_{i},\mu \right)=\sum_{j=0}^{k_{i}}\left(\begin{array}{c} N_{i}\\ j \end{array}\right)\mu^{j}{\left(1-\mu \right)}^{N_{i}-j}. \end{array}$$ (1)

The result is between 0 and 1, with results close to 0 (1) indicating that a particular state has rate of such incidents significantly lower (higher) than the national average.

### Modeling and Statistical Methods

#### Self-Excitation Contagion Model

In a self-excitation contagion model, recent prior events increase the probability of another event happening in the near future. In this analysis, we employ an exponential probability distribution to simulate this process. Under the hypothesis that past events incite future events, the increased probability of an event occurring during the 24 hours of day *t*_*j*_ due to prior event that occurred on day *t*_*i*_ is thus

$$\begin{array}{c} P\left(t_{j}|t_{i},T_{\text{excite}}\right)=\int_{t_{j}-t_{i}-1}^{t_{j}-t_{i}}dx\;\frac{e^{-x/T_{\text{excite}}}}{T_{\text{excite}}}, \end{array}$$ (2)

where *T*_excite_ is the average duration of the contagion process (in this analysis, measured in days). This yields

$$\begin{array}{c} P\left(t_{j}|t_{i},T_{\text{excite}}\right)=e^{-\left(t_{j}-t_{i}-1\right)/T_{\text{excite}}}-e^{-\left(t_{j}-t_{i}\right)/T_{\text{excite}}}. \end{array}$$ (3)

We consider a self-excitation contagion model with an additional baseline (i.e.; non-contagion related) average number of events per day of *N*_0_(*t*). Taking into account all prior events in some stochastic data realization, the total number of expected events, *N* ^exp^, on day *t*_*n*_ for that realization is thus

$$\begin{array}{c} N^{\text{exp}}\left(t_{n}\right)=N_{0}\left(t_{n}\right)+N_{\text{secondary}}\sum_{\forall t_{i}<t_{n}}P\left(t_{n}|t_{i},T_{\text{excite}}\right), \end{array}$$ (4)

where the summation is over all prior events. The parameters of this contagion model are the average number of secondary events inspired by the contagion of a single event, *N*_secondary_, the duration of the contagion process, *T*_excite_, and whatever parameters are needed to describe the temporal evolution of the baseline number of events, *N*_0_(*t*). For instance, one can assume *N*_0_(*t*) is a constant, or a straight line with a slope. One can also use a non-parametric approach where *N*_0_(*t*) is calculated using a weighted running mean of the data itself (in S1 Appendix we provide a detailed description of the weighted running mean ansatz).

The functional form of *N*_0_(*t*) can incorporate additional weights if needed in order to take into account day-of-week or seasonal effects, and indeed, we incorporate such weights when it is evident that there is significant evidence of dependence on season or day-of-week in a particular data set.

In S1 Appendix, we describe the methods used to fit the parameters of the model of Eq 4 to data, and the methods we used to simulate data with a self-excitation contagion model. We also describe the methods used to validate the modeling and fitting methods, and the methods used to cross-check the analysis.

## Results

Based on the USA Today sample, mass killings involving firearms with four or more people killed occur on average every 12.5 days in the US. Based on the Brady Campaign data, school shootings occur on average every 31.6 days.

The results of the fits of the parameters of the self-excitation contagion model of Eq 4 to the various data samples are shown in Fig 1, and are tabulated in Table 1. Evidence of significant contagion is found for all samples but the Brady Campaign mass shooting sample with at most three people killed.

**Table 1:** Results of fits of the self-excitation contagion model in Eq 4 to the various data samples considered in these studies, using a running mean calculation of *N*_0_(*t*). *N*_secondary_ is the average number of new incidents. The p-value is obtained from the likelihood ratio test comparing the likelihood of the full contagion model to the likelihood of the null hypothesis model of no contagion. The numbers in the square brackets indicate the 95% confidence interval on the parameter.

Sample	# in sample	*N*_secondary_	*T*_excite_	*p*−value
USA Today full sample	232	0.28 [0.10,0.56]	13.3 [4.6,58.6]	*p* = 0.004
USA Today w/ firearms	176	0.30 [0.12,0.56]	13.2 [4.3,46.8]	*p* = 0.002
USA Today w/o firearms	56	0.23 [0.04,0.64]	11.9 [3.2,89.5]	*p* = 0.037
Brady Campaign school shootings	188	0.22 [0.10,0.42]	12.9 [5.4,53.3]	*p* < 0.001
Brady Campaign mass shootings w/ ≤ 3 killed	373	0.28 [0.00,0.62]	38 [0, 90]	*p* = 0.18

Latitude and longitude data were obtained for the location of each incident in the data sets used in this analysis. For all samples, the time between incidents was not significantly correlated to the distance between them, indicating lack of evidence of temporal/geospatial clustering (as would happen, for instance, if an incident incited a similar incident in a nearby locale). Additionally, the Mantel test for temporal/geo-spatial clustering in the samples did not return significant *p*-values (*p* > 0.05 for all samples) [35]. This lack of temporal/geo-spatial correlation is consistent with what would be expected if the contagion process is potentially due, for instance, to widespread media attention given to mass killings and school shootings.

**Fig 2:**
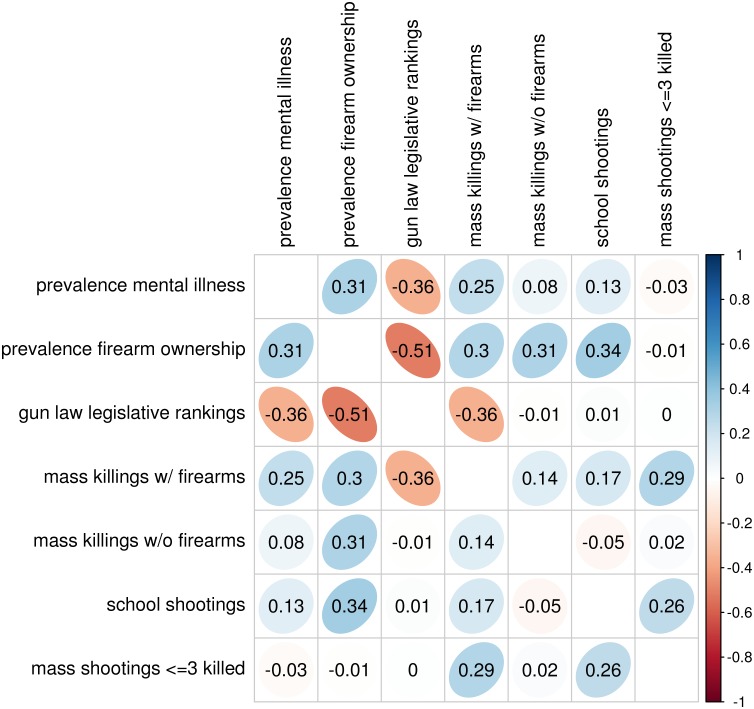
Relationship of state prevalence of firearm ownership, mental illness, and state rankings of strength of firearm legislation, to the state incidence of mass killings, school shootings, and mass shootings. Correlations with ∣*ρ*∣ ≥ 0.28 are significant to *p* < 0.05.

The correlation of the state incidence of our data samples to firearm ownership prevalence, prevalence of mental illness, and ranking of strength of state firearm legislation are summarized in Fig 2.

## Discussion and Summary

In our analysis, we employ a self-excitation contagion model, and find significant evidence of contagion in mass killings and school shootings. There is no significant evidence of contagion in mass shootings that involve three or fewer people killed, possibly indicating that the much higher frequency of such events compared with mass killings and school shootings reduces their relative sensationalism, and thus reduces their contagiousness. This is the first analysis of its kind to examine the potential of contagion in such incidents.

We find that state prevalence of firearm ownership is significantly associated with state incidence of mass killings with firearms, school shootings, and mass shootings. Once state prevalence of firearm ownership has been taken into account, there is no significant association between state incidence of these events and state prevalence of mental illness or ranking of strength of firearm legislation. Mass killings not involving firearms are not significantly correlated to any of these variables. Our noted apparent relationship of high-profile shooting incidents to firearm ownership is in concordance with the results of other studies of firearm violence, including homicide and suicide [8–14, 36].

Given the significant correlation of the incidence of mass killings to firearm ownership prevalence, it is worthwhile to explore the potential reasons for the apparent relationship. We note that the overall effect of firearm right-to-carry laws on the prevalence of various types of crime has been hotly debated (see, for instance, References [37–41]). However, it has been shown that firearm regulations that reduce overall firearm availability, such as permit and licensing regulations, appear to have a significant deterrent effect on suicide rates [42], and shall-issue laws that eliminate most restrictions on carrying a concealed weapon have been associated with increased firearm homicide rates [43]. In addition, firearm mortality rates were found to be significantly lower in states with stricter gun laws [44]. Secure gun storage practices involving safes and trigger locks have been shown to decrease the risk of youth suicides and unintentional firearm injuries [45].

We found no significant association between the rate of school and mass shootings and state prevalence of mental illness. However, we note that in all our data samples but mass killings not involving firearms, the probability that the perpetrator committed suicide was several times higher than the overall estimated perpetrator suicide rate of 5% to 10% for all homicides [31]. In addition, in our mass killings with firearms and mass shootings data samples, we have found a significant positive association between the overall number killed and perpetrator suicide. Further study of the reasons behind these patterns is indicated, including an examination of whether or not problems with access to mental health treatment at the individual level played a partial role in such incidents.

While our analysis was initially inspired by the hypothesis that mass media attention given to sensational violent events may promote ideation in vulnerable individuals, in practice what our analysis tests is whether or not temporal patterns in the data indicate evidence for contagion, by whatever means. In truth, and especially because so many perpetrators of these acts commit suicide, we likely may never know on a case-by-case basis who was inspired by similar prior acts, particularly since the ideation may have been subconscious.

It is also unclear whether or not the patterns we have observed in our analysis are perhaps spuriously caused by potential unaccounted-for biases in the data samples that were compiled by private organizations. To further the understanding of the underlying root causes of these events, and to confirm whether or not contagion truly plays a role, an official comprehensive detailed, accurate, and publicly available federal database of incidents of all mass killings and school shootings in the US is necessary. A database that includes, at a minimum, details on the background events, mental health status and access to mental health treatment of the perpetrators, exactly what kinds of weapons were used, where the perpetrators obtained their weapons, and whether they did so legally or illegally. Several studies of firearm violence over the past decade have pointed out the need for such a database (see, for instance, References [46, 47]). For the time being, while waiting for such a database to become available, studies such as this must use what data are available, paying attention to cross-checks of the robustness of the modeling methodology to potential biases, as we have attempted to do here.

Studies into the prevention of such tragedies are also hampered by the freeze on federal funding for research into gun violence in the United States, put in place by Congress in 1997 [48, 49]. In January, 2013, President Obama issued a presidential memorandum directing the Centers for Disease Control to resume studies into firearm violence. However, at the time of this writing in September 2014, the majority of members of Congress have vowed to continue to block allocation of federal funding to the studies. In the near term it thus appears that federal legislation will not be put forward to address the need for the documentation and detailed study of such events.

## Supporting Information

S1 Appendix (PDF)

S1 Data A compressed directory containing the data files used in this study. (GZ)
