# Supplementary material for: Contagion in Mass Killings and School Shootings
Source: PLoS One. 2015 Jul 2;10(7):e0117259. doi: 10.1371/journal.pone.0117259 (PMC4489652; doi:10.1371/journal.pone.0117259)
Supplement: S1 Appendix — (PDF) [file pone.0117259.s001.pdf]

Appendix S1

Modeling methods, estimation of model parameters, analysis cross checks, and model validation

Maximum likelihood estimation of self-excitation contagion model parameters

Given a set of data events, X_i, observed at times s_i with $i = 1, ..., Q$, we create a new vector, $\vec{t}$, of sequential days from time s_1 to s_Q (ie; this vector is of length $M = s_Q - s_1 + 1$). We also create a new data vector, $\vec{Y}$, also of length M, where the vector is filled at the appropriate dates with the data in X, and is zero otherwise.

We consider a self-excitation contagion model with an additional baseline (i.e.; non-contagion related) average number of events per day of $N_0(t)$. Taking into account all prior events in some stochastic data realization, the total number of expected events, $N^{\rm exp}$, on day t_n for that realization is thus

$$N^{\rm exp}(t_n) = N_0(t_n) + N_{\rm secondary} \sum_{\forall t_i < t_n} P(t_n | t_i, T_{\rm excite}), \qquad (1)$$

where the summation is over all prior events. The parameters of this contagion model are the average number of secondary events inspired by the contagion of a single event, $N_{\rm secondary}$, the duration of the contagion process, $T_{\rm excite}$, and whatever parameters are needed to describe the temporal evolution of the baseline number of events, $N_0(t)$.

Let us refer to the parameters of the self-excitation contagion model for $N^{\rm exp}(t)$ in Equation 1 as vector $\vec{\theta}$ (these parameters include $N_{\rm secondary}$, $T_{\rm excite}$, and whatever parameters are needed to parameterize the temporal evolution of the baseline average number of events per day, N_0). We find the best-fit model to the data by finding the value of $\vec{\theta}$ that minimizes the negative Poisson log-likelihood

$$-\log \mathcal{L}(\vec{Y}|\vec{\theta}) = \sum_{i=i_0}^{M} N^{\rm exp}(t_i|\vec{\theta}) - Y_i \log N^{\rm exp}(t_i|\vec{\theta}), \qquad (2)$$

where the fit is performed to observations i_0 to M; to take into account the fact that the data are left censored, meaning that information about possible earlier events in the self-excitation process are missing, and to avoid potential bias this censoring might cause in the estimation of the parameters of the self-excitation process, we choose i_0 to be large enough that the calculation of $N^{\rm exp}$ includes several

prior events. We nominally take $i_0 = 365$ days, and cross-check each analysis with i_0 equal to two years to ensure that the results do not significantly change.

We simulated data samples of the same sizes and temporal variation (including weekday and seasonal variation) of the samples used in this study to affirm that the fitting ansatz produced unbiased and efficient estimates of T_{excite} and $N_{\text{secondary}}$.

With additional simulation studies, we examined the effect of incomplete observations of the data; that is to say that only a fraction, f_{observed}, of the true number of incidents is actually contained within a particular data set, as would for instance occur if some killings or school shootings did not receive media attention. We found that for such data the estimates of T_{excite} were unbiased and efficient, but that the estimates of $N_{\text{secondary}}$ and N_0 were scaled by f_{observed}. Thus the estimates of $N_{\text{secondary}}$ obtained from the fits to the data samples used in this analysis are lower bounds on the true value.

Simulation of self-excitation contagion processes

To simulate a self-excitation process under some hypothesis of $N_{\text{secondary}}$ and T_{excite}, along with a functional parameterisation of $N_0(t)$, we start with 0 events on day t_1 and calculate the expected number of events on the next day $N^{\text{exp}}(t_2)$ using Equation 1. The number of events on day t_2 is then simulated with a random number drawn from the Poisson distribution with mean $N^{\text{exp}}(t_2)$. On each subsequent day, t_i, the number of events depends on the timing of the past events, and is simulated with a random number drawn from the Poisson distribution with mean $N^{\text{exp}}(t_i)$. This is repeated for the desired length of the time series.

Running mean estimation of baseline number of incidents per day

In order to obtain an estimate of the temporal evolution of the baseline (non contagion-related) number of events per day, $N_0(t)$, from the data itself, we assume that the changes in $N_0(t)$ occur on a relatively longer time frame compared with the changes due to the self-excitation process, and employ the approach of Reference [1] using a Gaussian kernel weighted running mean of the data, with weights that decline as one moves away from the time point of interest. Given a set of events, X_i, observed at times s_i with $i = 1, ..., Q$, the estimate of the average number of events per day not due to self-excitation, N_0, at time t is obtained from the running mean of all points in the time series that are less than Δt days from time

t,

$$N_0(t) = \frac{1}{\sigma\sqrt{2\pi}} \sum\nolimits_{\forall i \text{ s.t. } |s_i - t| < \Delta t} X_i e^{\frac{-(t-s_i)^2}{2\sigma^2}} \tag{3}$$

where σ is a bin-width parameter. The parameter Δt must be chosen to be large enough that points close in time to t that may be due to self-excitation do not bias the estimate of N_0. Similarly the bin-width σ must also be chosen to be large enough to avoid similar bias (and include enough data events within the bin-width in order to ensure accurate estimates of the true value of $N_0(t)$), yet small enough that the running mean provides unbiased estimates the short-term changes in the temporal evolution of the baseline process. Using simulated data sets similar to the size of the sets used in these studies, with a simulated self-excitation process with various hypotheses of T_{excite} between 7 to 28 days and $N_{\text{secondary}}$ between 0 to 0.5, we found that $\sigma = 365/2$ days and $\Delta t = 30$ days yielded unbiased and efficient estimates of $N_{\text{secondary}}$ and T_{excite} for simulated samples of the same sizes and roughly the same temporal trends as the data sets.

We cross-checked our studies by repeating all of the fits with $\sigma = 365$ and $\sigma = 365/4$ days, and $\Delta t = 15$ and $\Delta t = 45$ days, and found no significant difference in the central values returned by any of the fits.

Model validation

To ensure that the model reliably detects when no contagion is present, and to ensure that day-of-week or seasonal effects do not spuriously make it appear that significant contagion is evident when none in fact is, we generated 100 simulated samples under the null hypothesis of no contagion, with the samples having similar size and similar weekday and seasonal variation as the Brady Campaign school shooting data set (this particular data set exhibited the most extreme variations by weekday and season of the three data sets considered in this analysis); from this data set we determined the average number of events occuring within month and weekday, and used this as our expected model.

The contagion model fit to these samples yielded values of $N_{\text{secondary}}$ consistent with zero in 98 out of the 100 trials.

In addition, in order to ensure that the contagion model has good predictive power for our data, for each data set we perform 100 bootstrap iterations where half of the sample is randomly selected as the

training sample and the remainder is selected as the test sample. The parameters of the self-excitation model in Equation 1 are fit to the training data, and the parameters are then used to calculate the likelihood for the test sample via Equation 2. This likelihood is then compared with the likelihood calculated from the null (no contagion) model. If the contagion model has predictive power, the full model will yield a likelihood greater than the null model for the test sample more than 50% of the time.

For the USA Today data, the full model with parameters fit to the training sample is more likely than the null model for the test sample 68% of the time.

For the Brady Campaign school shooting data, the full model with parameters fit to the training sample is more likely than the null model for the test sample 79% of the time. For the shooting incident data the full model with parameters fit to the training sample is more likely than the null model for the test sample 92% of the time.

For the Brady Campaign mass shooting data, the full model with parameters fit to the training sample is more likely than the null model for the test sample 58% of the time.

Other cross-checks

In addition to model validation, we perform cross-checks for each data sample where the dates of each incident are randomly shifted by time ΔT, where ΔT is sampled from the discrete uniform distribution from -90 to $+90$ days. Simulation studies show this shift is large enough to destroy any self-excitation effects with $T_{\text{excite}} < 90/3$, yet is short enough to still preserve the overall temporal shape of the kernel weighted running mean of the sample (thus confirming that the overall temporal distribution of the data is not in itself responsible for potential spurious evaluation of significant contagion when in fact none is present). For all three data samples, this cross-check yielded values of $N_{\text{secondary}}$ consistent with zero.

References

1. Lewis E, Mohler G, Brantingham PJ, Bertozzi AL. Self-exciting point process models of civilian deaths in Iraq. Security Journal. 2011; 25: 244–264.
