# Supplementary material for: Contagion in Mass Killings and School Shootings
Source: PLoS One. 2015 Jul 2;10(7):e0117259. doi: 10.1371/journal.pone.0117259 (PMC4489652; doi:10.1371/journal.pone.0117259)
Supplement: S1 Data — (GZ) [file pone.0117259.s002.gz › data/brady_mass_shooting_to_jan_2013.pdf]

Brady Campaign
To Prevent Gun Violence
SENSIBLE GUN LAWS SAVE LIVES

Mass Shootings in the United States Since 2005

The Brady Campaign compiles this list of mass shootings based on online reports of shootings across the U.S where 3 people or more were injured or killed. Each entry lists the city, state and date of the incident, brief details on the shooting, and the source of the information.

Phoenix, AZ
January 30, 2013
3 people were shot when 70 year old Arthur Douglas Harmon opened fire outside an office building before fleeing. One man was killed and another remains on life support. The body of the shooter was found by police the next day from an apparent self-inflicted gunshot wound. Police have speculated that motive is tied to litigation between two of the victims and the shooter. (*Phoenix office shooting claiming second victim,* **USA Today, January 31, 2013**)

Houston, TX
January 22, 2013
According to police, 22 year old Trey Foster got into a heated argument on Lone Star College's North Harris campus after another individual bumped into him. The argument, which escalated to Foster unleashing gunfire, resulted in the hospitalization of four individuals. Three of the four who were injured appeared to have been wounded by the gunfire, including a maintenance worker who was shot in the leg. (4 Hospitalized in Shooting at Lone Star College, NYTimes, January 22, 2013)(Charges dropped against one suspect in Lone Star College shooting, LATimes, January 28,2013)

Albuquerque, NM
January 19, 2013
A family of five was killed after 15-year-old Nehemiah Griego fatally shot his parents and three younger siblings in their home, using an AR-15 rifle. Griego is alleged to have taken the weapons used from his parents' closet. According to statements, the teen planned on reloading before taking the weapon to a more populated area to kill at random till he was taken out by law enforcement. Griego had no previous interaction with the Juvenile justice system. (Nehemiah Griego, Teen, Allegedly Shoots Parents and 3 Children in Albuquerque, **HuffPo**, **January 21, 2013**)

Hazard, Kentucky
1/13/13
21-year old Dalton Stidham was charged after shooting his girlfriend, her uncle, and her cousin, in the parking lot of the local community college, Hazard Community and Technical College. The gun found at the scene, believed by police to be the murder weapon, was purchased at a pawn shop earlier that day. At the time of the shooting, approximately 30 students were on campus. Stidham's girlfriend had separated from him that October according to her sister, and had been a student at the Kentucky college. (Dalton Stidham Charged With Murder in Shooting of Near Hazard Community and Technical College, HuffPo, January 16, 2013)

Newtown, CT
December 14, 2012
26 people were shot and killed at Sandy Hook Elementary School, a K-4th grade school. 24-year-old Adam Lanza, whose mother was one of the victims and employed by the school, was the lone gunman. Twenty young students and six adults at the school died. (*26 Dead at Newtown School*, **NBC, December 14, 2012**).

Portland, OR
December 11, 2012
22-year-old Jacob Roberts killed two and injured one before killing himself at a Portland-area shopping mall, which was crowded with thousands of holiday shoppers. The injured victim was a 15-year-old girl, to whom Roberts had no relation. (*Details, but no answers, in Oregon mall shooting*, **CNN, December 13, 2012**)

Fresno, CA
November 6, 2012
Lawrence Jones, a 42-year-old employee of a Fresno, CA – area chicken processing plant opened fire at his workplace, killing two and injuring two others before killing himself. Jones had a long criminal history and was on parole at the time of his death. Later reports indicated that he had been diagnosed with 'explosive disorder.'
(*3 dead, 2 wounded after shooting rampage at California chicken plant*, **Fox News, Nov. 6, 2012**)

Chicago, IL
October 21, 2012
Two men, ages 28 and 30, were killed in a shooting that also wounded a 25-year-old pregnant woman in the city's South Chicago neighborhood. This incident was one of many unrelated shootings that together resulted in 24 wounded in one weekend. (*At least 5 dead, 24 wounded in gun violence over weekend*, **Huffington Post, Oct. 22, 2012**)

Brookfield, WI
October 21, 2012
Radcliffe Haughton shot and killed his estranged wife Zina Haughton and two other women, wounding four others before killing himself, at the salon where she worked. Haughton had a history of harassing Zina, who had filed a restraining order against him on October 8, 2012. This order prohibited Haughton from owning any firearms, yet two days after it was approved, Haughton illegally purchased a .40-caliber semiautomatic weapon, which he used to kill three people and himself.
(*Wisconsin salon shooter's wife told court he terrorized her for years*, **NBC, Oct. 22, 2012**)

Casselberry, FL
October 18, 2012
Three women were killed and one was wounded at a beauty salon by gunman Bradford Baumet, who later killed himself at another location. Police say he was served with a domestic violence injunction October 9, 2012, and had been scheduled to appear in court the day of the shooting.
(*Salon shooting: police ID accused gunman in Fla. salon shooting*, **AP, Oct. 18, 2012**)

Winter Springs, FL
September 30, 2012
Several people exchanged gunfire before a charity motorcycle ride began at a Veterans of Foreign Wars post. Shooters killed two and wounded one, and police confiscated numerous weapons.
(*Shooting at veterans club in Florida leaves 2 dead, 1 injured*, **AP, Sept. 30, 2012**)

Minneapolis, MN
September 27, 2012
Disgruntled former employee Andrew Engeldinger entered Accent Signage Systems, Inc., and opened fire, killing five and wounding four before killing himself. Among those killed were Reuven Rahamim, the business owner, and a UPS driver.
(*Five dead in Minneapolis shooting rampage*, **Minneapolis Public Radio, Sept. 28, 2012**)

Compton, CA
September 10, 2012
A still-unidentified shooter opened fire on a group of young men gathered behind a Compton apartment building, killing one and injuring two others. The gunman fled on foot, and police say the shooting appeared to be gang-related.
(*Gang-related shooting in Compton kills 1, injures 2*, **ABC, Sept. 10, 2012**)

Old Bridge, NJ
August 31, 2012
23-year-old Terence Tyler shot and killed two coworkers at a Pathmark grocery store, early in the morning before the store opened. The victims were 18 and 24 years old. Tyler used an assault rifle similar to an AK-47 and killed himself with a handgun. He was a former Marine, and police say he might have had a history of depression and mental illness.
(*Employee shoots 2 dead at NJ supermarket before killing himself, police say*, **NBC, Aug. 31, 2012**)

Chicago, IL
August 24, 2012
Eight people were wounded on a single street on the South Side of Chicago in a drive-by shooting. The victims ranged in age from 14 to 20 years. This incident was among several unrelated shootings the weekend of August 24 that wounded a total of 19 people.
(*At least 19 wounded by gun violence Thursday, 13 over 30 minutes*, **AP, Aug. 24, 2012**)

Laplace, LA
August 16, 2012

Two police deputies were killed and two were wounded in a shootout in a suburb of New Orleans between police and suspects with ties to violent anarchists. The shooters were among seven “sovereign citizens” arrested, known to the FBI for their suspicious activity and radical ideologies. The group of suspects had been previously monitored, and a stockpile of weapons was found in their residences.
(*Suspects in deputy killings linked to extremists*, **CBS, Aug. 18, 2012**)

College Station, TX
August 13, 2012
A 30-minute shootout near the Texas A&M University campus resulted in the deaths of a police constable, a bystander, the shooter, and the injury of four others. It was said by his family that 35-year-old gunman Thomas Caffall was “ill.” The University activated its emergency “Code Maroon” to warn students to avoid the area.
(*3 killed in shootings near Texas A&M University*, **CNN, Aug. 14, 2012**)

Oak Creek, WI
August 5, 2012
Seven people were killed, including the gunman and three injured at a Sikh temple in a Milwaukee suburb. The FBI is investigating the incident as a “domestic terrorist-type incident.”
(*Gunman, six others dead at Wisconsin Sikh temple*, **CNN, August 5, 2012**)

Aurora, CO
July 20, 2012
Twelve people were killed and 58 were injured in Aurora, Colorado during a sold-out midnight premier of the new Batman movie "The Dark Knight Rises" when 24-year-old James Holmes unloaded four weapons' full of ammunition into the unsuspecting crowd. He detonated multiple smoke bombs, and then began firing at viewers in the sold-out auditorium, Ten members of "The Dark Knight Rises" audience were killed in theater, while two others died later at area hospitals. Numerous patrons were in critical condition at six local hospitals, the Aurora police said. (*Colorado Movie Theater Shooting: 70 Victims The Largest Mass Shooting*, **ABC**, **July 20, 2012**)

Tuscaloosa, AL
July 17, 2012
A gunman stood outside of a crowded downtown bar and opened fire from two different positions early Tuesday, sending patrons running or crawling for cover. At least 17 people were hurt. Nathan Van Wilkins, 44, surrendered about 10 hours after the shooting near the University of Alabama campus, police said. Authorities believe one of the bar patrons was a target of the rampage and that it was connected to an earlier shooting at a home. (*Alabama shooting suspect surrenders*, **SFGate, July 17 2012**)

Chicago, IL
July 11, 2012
Four youngsters are among the latest victims caught in Chicago’s gun violence epidemic, including two middle school-aged girls who were wounded in a neighborhood park on the Far South Side. (*Gun Violence Leaves 4 More Chicago Youth Wounded*, **CBS Chicago, July 11 2012)**

Dover, DE
July 9, 2012
At a weekend soccer tournament in Delaware three people died and two were wounded. The dead included the tournament organizer, a 16-year-old boy participating in the tournament and one of three suspects alleged to have initiated the deadly violence Sunday afternoon at a park near downtown Wilmington. (*3 dead after gunfire at Del. soccer tournament*, **AP, July 9 2012**)

Chicago, IL
July 6, 2012
Three people were shot, a 19-year-old man was shot in the calf, a 34-year-old man was shot in the back and a 24-year-old man was shot in the thigh. The oldest was taken to Advocate Christ Medical Center in critical condition and the other two were taken to Roseland Hospital, Alfaro said. Someone approached the three on a bicycle and opened fire. (*3 dead, 7 wounded in shootings across city*, **Chicago Tribune, July 9 2012)**

Seattle, WA
July 02, 2012
One killed, five injured at party.
Gunfire erupted at a South Seattle home where a party was underway. Six people were hit with flying bullets as multiple shots were fired. One of the wounded, a 21-year old woman, later died. (*MORE GUN VIOLENCE IN SEATTLE*, **Sky Valley Chronicle, July 2 2012)**

Chicago, IL
July 1, 2012
One man was killed and a man and woman were injured in a shooting that occurred early Sunday in the East Garfield Park neighborhood, police said. A 22-year-old man was shot in the head and pronounced dead on the scene. (*Cops: 1 dead, 2 injured in West Side shooting*, Chicago Tribune, **July 1, 2012**)

Omaha, NE
06/26/2012
Gun violence put three men in the hospital, one of whom has serious injuries. One neighbor said she heard several shots, then went outside to find the victims hit in the hand, arm and leg. (*3 injured in 61st Street shooting*, **MSNBC, June 26, 2012**)

Houston, TX
06/20/2012
Gunfire erupted outside a Houston night club, killing three people and wounding two others as hundreds of confused concert-goers hit the ground in panic, witnesses and police said. 3 people, including one woman investigators described as an innocent bystander, died. (*3 dead, 2 wounded in Houston club shooting*, **CBS 5 News Channel, June 20, 2012**)

Auburn, AL
06/09/2012
A 22 year old man opened fire at University Heights apartments off West Longleaf Drive. The shooting left three men dead, including two former Auburn University football players, and three others wounded. (*Manhunt on for suspect in Auburn mass shooting*, **Dothan Eagle, June 10 2012**)

Oklahoma City, OK
5/23/2012
A 16-year-old boy is in custody and suspected of opening fire after an NBA game on Monday, injuring eight people. Three of the people shot were bystanders, while five had been involved in the group clashes, which revolved around "high school, girls-and-boys stuff". (*Police say 16-year-old shot eight outside NBA game*, **Chicago Tribune, May 23 2012**)

Chicago, IL
5/26-29/2012
Last year Chicago saw four people slain during Memorial Day weekend. This year the city saw 40 shootings and 10 murders over the four-day weekend (*Chicago's Memorial Day Weekend Was Terrible*, **Atlantic Wire, May 29, 2012**)

Seattle, WA
05/30/2012
40-year-old Ian Stawicki entered a Seattle cafe on Wednesday and opened fire, killing four people. He then left Cafe Racer, killing another person during a carjacking before taking his own life. (*Seattle Mass Shooting Latest by a Concealed Handgun Permit Holder*, **Huffingtonpost, June 1 2012**)

Gilbert, AZ
05/01/2012
Four people killed by a former neo-Nazi before he turned the gun on himself. The killer shot and killed his girlfriend and three others, including a toddler as a result of a domestic dispute. (*Armored gunman, 4 people dead in Arizona shooting*, **msnbc, May 2, 2012**)

Tulsa, OK
4/6/2012
Three were killed and two wounded severely early Friday morning in four separate incidents during a span of less than two hours. Two men were arrested in the crimes. All of the victims were targeted while they were out walking, and apparently did not know each other. Hate crime charges were being evaluated. (*Two Men Arrested, Facebook Clues in Tulsa Shooting Spree,* **ABC News, April 8, 2012**)

Oakland, CA
04/02/2012
Seven people were killed and three others wounded in a shooting rampage Monday at an Asian religious vocational school in east Oakland; police later detained the suspected gunman. (*7 Dead, 3 Wounded In Shooting At Oakland Religious School; Gunman In Custody*, **CBS San Francisco, April 2 2012)**

North Miami, FL
03/30/2012
Fourteen people have been shot and two are dead outside a funeral home in South Florida. One of the victims, a 43-year-old man, died at the scene while the other, a 27-year-old man, died at the hospital. A 5-year-old girl was shot in the leg and hospitalized at Jackson Memorial

Hospital along with eleven other victims. *("Grandma, I've Been Shot" Said Girl Hurt In Gang Related Mass Shooting*, **CBS Miami, March 31 2012**)

Waller, TX
03/20/2012
Trey Sesler massacred his mother, father, and brother in their home in Waller, Texas. He was found by police the next day. A high-caliber rifle was used. It later emerged the killer was also planning a Columbine-style massacre at a local high school. (*Man suspected of killing family captured*, **click2houston.com, March 21, 2012**)

Pittsburgh, PA
3/8/2012
A gunman opened fire in the lobby of a psychiatric hospital in Pittsburgh on Thursday, killing one person and wounding seven, including a police officer, officials said. The gunman was also said to have died. (*2 Die and 7 Are Wounded in a Shooting in Pittsburgh,* **New York Times, March 8, 2012)**

Tempe, AZ
3/3/2012
As hundreds of fans waited in line to see Hussle, fights and arguments broke out, police said. They also said some fans were drinking beer in the parking lot. Three men opened fire on rival gang members and fled, police said. All 14 of the people shot are expected to survive, including the two seriously wounded, Lt. Mike Horn said. (*Tempe concert mass shooting: Rapper decries gun violence*, **Tucson Citizen**, **March, 3, 2012**)

Chardon,OH
2/27/2012
T.J. Lane has told police that he took a .22 caliber Ruger pistol and a knife to the school on Monday and randomly fired 10 shots into a group of students sitting at a table, according to prosecutors. Daniel Parmertor, 16; Demetrius Hewlin, 16; and Russell King, 17; were killed. Nick Walczak, 17, remains in serious condition in a hospital with neck and back injuries, and another student has been released from the hospital. (*Suspect in Ohio school shooting charged with murder,* **Washington Post, March 1, 2012**)

Jackson, TN
2/26/2012
Police said one person was killed and 20 others were injured early Sunday in a shooting at a Tennessee nightclub. An apparent dispute between several people at the Karma Lounge in Jackson led to at least three people opening fire, according to a press release from the Jackson Police Department. The club was hosting an after-party for a basketball game between Lane College and Lemoyne-Owen College. (*Police: 1 killed, 20 injured in shooting at Tennessee nightclub,* **Fox News, February 26, 2012**)

Norcross, GA
2/21/2012
Norcross police on Thursday identified the gunman as the 59-year-old Paek, and the victims as Byong Ok Kang, 64; Kum Hi Song, 61; Kum Sook Kim, 57, and Tae Yol Kim, 55. Surveillance camera footage shows Paek walking into the business and talking to one of the victims before shots were fired, police said. Paek is accused of killing his two sisters and their husbands before

turning the gun on himself. (*Witness to Norcross mass shooting: 'It was horrible'*, **Atlanta Journal-Constitution, February 23, 2012**)

Philadelphia, PA
1/10/2012
Police late Wednesday arrested a man after three teenagers were shot dead in an ambush in Philadelphia, according to a report. The Philadelphia Inquirer reported that another teenager was also wounded in the attack Tuesday night. (*3 Teens shot dead in Philly ambush.***US News MSNBC.com, January 12, 2012**)

Grapevine, TX
12/25/2011
On Christmas morning, police have said, a middle-aged man dressed in a Santa Claus suit shot and killed a man, a teenage boy and four women and then himself inside a unit at the Lincoln Vineyards Apartments in the 2500 block of Hall-Johnson Road. The attack was planned, investigators said. (*Police say gunman in Christmas shootings planted gun on victim*. **Grapevine Courier December 28, 2011**)

Bayonne, NJ
12/19/2011
Three people, including an infant, were shot dead Monday night before the shooter turned the gun on himself, authorities told NBC New York. The gunman shot two adults and the infant, then killed himself, based on initial reports from the scene in Bayonne, N.J., Mayor Mark Smith said. (*Officials: 3 adults, infant killed in 'murder-suicide'*. **NBC December 19, 2011***)*

Irwindale, CA
12/16/2011
Robert Scott Lindsay, 53, of Chino Hills and Henry Serrano, 56, of Walnut were killed Friday when Andre Turner, 48, of Norco opened fire in the office with a semiautomatic handgun, then took his own life. Two others, Angela Alvarez, 46, of Glendale, an Edison employee, and Abhay Pimpale, 38, of Montebello, a contract worker, were wounded and in critical condition. (*Edison office shooting victims, killer identified***. LA Times, December 17, 2011**)

Emmington, IL
12/16/2011
Five people, including three children, were found shot to death Friday afternoon at a home in the small town of Emington, IL., NBCChicago.com reported. The bodies of a woman, a girl and a baby were found in the backyard of home. A man and a boy were found nearby. All had been shot, officials said. The names of the victims haven't yet been publicly released. (*3 children, 2 adults shot dead at Illinois home*. ***MSNBC December 16, 2011)***

Cambridge, MA
12/9/2011
Three people have been fatally shot and killed in Cambridge, Massachusetts. The suspected shooter, a retired police officer later committed suicide. (*Victims identified in Cambridge, Mass. triple shooting.* ***NECN December 11, 2011***)

Bay City, TX
11/30/2011

All four of the children wounded in a mass shooting in east Texas have died, police confirmed Thursday. The children's mother was wounded in the shooting Wednesday afternoon and airlifted to a hospital in Houston, according to police. (*4 children dead in Texas mass shooting*. ***CNN December 1, 2011***)

Oakland, CA
11/29/2011
A 23-month-old toddler who was hit in the head by a bullet during a shooting rampage in Oakland has died from his injuries. Lawrence was shot in the head and six other people were wounded on Nov. 28 when three hooded assailants opened fire on a group filming a rap music video at 7th and Willow Streets. At least two people in the group returned fire. (*Hiram Lawrence, toddler shot in Oakland, dies.* **ABC December 9 2011)**

Wichita, KS
11/27/2011
Four people were shot outside a south Wichita gas station early Sunday morning and police are still working to determine what happened. Emergency dispatchers were soon notified shooting victims had arrived at Wesley Medical Center and at Via Christi-St. Joseph hospital. All four are said to be in serious condition with non-life-threatening injuries. (*Police Looking For 5th Suspect In Quadruple Shooting.* **Kakeland News, November 27, 2011**)

Guilford County, NC
11/20/2011
Deputies believe Mary Ann Holder, 36, shot two sons, a niece, a nephew, the girlfriend of one of her sons and Holder's estranged boyfriend before shooting and killing herself. Five of the victims were 17 or younger. All of the children including Mary Ann were killed, the estranged boyfriend is the only survivor of the shooting. (*Pleasant Garden Hit Hard by Sunday Shooting.***Fox8, November 21, 2011**)

Orange County, CA
10/12/2011
Seal Beach police allege that Dekraai, an ex-marine, entered Salon Meritage in Seal Beach, Calif., wearing a bullet-proof vest and opened fire, killing six people immediately. The six victims were declared dead at the scene. Three people were hospitalized with severe wounds and two of them later died, police said. One of the victims was Dekraai's ex-wife the apparent target. (*Seal Beach Massacre: Suspect's Ex-Wife Claimed Abuse.* **ABC, October 13, 2011**)

Anchorage, AK
10/10/2011
Outside J.J Lounge, a bar in Anchorage, two men were killed and two others were injured during a morning shooting. A witness reported that the gunman repeatedly shot another man in the bar's parking lot. The gunman continued to shoot the man point-blank after the victim put his arms in the air in what appear to be a defensive position. (*Anchorage man arrested on assault, weapons charges after bar shooting that killed 2, injured 2.* **Washington Post, October 11, 2011**)

Newark, NJ
10/10/2011

Four people were left wounded after gunshots were fired at a housing complex. The shooting took place in the Kemsco Village complex. Witnesses stated there were around 15 to 20 shots fired at 1 in the afternoon. Police believe the shots were drug motivated. (*Newark Shooting May Be Drug-Related: Cop*s, **NBC New York, October 11, 2011**)

Chicago, IL
10/8/2011
In the span of one night, 3 people were killed and twenty were injured in the city of Chicago. The injured victims include 10 teenagers, including a kid who was accidently shot by a man showing him a gun. Two men and one woman were killed. (**At least 3 dead, 20 injured in Chicago shooting overnight**. *Associated Press October 8, 2011*)

Dallas, TX
10/8/2011
Eight people were left dead after confrontation between rival gangs at Rolls Royce Club. The two gangs are the Garland Gang and Bonton Gang. A local rapper was about to the stage when he began to argue with another man, who took out a gun and shot the rapper three times and then turned his gun to the rest of the crowd (*Eight Dead in Dallas Rap Gang Shooting,* **AllVoice.com October 8, 2011.**)

Cupertino, CA
10/5/2011
Three workers were killed and seven more injured at a cement plant during the early morning. The gunman began shooting during a safety meeting with more than a dozen co-workers. After shooting coworkers, the gunman retrieved an AK-47 he had brought and fled the scene and police began a day long manhunt. (*California Police Search for Gunman in Shooting That Left Three Workers Dead*, **Officer.com October 6, 2011**)

Carson City, NV
9/6/2011
A gunman opened fire killing four and injuring seven at an IHOP. The gunman used an assault rifle on customers before turning the gun on himself. The weapon used had been altered to function as an automatic weapon. The gunman had a record of mental illness. (*Shooter Used Illegally Altered Gun in Nevada IHOP Rampage, Police Say* **myFOXdc.com October 5, 2011**)

Jacksonville, FL
8/28/2011
About 100 people were at a birthday party for two women when shooters opened fire about 8:45 p.m. at a park in the city's Brooklyn neighborhood. Eleven people were wounded in the gunfire. The victims included an 18-month-old toddler and two pregnant women, one of whom lost her 29-week unborn child. (*All But 1 Released From Hospital in Mass Shooting at Jacksonville Party*, **Florida Times-Union, August 29, 2011**)

Philadelphia, PA
8/22/2011
Six people were injured during a shootout -- one critically -- at a local league basketball game attended by 500 people in the city's Kingsessing section. The victims were innocent bystanders who happened to be near the apparent intended target.
(*Six injured In Shooting at Kingsessing Basketball Game*. **Philadelphia Inquirer, August 23, 2011**)

Lynden, WA
8/20/2011
A 15-year-old boy was charged in the shooting of two men and a woman and the stabbing of a third man at the Northwest Washington Fair. The victims were ages 18 to 23.
(*15-year-old arrested in Lynden fair shootings, stabbing.* **The Bellingham Herald, August 22, 2011)**

Kansas City, MO
8/13/2011
At least three youths were injured in shootings on the city's Country Club Plaza, where a melee erupted in which Mayor Sly James was shoved to the ground. The victims were two boys and a girl, ages 13, 15 and 16.
(*Three youths injured in Plaza shootings.* **The Kansas City Star, August 14, 2011)**

Copley, Ohio
8/7/2011
A 51-year-old Ohio man went on a neighborhood shooting rampage, killing seven and leaving victims in three homes before being shot and killed in a gunfight with a police officer and a former officer. The victims included his girlfriend, whom he wounded in one house. He then ran to a neighboring home and murdered her brother and four other people, including two teenagers. Afterward, he chased two bystanders through backyards, shooting to death one outside a home, before entering another house and killing his girlfriend's 11-year-old nephew. He was killed after he left the home.
(Gunman kills seven in Copley Twp. then is killed himself, **Akron Beacon Journal, August 8, 2011*)***

Auburn, WA
7/24/2011
A man entered a casino seeking a woman believed to be his girlfriend or wife. He found her dancing with another man and promptly pulled a gun and began firing. He shot seven people including the woman and the man with whom she was dancing. Two of the victims were in critical condition. (*Police 7 wounded in casino shooting near Seattle,* **ASSOCIATED PRESS, July 24, 2011**)

Apopka, FL
7/24/2011
A teenager armed with a handgun opened fire after a fight broke out at a birthday party. The teen fired numerous rounds leaving nine other teens aged between 15 and 19 years old wounded. (*9 wounded in house party shooting in central Fla.,* **ASSOCIATED PRESS, July 24, 2011**)

Stockton, CA
7/23/ 2011
A 15-year-old was killed and eight others were wounded during a late night party in a backyard. The unidentified shooter is believed to have entered neighbor's backyard and started firing over the fence. (*Teen killed, 8 wounded in birthday party shooting,* **ASSOCIATED PRESS, July 24, 2011**)

Grand Prairie, TX

7/23/2011
A man shot and killed his estranged wife along with four other family members before killing himself during a birthday party for one of his children at a roller skating rink. One of the victims was only 16 years old. Authorities believe the shooting was prompted by ongoing marital problems between the man and his wife. His two children were not physically harmed. (*Police: Father opens fire at roller rink during child's birthday party,* **CNN, July 24, 2011**)

Grand Rapids, MI
7/7/2011
A man shot and killed himself during a hostage standoff with police following his shooting rampage that left seven dead and two others wounded. The victims included two ex-girlfriends of the shooter and their families. The man killed two young girls aged eleven and twelve, the latter was his own daughter. (*Police say man kills 7 people, including daughter, then himself,* **CNN, July 8, 2011**)

Bechtelsville, PA
7/2/2011
Two couples and a two-year-old boy were all shot in their country vacation home by a lone gunman seeking revenge from an insurance dispute with one of the victims. All five victims were shot in the head. Two were killed, including the infant. Another would die later in the hospital, while the other victims remained in critical condition. The gunman was tracked down by police and killed in a shootout with several officers. (*A Bloodbath in a Country Home, Then a Siege on a Vengeful Man,* **New York Times, July 4, 2011**)

Lauderdale Lakes, FL
6/30/2011
Two men were involved in a dispute over a girl at a crowded nightclub when one of the men pulled out a gun. An armed security guard of the nightclub drew his own firearm and commanded the man to put down his weapon. The man refused and gunfire erupted leaving both armed men dead and ten innocent bystanders wounded by gunshots. (*2 dead, 10 wounded in club shooting,* **WSVN-TV Miami and Ft. Lauderdale News, June 30, 2011**)

Long Island, NY
6/20/2011
At a local pharmacy a man shot and killed four people including an 18-year-old who was set to graduate later in the week. The shooting seems to have been part of a robbery as painkiller medications were stolen. (Alison Fox and Tamer El-Ghobashy, *'Senseless' Killings Link Four Victims,* **Wall Street Journal, June 21, 2011**)

Youngstown, OH
2/6/2011
One person was killed and 11 were injured in a shooting at an after-hours party near the Youngstown State University campus. The deceased was a university student. Six of the injured were also students at the university. Two suspects were arrested. (*2 suspects charged in Youngstown fraternity shooting*, **Cleveland Plain Dealer**, **February 6, 2011**)

West Union, OH
4/30/2011
Four people were found dead at a home in rural southern Ohio. Investigators believe they were shot with a semiautomatic weapon. The victims, all female, were 11, 34, 46 and 68. An 11-year-

old girl who was also in the home was unharmed. The suspect, a 27-year-old man, was living at the home. He had an expired concealed-carry permit. The man was facing felony burglary charges and had failed to appear in court. He died in a shootout with Columbus police. (*4 slain in Ohio; suspect dies in police shootout,* **Associated Press, April 30, 2011**)

Irvington, NJ
3/25/2011
Five men were shot outside a nightclub in Irvington, New Jersey. One of the victims died. The surviving victims are believed to have non-life threatening injuries. The shootings took place around at 4:30 p.m. when witnesses in the area said they heard at least six gunshots. No motive was immediately determined and no suspect was immediately named. (*1 dead, 4 hurt in N.J. bar shooting,* **Associated Press, March 27, 2011**)

Portland, OR
3/20/2011
Police found three men injured at the scene of a Northeast Portland shooting. A fourth victim later arrived at a local hospital with a gunshot wound. Investigators learned that a disturbance began inside a party at a nearby residence. Gunshots were fired inside the residence and outside in the street. (*Portland gang detectives investigate early morning shooting,* **The Oregonian, March 20, 2011**)

Tarpon Springs, FL
3/15/2011
A multiple shooting and suicide left four people dead. The gunman shot and killed his mother, grandson and uncle when he was told he needed to move out of the home in which all four lived. After fatally shooting his three victims, he killed himself. The shooter had been arrested on drug and weapons charges a few days earlier. Police found three handguns at the scene. All three weapons had been used in the killings. (Rita Farlow and Ileana Morales, *Four killed in Tarpon Springs murder-suicide, police say,* **St. Petersburg Times, March 16, 2011**)

Burlington, CO
3/2/2011
A 12-year-old boy was arrested after a shooting that killed a husband and wife and their two children; a five-year-old girl and a nine-year-old boy. The shooter was related to the victims. Burlington is a farming community of about 4,400 residents located near the Colorado border with Kansas. (Kim Posey, *12-year-old accused of killing parents may face life in prison,* **KDVR-TV Fox 31 Denver, March 3, 2011**)

Youngstown, OH
2/6/2011
One person was killed and 11 were injured in a shooting at an after-hours party near the Youngstown State University campus. The deceased was a university student. Six of the injured were also students at the university. Two suspects were arrested. (*2 suspects charged in Youngstown fraternity shooting*, **Cleveland Plain Dealer**, **February 6, 2011**)

Jacksonville, FL
2/5/2011
At least five people were hospitalized in a multiple shooting in northwest Jacksonville. (*At Least 5 Victims in Moncrief Shootings*, **WJXT-TV**, **February 5, 2011**)

Tucson, AZ
1/8/2011
Six people were killed and 13 were injured, including a U.S. Congressman, when a gunman armed with a semiautomatic weapon opens fire at a meet-and-greet with Rep. Gabrielle Giffords outside a shopping center. The gunman, a mentally-ill 22-year-old, shot 32 rounds in 16-seconds before being subdued while trying to reload. (Shailagh Murray and Sari Horwitz, *Rep. Gabrielle Giffords shot in Tucson rampage; federal judge killed,* **Washington Post, January 9, 2011**)

Prince George's Co., MD
1/4/2011
One person was killed and three people were hurt in the Hillcrest Heights/Marlow Heights area. (*5 slayings in 4 days jolt Pr. George's*, **The Washington Post**, **January 5, 2011**)

South San Francisco, CA
12/22/2010
Two people were killed and four others wounded on a street corner in the evening in a drive-by shooting. (Peter Fimrite, *2 dead, 4 wounded in South San Francisco shooting*, **SAN FRANCISCO CHRONICLE, December 22, 2010**)

Sacramento, CA
12/14/2010
A woman was killed and five others wounded in a gun battle at a strip mall. (*1 Dead, 5 wounded in shooting at Sacramento strip mall*, **LOS ANGELES TIMES, December 14, 2010**)

Memphis, TN
12/12/2010
Five men were shot at nightclub early in the morning because someone was “pushing”. (Jody Callahan, *5 men injured in club shooting*, **MEMPHIS COMMERCIAL APPEAL, December 12, 2010**)

St.Louis, MO
11/30/2010
Two people were killed and another two wounded outside a funeral home in an on-going feud between local gangs. (Nicholas Pistor, *Two dead in shooting outside St. Louis funeral home*, **ST.LOUIS POST DISPATCH, November 30, 2010**)

Cleveland, OH
11/28/2010
An argument at a motorcycle club early in the morning lead to five people being shot and one beaten. (Stan Donaldson, *Six people injured after shooting at motorcycle club*, **CLEVELAND PLAIN-DEALER, November 29, 2010**)

Richmond County, GA
11/28/2010
Five people were shot at a club early in the morning. Police believe several people are responsible for the shooting. (*Suspects sought after 5 people shot at club*, **AUGUSTA CHRONICLE, November 29, 2010**)

Bonne Terre, MO
11/24/2010

A man arrived at his ex-girlfriend's apartment and opened fired, killing his ex-girlfriend, her mother and that woman's boyfriend. He shot himself fatally when confronted by officers. An 18-month-old and a 3-year-old were present during the shooting, but left unharmed. (*Police try to learn what spurred Duplex shootings*, **ASSOCIATED PRESS, November 30, 2010**)

Erie, PA
11/13/2010
In an apparent murder-suicide, a man shot his wife, two daughters, and himself to death. (*Parents, 2 kids found shot to death in Western PA*, **ASSOCIATED PRESS, November 13, 2010**)

Atlanta, GA
10/30/2010
Four people were hit in a drive-by shooting at a nightclub during a Halloween party. One of the men and the bartender were shot in the head, and the two other victims were transported to the hospital in stable condition. (*4 Shot in Atlanta Driveby*, **WSB-TV 2 ATLANTA, November 30, 2010**)

Chicago, IL
10/25/2010
A man killed a woman, her nine-year-old daughter, another 16-year-old girl, and her partner's four-year-old son in an apartment. (*3 Dead in Harvey Shooting: Two Children Fatally Shot*, **ASSOCIATED PRESS, October 10, 2010**)

Oklahoma City, OK
10/13/2010
Five people were wounded in a shooting outside two churches. The shooting is thought to be gang related. (Carla Hinton, *One dead, 5 wounded in northwest Oklahoma City shooting*, **THE OKLAHOMAN, October 15, 2010**)

Boston, MA
9/28/2010
Four people were shot to death on a street early in the morning, including a 21-year-old woman and her two-year-old son who was cradled in her arms. The other two male victims were found naked, sprawled on the ground. A third male also lay naked clinging to life after attempting to flee. Law enforcement officials believe the shooting was possibly a drug deal that went bad. (*City, Residents reeling after Mattapan slayings*, **BOSTON GLOBE, September 28, 2010**)

East Orange, NJ
9/25/2010
Five people were shot at an off-campus Seton Hall house party. Students said the shooter was kicked out of the party when he refused to pay the cover charge. (*Witness Describes 'Hell' During Fatal Shooting Rampage at 'Typical Fraternity Party'*, **ASSOCIATED PRESS, September 26, 2010**)

Seattle, WA
9/23/2010
A schizophrenic grandmother armed with a handgun killed her 14- and 17-year-old granddaughters and her 43-year-old son-in-law, and injured her 42-year-old daughter. (Casey McNerthney, *Family: Grandmother in shooting spree battled schizophrenia*, **SEATTLE POST-INTELLIGENCER, September 25, 2010**)

Bay Shore, NY
9/11/2010
After an argument, two people with handguns opened fire, injuring five people outside a bar early in the morning. (Kaitlyn Piccoli, *Five People Shot outside Bay Shore bar*, **Long Island Press, September 9, 2010**)

Chesapeake, VA
9/11/2010
Two people opened fire on a group having a cookout in a park next to a middle school. Four adults and a 14-year-old were injured. (Jennifer Jiggets, *Suspects sought in Chesapeake shooting that wounds 5*, **Virginian-Pilot, September 13, 2010**)

Lancaster, CA
9/6/2010
At least one gunman opened fire at a crowded house party, killing a 14-year-old girl and injuring six others. The injured victims range in age from 13 to 26 years old. (*Detectives seek leads in Lancaster shooting that left girl, 14, dead and 6 others wounded*, **Los Angeles Times, September 6, 2010**)

New Orleans, LA
9/5/2010
A woman was fatally shot in the head and four others were wounded on a street corner. (Rick Martin, *1 killed, 4 hurt in New Orleans shooting*, **CNN, September 5, 2010**)

Durham, NC
9/5/2010
Ten people inside a home were injured in drive-by shooting. (*10 wounded in Durham drive by shooting*, **WRAL - TV 4 Raleigh/Durham, September 10, 2010**)

Chicago, IL
9/2/2010
Four men were fatally shot execution-style in a garage. The men's hands, feet and mouths were bound with duct tape. Multiple weapons were found at the scene. (*Relatives sat slaying of 4 men in garage a mystery*, **WGN- Chicago, September 3, 2010**)

Louisa, VA
8/22/2010
At least four people were wounded and two killed when someone opened fire over a property dispute. When police arrived, the suspect attacked police and they killed him. Several victims were family members of the shooter. (Portia Smith, *Louisa shootout leaves 3 dead*, **Fredericksburg News, August 23, 2010**)

Hartford, CT
8/3/2010
A disgruntled employee, who claimed he had been harassed because he was African-American, opened fire at the beer distribution factory where he worked. He had just attended a disciplinary hearing relating to his theft of beer from the company before the shooting. He killed eight people and injured two before calling his girlfriend and then taking his own life. (Ray Rivera, *Troubles Proceeded Connecticut Workplace killing*, **New York Times, August 3, 2010**)

Indianapolis, IN
8/3/2010
Three men armed with an assault rifle and a handgun ambushed a backyard birthday party and shot seven people. A 37-year-old woman and a 54-year-old man died from their injuries. An eighth person was injured when someone at the party returned fire at the shooters, but shot a bystander instead. (Jon Murray, *Murder charges filed against 3 in gunfight birthday party*, **INDIANAPOLIS STAR, August 21, 2010**)

Ft. Lauderdale, FL
7/30/2010
Five people were shot outside of a nightclub. (Alexa Campbell, *Police identify five victims shot outside Fort Lauderdale Night Club*, **PALM BEACH POST NEWS, July 30, 2010**)

Dallas, TX
7/9/2010
A man killed his pregnant girlfriend, their two kids and himself. He shot their third child, age seven, in the face but she survived. He had a history of domestic violence and the victim had a restraining order against him. (Michael Barrett, *Court records foreshadow day of four deaths in Dallas*, **GASTON GAZETTE, July 12, 2010**)

Richmond, Va
7/3/2010
Police responded to a call reporting random gunfire at a public-housing complex. Officers found five people with gunshot wounds within two blocks. (Jeremy Slayton, *5 wounded in Richmond shooting*, **RICHMOND TIMES-DISPATCH, July 5, 2010**)

East Point, GA
6/26/2010
At least two people attending began shooting when a fight broke out at a large party at an apartment complex. Four people were injured and one was killed. The victims were described as teens or younger adults. (Jay Black, *5 Shot, 1 Dead During Party in East Points*, **WSB NEWS RADIO ATLANTA, June 27, 2010**)

San Bernardino, CA
6/19/2010
A 56-year-old man opened fire in a restaurant, killing his stepdaughter's husband and their six-year-old son before fatally shooting himself in the head. The man also critically wounded his stepdaughter and her 5-year-old son. Authorities said the shooter had a lengthy criminal record that included charges of theft DUI and assault with a deadly weapon. (Victoria Kim, *2 people killed in Del Taco shooting are identified*, **LOS ANGELES TIMES, June 19, 2010**)

Coney Island, NY
6/9/2010
Four people were wounded during a shooting when a dispute involving a man and a 16-year-old girl turned violent. The girl’s brother came outside to stop the fight, and gunshots were fired. The girl, her brother, and mother were all wounded in the incident. (Anahad O’Connor & Colin Moynihan, *4 Wounded in Coney Island Shooting*, **NEW YORK TIMES, June 9, 2010**)

Hialeah, FL

6/6/2010
A Cuban immigrant, who had a clean record in the US, but had served time in prison in Cuba, opened fire at the restaurant where his wife worked. He shot seven women, killing four, before turning the gun on himself. The wife's family claims he repeatedly abused her. (*Shooter Had Violent Past*, **WSVN- TV 7 MIAMI, June 11, 2010**)

New Brighton, MN
6/6/2010
A gunman demanded money from people attending a three-year-old's birthday party a community shelter at a trailer park, and then opened fire, injuring four people. (*4 injured in New Brighton shooting, suspect still at large*, **KARE - TV 11 MINNEAPOLIS, June 6, 2010**)

West Memphis, AK
5/20/2010
A man and his 16-year-old son opened fired with assault weapons on police officers during a traffic stop, and then again in a shootout when police tried to apprehend them after the first shooting. The two officers involved in the traffic stop were killed and two officers were injured during the second shootout; the two suspects were killed by police in the second shootout. The adult suspect had multiple run-ins with the law, and was consider a threat to law enforcement before the shooting happened. (Zack McMillin and Marc Perrusquia, *2 police officers fatally shot in West Memphis, Arkansas*, **MEMPHIS COMMERCIAL APPEAL, May 22, 2010**)

Tuscaloosa, AL
5/16/2010
A man fired a handgun at a graduation party where there were an estimated 100 people in attendance. Nine people were struck by the gunshots, two of which were wounded critically and one was killed. Witnesses said the shooter got into a physical fight with another group, possibly involving gang affiliations. (Stefanie Taylor, *Three shooting victims were friends of suspect*, **TUSCALOOSA NEWS, May 18, 2010**)

North Richmond, CA
5/13/2010
Three men were killed and one man was injured in a drive-by shooting. Two of them men were pronounced dead at the scene and a third died after being taken to the hospital. (*3 killed, 1 hurt in Richmond shooting,* **San Francisco Chronicle, May 13, 2010**)

Detroit, MI
5/3/2010
Five officers were shot, one fatally, when they responded to a report of gunshots at a vacant duplex where drugs were reportedly sold. (*Michigan: 5 Officers Are Shot, One Fatally*, **NEW YORK TIMES, May 3, 2010**)

Decatur, GA
4/15/2010
Two people died and three others were injured when gunfire erupted in a home. Police believe multiple people fired guns during the incident. (*Multiple Shooting near Atlanta, 2 dead*, **ASSOCIATED PRESS, April 16, 2010**)

Chicago, IL
4/14/2010

Four people were killed, including three children and two others were injured at their home in an early morning shooting. A suspect is thought to be related to the victims. (*4 dead, 2 wounded in Chicago shooting*, **ASSOCIATED PRESS, April 14, 2010**)

Muskogee, OK
4/10/2010
An argument between two gangs lead to a shooting at a crowded mall. A 17-year-old was killed and five others were injured. (Deon J. Hampton, *Shooting at mall in Muskogee kills teen*, **TULSA WORLD, April 11, 2010**)

Long Beach, CA
4/5/2010
A gunman opened fire on a group outside an apartment complex, injuring four people, including two teenage girls. Police believe the shooting may have been gang-related. (Joel Rubin, *4 people injured in Long Beach shooting*, **LOS ANGELES TIMES, April 5, 2010**)

Omaha, NE
4/4/2010
A 19-year-old was charged with shooting four other teenagers on a street corner in the evening. (*Arrest Made in Multiple Shooting*, **WOWT-TV 6 OMAHA, April 13, 2010**)

North Hollywood, CA
4/3/2010
A gunman opened fire at a restaurant, killing four people and injuring two others. Police believe the victims were targeted and Armenian gangsters are responsible. (*Restaurant Shooting: Armenian Gangs Blamed for "Blood Bath" at L.A. Restaurant*, **KTLA- TV LOS ANGELES, April 5, 2010**)

Chicago, IL
4/2/2010
Four people were shot on the street after midnight. As emergency responders were taking the victims away, someone shot at police at the scene and injured two bystanders. The shooting is believed to be gang related. (Pat Curry, *Six shot on South Side*, **CHICAGO TRIBUNE, April 2, 2010**)

New Orleans, LA
3/31/2010
An elderly man fatally shot his wife, her sister, and her mother inside their home. He then turned the gun on himself. He survived his attempted suicide. The man had recently been in court charges of domestic violence but was acquitted. (Ramon Vargas, *Treme triple-murder suspect's poor health a factor in domestic abuse acquittal,* **TIMES-PICAYUNE, March 31, 2010**)

Aransas Pass, TX
3/31/2010

A man shot five people in a mobile home. A 35-year-old woman died from her injuries. (Elizabeth Noyola, *Shooting in Aransas pass kills 1, 4 others hospitalized: Police search for killer*, **KZTV – TV 10 CORPUS CHRISTI, April 1, 2010**)

Washington, DC
3/30/2010
A 14-year-old and three men with an AK-47 assault rifle and two handguns opened fire on a group of young adults gathered outside in the evening. Four teenagers, aged 16, 17, 18, and 19, were killed and five other people were injured. Police believe the shooting may have stemmed from a missing a bracelet or was retaliation for a previous shooting. (Clarence Williams, Keith L. Alexander & Paul Duggan, *D.C. police trace shootings that killed 4 through chain of events starting with a man's missing bracelet*, **WASHINGTON POST, April 1, 2010**)
Chattanooga, TN
3/27/2010
Three people, including a juvenile, are suspected of shooting five people, including three juveniles, at Coolidge Park. The shooting happened as police were clearing the park of the hundreds of people who had gathered at the park. (*3 arrested in shooting at park in eastern Tennessee*, **ASSOCIATED PRESS, March 28, 2010**)

New Orleans, LA
3/25/2010
A woman, her seven-year-old daughter, four-year-old son, and 17-year-old sister were each shot multiple times in their home. All four victims died. (Ramon Antonio Vargas, *Mother, her two children and sister in grisly slaying mourned by family members*, **NEW ORLEANS TIMES-PICAYUNE, March 26, 2010**)

Milwaukee, WI
3/21/2010
A man shot four people, including a juvenile and a pregnant woman, outside a house early in the morning. The victims and shooter had argued earlier. The pregnant victim delivered her baby, who was also wounded in the shooting, at the hospital. (Mark Johnson, *Baby born wounded as shooting injures 5*, **MILWAUKEE JOURNAL SENTINEL, March 21, 2010**)

Chicago, IL
3/18/2010
Four people, including a 14-year-old boy, were shot on the street at night. (*4 people wounded in West Side shooting*, **CHICAGO TRIBUNE, March 18, 2010**)

Winnsboro, SC
3/17/2010
Two men shot four people during a robbery-attempt. The homeowner fought the burglars who fired at several guests. (Tony Santaella, James Gilbert & Lauren Eleazer, *4 People Shot in Winnsboro, Suspects in Custody*, **WLTX –TV 19 COLUMBIA, March 18, 2010**)

Pomona, CA
3/13/2010
A gunman opened fire on a group people at an outdoor party in the courtyard of an apartment complex. Three men and two women were wounded. (Corina Knoll, *Shooting in Pomona leaves 5 wounded, police say*, **LOS ANGELES TIMES, March 14, 2010**)

Detroit, MI
3/11/2010
A gunman in an SUV opened fire with an assault weapon at another car, hitting all four passengers. One man died and a two-year-old boy was critically injured. (Zlati Meyer, *Man dead, toddler hurt in Detroit shooting*, **DETROIT FREE PRESS, March 12, 2010**)

New York, NY
2/22/2010
A man shot his wife and two teenage daughters to death with an assault rifle before shooting and killing himself. He and his wife had been having marital problems for a while before the shooting. (Daniel Edward Rosen, Wil Cruz & Edgar Sandoval, *Queens father fatally shoots wife, daughters, then turns gun on self; leaves note that he's 'sorry'*, **NEW YORK DAILY NEWS, February 22, 2010**)

Philadelphia, PA
2/21/2010
One man died and three others were injured after being shot outside a bar during a fight between two people. (Derrick Nunnally, *1 dead, 3 hurt in shooting at N. Phila. Bar*, **PHILADELPHIA INQUIRER, February 22, 2010**)

Huntsville, AL
2/12/2010
A professor opened fire 50 minutes into a Biological Sciences Department faculty meeting at the University of Alabama, killing three colleagues and wounding three others. The shooter had a history of violence: she shot and killed her brother in 1986, was possibly involved in an attempted mail bombing, and was convicted of disorderly conduct, and assault and battery in an incident in 2002. The gun used in shooting was bought from the shooter's husband in 1989 by a friend in New Hampshire to avoid a waiting period in Massachusetts. (Emanuella Grinberg, *Judge sends Alabama university shooting case to grand jury*, **CNN, March 24, 2010**. Aaron Cooper & Brooke Baldwin, *Police: University shooting suspect was charged after fight in 2002,* **CNN, February 17, 2010**)

Miami-Dade County, FL
1/24/2010
Four men pulled up outside an apartment building and opened fire on a group there, killing a 15-year-old girl and injuring three other people. The men were armed with an AK-47 assault rifle and a shotgun. (Jennifer Lebovich, *Police, family seek tips in teen's drive-by shooting death*, **MIAMI HERALD, February 3, 2010**)

Milwaukee, WI
1/21/2010
Five people, including a 20-year-old, were shot outside a tavern. Police recovered shell casings from three weapons. (*5 Hospitalized After Milwaukee Tavern Shooting*, **WTAQ – RADIO WISCONSIN, January 21, 2010**)

Appomattox, VA
1/19/2010
A 39-year-old man shot and killed his sister, her husband, their two children, and four family friends in and around the family home. The man also shot at police with an assault rifle when they arrived at the scene, and even shot a police helicopter six times, bringing it down. The

shooter, who first obtained a concealed carry permit in 1995, and owned dozens of weapons, including “many” AR-15 assault rifles was badly affected by his mother’s death in 2006 and had reportedly become angry about ownership of the family house and land that was left jointly to him and his sister. (Fredrick Kunkle & Josh White, *Man is charged with murder in 8 Appomattox shooting*, **WASHINGTON POST, January 21, 2010**)

Bellville, TX
1/17/2010
A 20-year-old man is charged with shooting and killing his mother, stepfather, sister, brother and two-year-old niece at the family home. Police believe an “ongoing family dispute” prompted the shooting. (*Man charged in southeast Texas family slaying*, **ASSOCIATED PRESS, January 20, 2010**)

Oklahoma City, OK
1/16/2010
Five people were shot at a club, one fatally, late at night. The suspect was on bail and awaiting trial on an armed robbery charge. (*Club Shooting Kills 1, Injures 4: Police Called To El Dorado Club Just Before Midnight*, **KOCO- TV 5 OKLAHOMA CITY, January 17, 2010**)

Hernando County & Dixie County, FL
1/14/2010
A man shot his sister and her coworker to death and injured his niece and an employee. The shooter then fled, shooting and killing a sheriff’s deputy as they pursued him. The shooter had a history of troubling behavior: he had been arrested before and was described as being “hostile toward law enforcement”, was investigated by the Secret Service for comments about President Obama, had attacked his niece and her boyfriend previously, and told family that he wanted to “go on a shooting rampage.” (Karen Voyles, *Death penalty sought in slaying of Dixie captain*, **GAINESVILLE SUN, March 12, 2010**. Lisa Backus, *Fla. massacre suspect drew police attention,* **NEW BRITAIN HERALD, January 18, 2010**)

Kennesaw, GA
1/12/2010
A 60-year-old man wearing camouflage opened fire at a truck leasing company where he used to work. He killed two men and injured three others before fleeing the scene. Four of the victims were employees and the fifth a customer. The shooter reportedly had been laid off in July. (Mike Morris & Alexis Stevens, *Cobb shooter chose victims at random, company official says*, **ATLANTA JOURNAL CONSTITUTION, January, 13, 2010**)

Tulsa, OK
1/11/2010
A man opened fire with an AK-47 assault rifle, hitting four people in a house. One person died. The group had been celebrating the shooter’s birthday when a fight erupted, prompting the shooter to retrieve an AK-47 and fire on fellow celebrants. (Matt Barnard, *Tulsa police: Brawl at birthday party may have led to shootings*, **TULSA WORLD, January 12, 2010**)

Tulsa, OK
1/11/2010
A 31-year-old man was arrested for shooting four people, one fatally, in the evening. The shooting is thought to have been motivated by a quarrel at an earlier social gathering. (*1 dead, 3 wounded, 1 arrested in Tulsa shooting*, **ASSOCIATED PRESS, January 12, 2010**)

West Odessa, TX
1/10/2010
Two 20-year-olds were arrested for shooting five young adults, killing an 18-year-old man early in the morning. All five victims were inside when the shooting started. (*Shooting Leaves One Dead, Four Injured in West Odessa*, **KWES – TV 9 MIDLAND, TX, January 10, 2010**)

Dallas, TX
1/10/2010
Four people were injured when someone in another vehicle opened fire on their car while on the freeway. The vehicles' occupants had gotten into a fight at a nightclub earlier that night. (*Dallas freeway shooting leaves 4 wounded*, **ASSOCIATED PRESS, January 10, 2010**)

New Orleans, LA
1/7/2010
Two men are suspected of shooting and killing three people and wounding a fourth during a home invasion involving narcotics. Two of the deceased victims were shot with an assault rifle; the third was shot with a handgun. (Leslie Williams, *New Orleans police arrest suspect in St. Roch triple homicide*, **NEW ORLEANS TIMES-PICAYUNE, January 12, 2010**)

St. Louis, MO
1/7/2010
A 51-year-old man opened fire at the ABB Power plant where he worked, killing three co-workers and injuring five other, before killing himself. The shooter had sued his employer over their pension fund in 2006 and the trial began just this week. The shooter fired over 100 rounds and was armed with an assault rifle, a shotgun, and two handguns. (Liz Robbins, *Gunman Kills 3 Co-Workers in St. Louis Factory and Then Himself*, **NEW YORK TIMES, January 7, 2010**. Malcom Gay & Liz Robbins, *Details Emerge on St. Louis Shooting, but Motive Unclear,* **NEW YORK TIMES, January 8, 2010**)

New Orleans, LA
1/7/2010
Four people were shot, three fatally, just before midnight. Three victims were found inside the house, and the last was found in the backyard. (*3 Dead, 1 Injured in Bywater Shooting*, **WDSU – TV 6 NEW ORLEANS, January 8, 2010**)

Butler, MO
1/1/2010
A 26-year-old man shot a 29 year old woman and her 25 year old brother to death at her home early in the morning . The woman's seven year old daughter was injured by a bullet fragment. The shooter fled the scene, but police found him several hour later dead by a self-inflicted gunshot wound. The shooter was known to be friends with the victims. (Karen Dillion, *Brother, sister shot to death; police say suspect took his own life*, **KANSAS CITY STAR, January 1, 2010**)

New Orleans, LA
12/24/2009

Five teenagers and a 50 year old man were shot on the street. Police are looking for three suspects. (*Police Seek 3 Men In Multiple Shooting*, **WDSU – TV 6 NEW ORLEANS, December 27, 2009**)

New York, NY
12/17/2009
A man shot four members of a family in their apartment, before falling out a window while trying to escape. The woman is expected to survive, but her husband, father, and son all died. Police believe the shooter had been in jail with the husband, and believe the killings were either drug-related or a robbery attempt. (Al Baker, *Four Men Dead in West Side Shootings*, **NEW YORK TIMES, December 18, 2009**)

San Clemente, CA
12/14/2009
A 38 year old woman, her two young daughters, and their grandmother were all shot to death in a murder-suicide. Police are not sure who pulled the trigger, but the gun belonged to the grandmother. The incident happened shortly after a judge gave temporary custody of the two girls to another relative. The family had come to the area from Houston for the custody hearing. (Ruben Vives, *Grandmother's gun used in murder-suicide*, **LOS ANGELES TIMES, December 17, 2009**)

Mattawa, WA
12/14/2009
A 24 year old man is charged with shooting four men, one fatally. The shooter was reportedly angry about an alleged rape. (*Man charged in Washington state triple homicide*, **ASSOCIATED PRESS, December 17, 2009**)

Grand Junction, CO
12/13/2009
A 20 year old man shot five people early in the morning at a party at his neighbor's house. (Amy Hamilton, *Suspect in shootings could face 5 counts of attempted murder*, **GRAND JUNCTION DAILY SENTINEL, December 14, 2009**)

West Palm Beach, FL
12/7/2009
A gunman in a car opened fire on a group of people, hitting four people, one fatally. The victims were part of a small gathering in the front yard of a home. (*1 Killed, 3 Injured In W. Palm Beach Drive-By Shooting*, **WPBF - TV 25 WEST PALM BEACH, FL, December 8, 2009**)

Lakewood, WA
11/29/2009
A multiple felon ambushed four police officers, shooting and killing them as they were sitting at a coffee shop doing paper work before their shifts began. He fled and hid from police for two days before an on-duty cop happened upon him and killed him. The shooter had been in jail just the week before the shooting for assaulting a police officer and child rape, yet was bailed out of jailed even though he had been implicated in a number of other crimes, deemed by psychologists as dangerous, and had been in jail in Arkansas for years before his sentence was commuted. He had told several family members his plans to kill police in the day before the shooting, but nobody reported his comments. (Lewis Kamb, *Clemmons' last days: A timeline of tragedy*, **TACOMA NEWS TRIBUNE, December 2, 2009**)

Osage County, KS
11/28/2009
A man shot and killed his estranged wife, her grandmother, and their two teenage daughers while they were at home. Their 10 year old son was present but uninjured. The shooter had domestic assault charge pending against him for assaulting his wife. (John Hanna, *Kan. woman, 89, dies after shooting; toll up to 4*, **ASSOCIATED PRESS, December 2, 2009**)

Madison, WI
11/28/2009
A man, woman, seven year old boy, and eight year old girl were all shot in their apartment early in the morning. Police believe the shooting was a domestic incident, and one of those wounded was also the shooter. (*Police in Madison say 1 of 4 shot pulled trigger*, **ASSOCIATED PRESS, November 30, 2009**)

Paterson, NJ
11/28/2009
A man shot his estranged wife and two sons, killing the seven year old, as she was dropping them off at her mother's to baby-sit so she could go to work. The wife had a restraining order against him due to earlier abuse. An off-duty police officer, who witnessed the shooting, shot and killed the shooter. (Michael S. Schmidt & Karen Zraick, *New Jersey Man Who Shot Family Had Been Barred From Seeing Wife*, **NEW YORK TIMES, November 29, 2009**)

Brockton, MA
11/28/2009
Four people were shot, one fatally, at a gas station early in the morning. (*23-year-old killed in Brockton shooting, 3 others wounded*, **BROCKTON ENTERPRISE, November 28, 2009**)

Fresno, CA
11/28/2009
At least two gunmen opened fire at a large house party, injuring 12 partygoers. Police believe the shooting to be gang-related. (*A dozen injured at house party shooting*, **FRESNO BEE, November 30, 2009**)

Jupiter, FL
11/26/2009
A man shot seven relatives after spending three hours with them celebrating Thanksgiving. His six year old cousin, two sisters, one who was pregnant, and elderly aunt all died from their injuries. The shooter had a long history of mental illness and troubles with his family. After the shooting, he is reported to have said: "'I have been waiting 20 years to do this." (Kelly Kennedy, *Dad of victim: 4 Thanksgiving killings preplanned*, **ASSOCIATED PRESS, December 1, 2009**)

Valdosta, G
11/15/2009
A shooting at an apartment complex left one person dead and nine people injured after an argument erupted among a large group of people. (*10 people shot, 1 dead*, **ASSOCIATED PRESS, November 15, 2009**)

Flint, MI
11/15/2009

Four people were shot early in the morning in a car. Three died and a 22 year old was seriously injured. (Liz Shaw, *More details on triple homicide in Flint early Sunday*, **FLINT JOURNAL, November 16, 2009**)

Pearcy, AR
11/12/2009
A gumman shot five people to death in a mobile home, then burnt the home down. The suspect later opened fire on police as they tried to arrest him, injuring one officer, before police shot and killed the suspect. (*Authorities shoot, kill suspect in 5 Pearcy deaths*, **ASSOCIATED PRESS, November 20, 2009**)

Montgomery, AL
11/11/2009
Two 18 year olds who were refused admittence to a private party opened fire later that night at the same party. Five people were wounded and a 17 year old boy was killed. (*Montgomery police arrest 2 in connection with fatal shooting at private party*, **ASSOCIATED PRESS, November 13, 2009**)

Tualatin, OR
11/10/2009
A man shot his estranged wife to death and injured two of her coworkers when he opened fire at a drug-testing lab where she worked. The shooter used an assault rifle and a shotgun in his attack before killing himself. His estranged wife, who filed for divorce the week before the shooting, reportedly was concerned about her safety after he bought multiple guns. (Thom Jensen, *Victim's brother says sister feared husband before shooting*, KATU – TV 2 PORTLAND, OR, **November 11, 2009**)

Oklahoma City, OK
11/9/2009
A 28 year old ex-Marine and cage fighter is suspected of shooting four people to death, including two pregnant women, and then setting their house on fire. Guns, shell casings from two handguns, knives, ammunition, drug paraphernalia and marijuana were found in the house. (*Drugs, ammo found at Okla. Home with 4 bodies*, **ASSOCIATED PRESS, November 18, 2009**)

Walterboro, SC
11/9/2009
Three people were killed, including a 20 month old baby girl, and at least five others were injured in a drive-by shooting. The victims were outside their home when the shooting occurred. (Glenn Smith, *Three slain in shooting: 20-month-old girl among victims of drive-by attack in Walterboro*, **CHARLESTON POST AND COURIER, November 11, 2009**)

Reading, PA
11/8/2009
Multiple gunmen fired at least 25 shots into a crowd at an unlicensed bar. Seven people were hit, one fatally. There were about 100 people in the bar at the time of the shooting. (*Man Killed In Reading Shooting Identified*, **WGAL - TV 8 HARRISBURG, PA, November 8, 2009**)

Chicago, IL
11/8/2009

Four people were shot in the middle of the night in an empty lot. (*3 men, 1 woman shot in lot on Near North Side*, **CHICAGO SUN-TIMES, November 9, 2009**)

Vail, CO
11/7/2009
A 63 year old Vietnam veteran with post-traumatic stress disorder opened fire at a bar, killing one man and injuring three others. The shooter was being escorted out of the bar after "acting 'irate' and was bothering customers" when he opened fire. (*Police: 1 killed, 3 injured in shooting near Vail*, **ASSOCIATED PRESS, November 8, 2009**)

Cleveland, TX
11/7/2009
A 43 year old mentally ill man who "was off his medication, hallucinating and growing more violent" shot his girlfriend, her daughter, and his mother to death before killing himself. (*Officials: Man kills 3, self in rural Texas home*, ASSOCIATED PRESS, **November 10, 2009**)

Orlando, FL
11/6/2009
A mentally ill man with a history of violence shot six people, one fatally, at an engineering firm where he had worked before being fired two years earlier. (Shaila Dewan, *Lawyer Cites Mental Illness in Shooting*, **NEW YORK TIMES, November 7, 2009**)

Fort Hood, TX
11/5/2009
A 39-year-old Army psychiatrist opened fire on his fellow soldiers in a building at Fort Hood army base. The shooter, armed with a FN FiveSeveN “cop killer” pistol, killed 13 and shot another 30 people as he fired off 100 rounds in seven minutes. He was incapacitated after being shot four times and seriously injured by responding police. The shooter, a loner who feared his upcoming deployment and strongly voiced his opposition to the wars in Iraq and Afganistan, had been investigated by federal authorities for his communications with a radical Muslim cleric in Yemen and deemed non-violent, but they were not informed of his purchase of guns and ammunition. The shooter had obtained a concealed carry permit in Virginia in 1996 when he was resident there. (Jason Ryan, Pierre Thomas & Martha Raddatz, *How Alleged Fort Hood Shooter Slipped Through the Cracks*, **ABC NEWS, November 10, 2009**. James C. McKinley Jr. & James Dao, Fort *Hood: After years of growing tensions, 7 minutes of bloodshed,* **NEW YORK TIMES, November 8, 2009** Dan Casey, *Fight to expand gun rights is far from over,* **ROANOKE TIMES, NOVEMBER 10, 2009**)

Denver, CO
11/4/2009
A man shot into a crowd of people exiting from a nightclub at closing time, leaving six people injured and one dead. There had been an incident in the bar earlier that night. (Howard Pankratz, *Club Posh shooting victim dies,* **DENVER POST, November 5, 2009**)

Mount Airy, NC
11/1/2009
A 29-year-old felon shot four men, all in their 20s, to death outside a television store with an assault rifle. The shooter knew his victims, but police are uncertain his motive. (Alysia

Patterson, *Ex-con charged in 4 fatal shootings in 'Mayberry'*, **ASSOCIATED PRESS, November 2, 2009**)

Sumter, SC
11/1/2009
Four people were injured in a drive-by shooting that occurred outside a nightclub early in the morning. (Belton White, *Drive-by shooting outside club injures 4*, **SUMTER ITEM, November 3, 2009**)

Salinas, CA
10/14/2009
Two men shot four other men sitting in a car in the afternoon. (Kimber Solana, *Salinas shooting injures 4*, **SALINAS CALIFORNIAN, October 15, 2009**)

Washington, DC
10/13/2009
Five teenagers were shot, two fatally, at an apartment complex. (Hamil R. Harris, *D.C. Drive-By Bolsters Call for More Resources*, **WASHINGTON POST, October 14, 2009**)

Aliamanu, HI
10/13/2009
A man opened fire with a shotgun, hitting five men, during a fight. (Leila Fujimori, *No arrests yet in shooting that injured 5*, **HONOLULU STAR-BULLETIN, October 14, 2009**)

Bridgeport, AL
10/9/2009
A 28 year old man shot four men in the parking lot of bar early in the morning before fleeing the scene. Police say the shooter was armed with two handguns and a shotgun. (David Brewer, *Investigators seek motive in shooting of four men at Bridgeport bar*, **HUNTSVILLE TIMES, October 9, 2009**)

Charlotte, NC
10/4/2009
Seven people were injured in a shooting at an illegal nightclub stemming from a fight that erupted among several patrons. One witness says a man took an assault rifle out of his car and opened fire on the brawlers. (Ely Portillo, *Gunfire riddles illegal club; 7 hurt*, **CHARLOTTE OBSERVER, October 5, 2009**)

Sumter, SC
10/1/2009
Someone opened fired at several people standing outside an occupied house. Two people outside and three people inside were injured, including a one year old girl who was grazed with a bullet. (Joe Perry, *Four injured in shooting: Bullet grazes 1-year-old girl*, **SUMTER ITEM, October 3, 2009**)

St. Louis, MO
9/30/2009
One man died and three were wounded in a shooting at an apartment complex. (*One dead, three hurt in St. Louis shooting*, **ASSOCIATED PRESS, October 1, 2009**)

Mount Airy, MD
9/24/2009
A man shot his wife and two kids before killing himself inside their home. (Angie Cochrun, *Neighbors, friends shocked by death of family*, **MARYLAND COMMUNITY NEWSPAPERS, September 26, 2009**)

Lakewood, NJ
9/24/2009
A man shot four SWAT team members as they entered his house during a drug and gun raid. One officer was critically wounded. (Wayne Parry, *Shoot-out injures 4 officers, suspect in Lakewood*, **PHILADELPHIA INQUIRER, September 25, 2009**)
Decatur, GA
9/22/2009
A 24 year old man armed with a .223-caliber assault rifle shot killed three people, including a 3 year old boy, and injured one person after an argument. The incident took place at the home of the victims who knew the shooter. The shooter has been in prison previously for shooting someone on the same street in 2006. (Megan Matteucci & Marcus K. Garner, *Accused killer of 3 also charged in armed robbery*, **ATLANTA JOURNAL-CONSTITUTION, September 24, 2009**)

Baltimore, MD
9/18/2009
Two gunmen exited a car and opened fire in the middle of the night, hitting four people. (*Police: 2 Sought In Shooting Of 4*, **WBAL - TV 14 BALTIMORE, September 18, 2009**)

Kansas City, MO
9/7/2009
Five people were injured in a drive-by shooting, including three children. (*Drive-By Shooting Injures 5*, **KCTV – 5 KANSAS CITY, September 7, 2009**)

Indianapolis, IN
9/6/2009
A man shot four people, one fatally, in a truck outside a grocery store in the middle of the afternoon. Police believe the victims knew the shooter and had argued before the shooting. (*1 dead, 3 injured in Westside shooting*, **INDINAPOLIS STAR, September 6, 2009**)

Lindenwold, NJ
9/6/2009
At least two gunmen opened fire on a group of people standing outside a home at night, hitting five men. (Barbara Boyer, *5 remain hospitalized in Lindenwold shootings*, **PHILADELPHIA INQUIRER, September 8, 2009**)

Carbondale, IL
9/5/2009
Four people were shot with a handgun in the middle of the night. (Codell Rodriguez, *Five shot in Carbondale: Police probe two separate incidents*, **CARBONDALE SOUTHERN, September 6, 2009**)

Holden, LA
9/5/2009

A man shot his estranged wife, son, pregnant daughter-in-law, and two year old grandson at their home. The wife, son, and grandson all died. The shooter fled in his car, but shot and killed himself when deputies tried to pull him over a short time later. The man threatened his wife previously and she had a restraining order against him. (*2-year-old, 3 others dead in La. domestic incident*, **Associated Press, September 6, 2009**)

Danville, VA
8/31/2009
A retired police officer accidentally fired his gun in a restaurant, hitting himself and four other people with bullet fragments. (Matt Tomsic, *Five people, not three, injured in Monday's accidental shooting*, **Danville News, September 1, 2009**)

Moss Point, MS
8/30/2009
Six people were wounded and one killed in a shooting at a bar after an argument erupted between two patrons. (Amber Craig, *Police still investigating Mississippi shooting that killed one, injured 6*, **Mississippi Press, September 1, 2009**)

Brentwood, NY
8/29/2009
Gunmen sprayed bullets at a group of people gathered on the front lawn of a home during a party, hitting four people. (*4 men wounded in Long Island party shooting*, **Associated Press, August 29, 2009**)

Lawrenceville, GA
8/27/2009
A man shot five people, killing his girlfriend, her 11 year old daughter, a teenage boy, and a second woman, and seriously injuring her four year old daughter at their home. (Kate Brumback, *4 killed, suspect held in Atlanta-area shooting*, **Associated Press, August 28, 2009**. Andria Simmons, *Quadruple killings suspect maintains innocence,* **Atlanta Journal-Constitution, August 31, 2009**)

Salisbury, MD
8/21/2009
Five young adults were shot in an apartment building parking lot. (*5 people shot near apartment complex in Salisbury*, **Associated Press, August 22, 2009**)

Langston, OK
8/16/2009
Four people, none students, were shot in a parking lot at Langston University. (*Suspect In Langston U. Shooting Identified*, **KOCO – TV 5 Oklahoma City, August 16, 2009**)

Inglewood, CA
8/16/2009
Five people were shot at a party at a home. (*5 shot at Inglewood house party*, **Associated Press, August 16, 2009**)

Menomonie, WI
8/16/2009

A 23 year old man shot and killed three brothers standing outside a mobile home, and wounded a fourth person, before shooting and injuing himself. One of the deceased victims was 13 years old. (*3 brothers shot dead in western Wisconsin*, **Associated Press, August 16, 2009**)

Miramar, FL
8/16/2009
Two mothers and their teenage children were shot in a home during a possible robbery. The two teens and one of the mothers died. (Vytenis Didziulis & Jennifer Lebovich, *Mom, son among 3 slain in Miramar home*, **Miami Herald, August 19, 2009**)

Katy, TX
8/15/2009
A man shot and killed his estranged wife's parents and a teenage relative at their home, before shooting and killing himself a short distance away. (Paige Hewitt & Cindy George, *4 dead in apparent murder-suicide in Katy*, **Houston Chronicle, August 15, 2009**)

Washington, DC
8/15/2009
Seven teenagers were shot during a dispute that errupted after an afternoon community event. (Yamiche Alcindor & Martin Weil, *Northeast Shooting Injures At Least 5*, **Washington Post, August 16, 2009**)

St. Louis, MO
8/11/2009
Three men entered a backyard and began shooting at a group of people playing dice, hitting four people. One victim, who was 18 years old, died. (Carolyn Tuft, *Man is killed, 3 people wounded in shooting*, **St. Louis Post-Dispatch, August 12, 2009**)

Roslyn Heights, NY
8/7/2009
A man shot his 17 year old daughter, estranged wife, and mother-in-law, before shooting and killing himself. The shooter had argued with his family earlier in the day over their plan to move away. The daughter and mother-in-law both died. (Andrew Strickler, *Cops: Man who killed 2 family members, self left note*, **Newsday, August 8, 2009**)

Buffalo, NY
8/5/2009
Five people were shot, two fatally, in a possible attempted robbery. The two people who died were a 19 year old woman and a 25 year old man. (Phil Fairbanks, *City launches Hirschbeck Street cleanup*, **Buffalo News, August 6, 2009**)

Atlantic City, NJ
8/4/2009
Four people were shot, one fatally, in the parking lot of a fast food restaurant early in the morning. (Corn Wilson, *1 Killed, 3 Injured in Early Morning A.C. Shooting*, **WMGM – TV 40 Atlantic City, August 4, 2009**)

Oxon Hill, MD
8/4/2009

Four people were shot, one fatally, in an apartment complex parking lot after an argument erupted in the middle of the night. (Matt Zapotosky, *Man Dies, 3 People Are Injured in Shooting at Oxon Hill Apartment Complex*, **WASHINGTON POST, August 5, 2009**)

Collier, PA
8/4/2009
George Sodini, reported concealed carry permit-holder, attacked a fitness center in suburban Pittsburgh armed with multiple handguns with high-capacity magazines, murdering three women and wounding nine others. He reportedly targeted women specifically in his rampage, and planned the attack in advance. (Lillian Thomas, *LA Fitness shooter had lethal plan*, **PITTSBURGH POST-GAZETTE, August 6, 2009**. Jason Cato, et al., *Survivors of gym carnage relive the terror,* **PITTSBURGH TRIBUNE-REVIEW, August 6, 2009**)

Dyersburg, TN
8/1/2009
A man shot his 15 year old daughter and two neighbors who were acting as her foster parents, before shooting himself. The shooter had been accused of abusing the girl. The girl, foster father, and shooter all died; the foster mother survived. (Travis Loller, *Gunman, Daughter Among 3 Dead in Northwest Tenn.*, **ASSOCIATED PRESS, August 3, 2009**)

Clifton, CO
7/28/2009
Four people were shot at an apartment building. (*Four shot in Clifton; no arrests*, **GRAND JUNCTION DAILY SENTINEL, July 29, 2009**)

Aurora, CO
7/25/2009
Four people were shot, one fatally, outside a private motorcycle club. The shooting took place early in the morning as hundreds of people were leaving the club. At least 30 bullet casings were found at the scene. (Kirk Mitchell, *One killed outside Aurora motorcycle club: Multiple shots were fired as people left motorcycle hangout*, **DENVER POST, July 26, 2009**)

Long Beach, CA
7/24/2009
Five teenagers, aged 14 to 16, were shot while gathered outside a house. Police believe the shooting was gang related. (*5 Teenagers Wounded in Neighborhood Shooting*, **KTLA – TV 5 LOS ANGELES, July 25, 2009**)

Houston, TX
7/22/2009
Six people were shot, including one student, in a drive by shooting at a community rally on the campus of Texas Southern University. Police believe the shooting to be gang related. (Juan A. Lozano, *6 shot, wounded in drive-by at Texas Southern*, **ASSOCIATED PRESS, July 24, 2009**)

Newark, NJ
7/20/2009
Five people sitting on a stoop in the afternoon were shot in a drive-by shooting. One woman died from her injuries. (Victor Epstein, *Three shootings in Newark leave 3 dead, 7 wounded*, **ASSOCIATED PRESS, July 20, 2009**)

Omaha, NE
7/18/2009
A 22 year old died and five others were injured, including a 13 year old boy, after being shot on the street in the middle of the night. (Katie Fretland, *One dead, five injured in shooting*, **OMAHA WORLD-HERALD, July 18, 2009**)

Jersey City, NJ
7/16/2009
Five police officers were shot, one fatally, in a shootout with several suspects. The officer who died had been shot in the face with a shotgun as his SWAT team stormed the suspects' apartment. (Victor Epstein, *NJ police officer dies from shootout wounds*, **ASSOCIATED PRESS, July 21, 2009**)

Bakersfield, CA
7/15/2009
Two men were injured and two men died in a shooting at a home. (*2 dead, 2 injured in Edison Hwy shooting*, **WBAK – TV 29 BAKERSFIELD, July 15, 2009**)

Concord, NC
7/11/2009
A shooting at a street party left one person dead and three others injured. (Cleve R. Wootson Jr., *1 dead, 3 injured in Concord shooting*, **CHARLOTTE OBSERVER, July 12, 2009**)

Austell, GA
7/10/2009
A 20 year old died and three other people were injured after being shot during a home invasion early in the morning. (Rhonda Cook, *Quadruple shooting, 2 murders, suicide baffle Cobb cops: 2 suspects arrests, other sought in crime spree*, **ATLANTA JOURNAL-CONSTITUTION, July 11, 2009**)

New Orleans, LA
7/8/2009
A man got out of a car, and shot at a group of people on the street, injuring four. (Tom Planchet, *Suspect wanted in shooting of 4*, **WWL- TV 4 NEW ORLEANS, July 9, 2009**)

St. Louis, MO
7/7/2009
Gunmen opened fire on a group of more than a dozen people playing basketball. Four people were injured. (John Gadson, *4 People Shot in Drive-By Shooting During Basketball Game: More Than A Dozen Others Ran For Cover*, **KTVI – TV 2 ST. LOUIS, July 7, 2009**)

Overtown, FL
7/6/2009
Several gunmen armed with two pistols and an AK-47 assault rifle opened fire at a block party, killing two people and injuring 10 others. (Jose Pagliery & Jennifer Lebovich, *12 shot at birthday party in Overtown*, **MIAMI HERALD, July 7, 2009**. Jennifer Lebovich, *Second victim of Overtown party shootings die,* **MIAMI HERALD, July 10, 2009**)

Simi Valley, CA
7/1/2009

A distraught man armed with an SKS assault rifle walked into a dentist office, where his estranged wife worked, and opened fire, killing his wife and injuring three other people. (Ruben Vives, *Suspect, victims in Simi Valley dental office shooting identified*, **LOS ANGELES TIMES, July 2, 2009**)

Akron, OH
6/29/2009
Five people were shot, one fatally, in a drive-by shooting. Police believe at least two weapons were used. The shooters and victims had argued before the shooting. (Phil Trexler, *Drive-by shootings puzzling authorities: 1 man dead, 4 others injured. No suspects named, police say*, **AKRON BEACON JOURNAL, July 1, 2009**)

Pico Rivera, CA
6/27/2009
Ten people were shot, three fatally, in a drive-by shooting outside a restaurant where a fundraiser was being held for a motorcycle group. (*Three killed in pizzeria shooting in Pico Rivera*, **ASSOCIATED PRESS, June 27, 2009**)

New Orleans, LA
6/27/2009
Two gunmen emerged from an alley and opened fire on a large group of people, injuring seven of them. (Brendan McCarthy, *13 shot, 3 die during violent weekend*, **NEW ORLEANS TIMES-PICAYUNE, June 29, 2009**)

Kansas City, KS
6/26/2009
A seven year old girl was killed and five adults were injured when someone shot repeatedly into their home from outside. (*Girl, 7, Shot, Killed In KCK Home: 5 Others Wounded In Attack*, **KMBC – TV 9 KANSAS CITY, June 26, 2009**)

Chicago, IL
6/24/2009
Three people were injured and a nine year old girl died after being shot in a drive-by shooting. The girl had been giving her dog a bath outside her father's house when she was killed. (*Three injured, 1 child dead in South Side shooting*, **WLS – TV 7 CHICAGO, June 25, 2009**)

Kansas City, KS
6/22/2009
A man shot and killed four people, including a three year old girl, after a domestic dispute at a home. Each victim had been shot multiple times. The shooter is suspected of beating a man earlier that same day, was involved in another shooting in March, and had a previous criminal record. (*Man held in shooting deaths of 4*, **ASSOCIATED PRESS, June 26, 2009**)

Philadelphia, PA
6/21/2009
A 22 year old man shot seven people, one fatally, at a bar. The shooter had been kicked out of the bar earlier in the night after getting into several fights, only to return later, with a gun, and open fire in the doorway after being barred from entering. (Troy Graham, *Man charged with killing 1, wounding 6 in bar shooting*, **PHILADELPHIA INQUIRER, June 23, 2009**)

College Park, GA
6/15/2009
A gunman with an assault rifle opened fire on a car, injuring all four people inside. (*4 Shot With High-Powered Rifle in Car*, **WSB - TV 2 Atlanta, June 15, 2009**)

Lexington, KY
6/13/2009
Four people were shot, one fatally, outside a convenience store early in the morning. (*Shooting victim released from hospital*, **Lexington Herald-Leader, June 14, 2009**)

Philadelphia, PA
6/5/2009
A man shot four people on their way to a prom party. (*Four shot going to prom party*, **WPVI – TV 6 Philadelphia, June 6, 2009**)

Landover, MD
6/2/2009
A 19 year old woman died at her high school graduation party, and three other people were wounded when someone opened fire at the party in an apartment building. (*Shooting leaves 1 dead, 3 wounded in Landover*, **WTOP - Radio 103.5 FM Washington, June 3, 2009**)

Phoenix, AZ
5/31/2009
A fight turned deadly when a man shot four women, two fatally, in a convenience store parking lot. (Megan Boehnke *2 women slain, 2 wounded in brawl; 3 suspects sought*, **Arizona Republic, June 1, 2009**)

Springfield, MA
5/30/2009
A 24 year old man was arrested for shooting five people, one fatally, during a rampage at a strip club. A sixth person was stabbed during the incident. (Alex Peshkov, *1 dead, 5 hurt in club shooting*, **Springfield Republican, May 31, 2009**)

Birmingham, AL
5/28/2009
A man shot and killed a man and injured two bystanders outside a methadone clinic. Police chased the shooter, who then killed himself. The deceased victim and shooter had both been involved with the same woman. (*3 shot outside Birmingham clinic,* **Associated Press, May 29, 2009**)

Lenoir, NC
5/27/2009
A man shot an eight year old girl, her father, and two sheriff deputies in an incident that began when the shooter killed the girl's dog after it had killed a cat. The shooter was killed in a gun battle with police. (*Dispute over dog leads to fatal shooting,* **United Press International, May 28, 2009**)

Canton, OH
5/27/2009

Three people were shot and killed outside a bar just after closing time. (*Three killed outside Ohio bar,* **UNITED PRESS INTERNATIONAL, May 27, 2009**)

Winton, CA
5/27/2009
Three men opened fire on a group of five men standing outside a home, killing two of them and wounding two others. A woman nearby was also hit. (*2 dead, 3 injured in Winton shooting late Wednesday,* **MERCED SUN-STAR, May 28, 2009**)

Mesa, AZ
5/24/2009
After getting into a fight at graduation party, a man retrieved a gun from his car and shot six people at the party. Two people died. The gunman also shot at police, hitting one officer, when they arrived at the scene. (Sara Pratley, *Two killed in Arizona standoff*, **CNN, May 24, 2009**)

Toledo, OH
5/24/2009
A delusional man went to his neighbors' home and shot the couple who live there and their son, who was visiting. Two children were also in the house, but were not hurt. The shooter believed the neighbors had killed his parents, who live in Michigan, because his mother had not called him yet that day. Police later shot and killed the shooter during a standoff. (Kelly Heidbreder, *Three wounded, shooter dead in Toledo shootout with police,* **WNWO-TV TOLEDO, May 24, 2009**)

Milford, DE
5/23/2009
An 18 year old was arrested for shooting five people after a fight broke out outside of party being held at an American Legion Hall. (*Teen Charged in Multiple Shooting in Milford*, **ASSOCIATED PRESS, May 27, 2009**)

Charlotte, NC
5/20/2009
Four people were injured in a shootout in a strip club parking lot. A man shot at club employees who returned fire, hitting the shooter and his driver. (*3 injured in shootout at strip club,* **CHARLOTTE OBSERVER, May 21, 2009**)

Washington, DC
5/19/2009
A gunman shot four people, including two teenagers, on the street in the daytime. (*Shooting Wounds 4; Officers Hurt in Chase,* **WASHINGTON POST, May 20, 2009**)

Newport News, VA
5/17/2009
Two gunmen shot four people repeatedly before the gunmen fled. Two of the men, ages 18 and 21, died from their injuries.(Alicia Wittmeyer, *Two dead, two injured in Newport News shootout,* **THE VIRGINIAN-PILOT, MAY 17, 2009**)

Hazelwood, MO
5/17/2009

A man shot and killed his ex-girlfriend, her current boyfriend, and himself. Two three year olds and a two year old were in the house when the man killed the couple, but none of the kids were injured. (*Three dead in St. Louis suburb of Hazelwood,* **ASSOCIATED PRESS, May 18, 2009**)

Bakersfield, CA
5/14/2009
Two six year olds, a man and a pregnant woman were shot at a home. The four shooting victims are expected to survive, but the woman lost her baby. Police had been called to the home on two previous occasions in the two months since the family moved in and it is suspected that the shooting was related to domestic incidents. (Steve Swenson, *'Someone really wanted to kill this family,'* **BAKERSFIELD CALIFORNIAN, May 15, 2009**)

Duluth, GA
5/11/2009
Three people were injured and a fourth killed in a shootout over drugs. (Jason Wright, *Shootout leaves four victims, one dead*, **GWINNETT HERALD, May 13, 2009**)

Pendleton, SC
5/11/2009
Four men were shot at a home. (Rick Spruill, *Four shot at Pendleton home in stable condition,* **ANDERSON INDEPENDENT MAIL, May 12, 2009**)

Chicago, IL
5/6/2009
Four people were shot at a grocery store. The suspected gunman was later shot and killed by police after fleeing the scene. (John Garcia, *4 shot, 1 fatally on South Side,* **WLS – TV ABC 7 CHICAGO, May 6, 2009**)

Detroit, MI
5/5/2009
One man died and two were injured when an argument turned into a shootout at a gas station parking lot. More than 40 casings were found at the scene. (*DPD Investigates Spike in SW Shooting,* **WDIV – DETROIT, May 5, 2009**)

Kansas City, MO
5/3/2009
Four people were shot following several skirmishes as the bars were closing in the area. Police arrested three men with an assault rifle. (*Fights, Shooting in Westport leave 4 Hurt*, **KMBC TV KANSAS CITY, May 3, 2011**)

Lakeland, FL
5/2/2009
A man shot and killed his wife, four month old infant, and eight year old son with an assault rifle before fatally shooting himself. He also shot at his 13 year old son, but the boy managed to run away uninjured. (Josh Poltilove, *Dad says son appeared normal before Polk murder-suicide*, **TAMPA TRIBUNE, May 3, 2009**)

Lennox, CA
4/29/2009

A 59 year old man was killed and five people were injured, including a 12 year old and a teenager, in a shooting near a taco truck. (*3 arrested in fatal Calif. Taco truck shooting,* **ASSOCIATED PRESS, April 30, 2009**)

Liberty City, FL
4/26/2009
Four teenagers and a 22 year old were wounded when someone shot at them from a car with an AK-47 assault rifle as they were standing outside a home. (Robert Samuels, *Liberty City home of Brandon Mills targeted in drive-by shooting, 5 wounded*, **MIAMI HERALD, April 27, 2009**)

Washington, DC
4/26/2009
One woman was killed and three were injured after being shot at a home. Police believe the shooter and victims knew each other. (*Police identify woman in Northwest DC shooting,* **ASSOCIATED PRESS, April 26, 2009**)

Jacksonville, FL
4/25/2009
Two gunmen shot 10 people, one fatally, while they were at a neighborhood block party. (*Police search for gunman in block party shooting,* **Associated Press, April 27, 2009**)

Fairhope, AL
4/25/2009
A man shot his estranged wife, her current boyfriend, and her boyfriend's grandparents at the grandparent's home, killing the boyfriend and grandparents. The gunman later opened fire on police, who returned fire and killed him. The gunman was out on bond after being jailed for burglary. (Mark Kent and Franklin Hayes, *Four Shot to Death,* **PRESS-REGISTER, April 27, 2009**)

Athens, GA
4/25/2009
A man shot and killed his wife and two men at a local theater group picnic. The gunman returned to his car after the shooting where his children were waiting, dropped them off at a neighbor's, and then fled to an uninhabited area where he killed himself. The couple had been having marital troubles. (*3 dead, 2 hurt in Georgia shooting; professor sought,* **ASSOCIATED PRESS, April 25, 2009**)

King City, CA
4/22/2009
A 13 year old boy was killed and four others were injured, including two other teenagers, when someone shot them while they played video games in one of the victim's home garage. (*5 Shot in King City, 2 Dead,* **KCBA – TV, April 23, 2009**)

Oak Cliff, TX
4/20/2009
One person died and three were injured after a group of men started shooting into a Hummer parked outside a restaurant. The Hummer passengers returned fire and dozens of rounds were fired in the exchange. Police recovered an AK-47 assault rifle and a semiautomatic pistol from the scene. (Jon Sasser, *Police arrest suspect in fatal restaurant shooting,* **WFAA-TV DALLAS – FORT WORTH, April 20, 2009**)

Detroit, MI
4/17/2009
A 30 year old man died and four other people, including a four year old girl, were injured in a shooting. The deceased victim had been arguing with several men at a car repair shop when at least one man began shooting at him. Several bystanders were also hit, including the child and wife of the shop's owner and an employee. (Ben Schmitt, *1 killed and 4 wounded in shooting outside shop,* **Detroit Free Press, April 18, 2009**)

Warren, OH
4/13/2009
Someone armed with an assault weapon fired repeatedly into a home during a drive-by shooting, killing a child and an adult, and injuring a second child. (*Assault-Weapon Crimes*, **Youngtown Vindicator, April 23, 2009**)

Valdosta, GA
4/12/2009
Five people were shot in the parking lot of a business during an argument. (Kim Carapucci, *Brooks County man arrested and charged or Valdosta shooting,* **WCTV-TV, April 17, 2009**)

Rosemead, CA
4/10/2009
Two gunman opened fire at a party at a hotel, killing a 15-year-old and 27 year old, and injuring three others. (Brian Day, *Two killed in shooting at party,* **San Gabriel Valley Tribune, April 11, 2009**)

Morro Bay, CA
4/8/2009
Four people were shot, one fatally, during a robbery at a home. (*Man killed, three wounded in shootout at Morro Bay home,* **The Tribune – San Luis Obispo County, April 10, 2009**)

Temecula, CA
4/7/2009
A man shot his wife, himself, and two others at a Korean religious retreat. The shooter was a volunteer who lived at the retreat. One person died. (Gene Ghiotto and Sandra Stokely, *Gunnman, victim in Temecula shooting identified,* **The Press-Enterprise, April 8, 2009**)

Graham, WA
4/4/2009
A father shot and killed his five children, aged seven to 16, shooting each multiple times after his wife left him. He later shot and killed himself several miles away in his car. Child welfare had investigated him two years previously for assaulting one of his children, and put him on a "parenting plan." (William Yardley, *Wife was leaving man who killed 5 children,* **New York Times, April 5, 2009**)

Kansas City, MO
4/4/2009
Five people were injured in a drive-by shooting. (*KCK Drive-by Shooting Injures 5,* **KMBC-TV Kansas City, April 5, 2009**)

Binghamton, NY
4/3/2009
A disgruntled man, wearing body armor and carrying copious amounts of ammunition, shot and killed 13 people and wounded four more, before killing himself at an immigrant community center. The shooter, an immigrant himself, was having trouble finding a new job after losing his old one and felt people were mocking his English. He was a gun enthusiast with a few previous brushes with the law. (Katharine Seeley, *Shooting in Binghamton, N.Y.,* **NEW YORK TIMES, April 3, 2009**)

Santa Clara, CA
3/31/2009
A man shot his wife, 11 year old son, and 4 year daughter, as well as his wife's brother, the brother's wife, and their 11 month old baby, before shooting and killing himself. The shooter's wife was the sole survivor of the massacre. (Matthew Stannard, *5 killed 2 injured in Santa Clara shooting,* **SAN FRANCISCO CHRONICLE, March 31, 2009**)

Carthage, NC
3/30/2009
A man shot and killed seven residents and a nurse, and wounded three others at Pinelake Health and Rehab Center, a nursing home where his estranged wife worked. The gunman was shot and incapacitated by a police officer, who was also wounded in the rampage. (*Police: N.C. gunman stopped by single shot,* **Associated Press, March 30, 2009**)

Lakeville, MN
3/29/2009
A man shot four people at a house party after getting upset about being asked to leave. (Chao Xiong, *Suspects jailed after gunshots wound four in Lakeville,* **MINNEAPOLIS STAR-TRIBUNE, March 30, 2009**)

Oakland, CA
3/21/2009
A man with an extensive criminal history shot five police officers, killing four of them. The gunman shot and killed the first two police officers during a routine traffic stop, and then fled to a nearby apartment building where he shot another three police officers with an assault rifle. The gunman died when police returned fire. (Henry K. Lee, *Oakland police funeral set for today*, **SAN FRANCISCO CHRONICLE, March 27, 2009**)

Jacksonville, FL
3/20/2009
One person died and four others were wounded after being shot at a block party at an apartment complex. (*1 Dead, 4 Injured in block Party Shooting,* **WJXT-TV Jacksonville, March 21, 2009**)

Miami, FL
3/15/2009
A man shot and killed his estranged wife, her daughter, her boyfriend, and her mother at the boyfriend's birthday party. The gunman shot over thirty rounds, spraying bullets at other partygoers and the neighborhood. He then returned to his home, set the house and car on fire before shooting and killing himself. (*Five Killed in Miami murder-suicide – police,* **Reuters, March 15, 2009**)

Conover, NC
3/12/2009
A drug trafficker shot and stabbed a woman and her three children to death in their house. When the police tracked him down several days later in Utah, the man shot his girlfriend and then himself to death instead of being apprehended. (*'America's Most Wanted' to cover NC killing of 4,* **Associated Press, March 15, 2009**)

North Richland Hills, TX
3/12/2009
A man shot his girlfriend, her mother, and her 17 year old son before shooting and killing himself. The girlfriend survived and a toddler was not hurt, but the mother and son died from their injuries. (*Shooting in North Richland Hills leaves three dead, one critically injured,* **Pegasus News, March 13, 2009**)

Kinston & Samson, AL
3/10/2009
Michael K. McLendon, 28, killed 10 people over a 20 mile trail in rural southern Alabama. He first killed his mother and the family dogs then drove 20 miles and shot five members of his extended family. Next, his violence became random as he sprayed bullets at cars, stores, and police officers, killing neighbors, a man walking down the street, a woman at a store, and a passing motorist. He was armed with a handgun, a shotgun, and two assault rifles. He grazed one police officer's shoulder, and another officer in pursuit did not know at the time of the shootings that his wife and 18-month-old daughter were among the victims. They were on their porch across the street from where McLendon's extended family lived. Geneva Police Department stated that McLendon was licensed to carry handguns. (*Timeline: 11 dead in one afternoon, two towns reeling*, **CNN, March 11, 2009**, Shaila Dewan & A.G. Saulzberger, *Officials Identify Alabama Gunman,* **The New York Times, March 12, 2009** Jay Reeves, *Killer's march took 200 bullets, 1 hour, 11 lives,* **Associated Press, March 12, 2009**)

Louisville, KY
3/9/2009
Four people were shot on the dance floor of a nightclub. (*Four shot at Louisville dance club,* **Associated Press, March 9, 2009**)

Kansas City, MO
3/7/2009
One person died and three others were injured when they were shot while riding in their car on Interstate 70. (*Police: 1 Dead, 3 Hurt in I-70 Shooting,* **KMBC-TV ACB Kansas City, March 7, 2009**)

Miami Gardens, FL
3/7/2009
Five people were shot outside of a nightclub after an argument inside escalated. (*Five injured during shooting after fight in Miami Gardens,* **Miami Herald, March 9, 2009**)

Portsmouth, VA
3/7/2009

A gunman opened fire on a group of people from his car. Seven people were hit, including four teenagers, a 12 year old, and a five year old. (Matthew Bowers, *'Snakeshot' used in Porstmouth shooting, police say,* **Virginian-Pilot, March 10, 2009**)

Cleveland, OH
3/5/2009
A man shot and killed his wife, sister-in-law, and three young children in his house. A fourth child was also shot but is expected to survive and two other children were not injured. The shooter was previously convicted of manslaughter and assault. He shot and killed himself when police closed in on him the next day. (M.R. Kropko, *Police say 5 dead in Cleveland shooting,* **Associated Press, March 5, 2009**)

Largo, MD
3/1/2009
A man shot seven people, one fatally, outside a bar. An argument between two groups started inside the bar earlier in the night, and then escalated outside. (*1 Dead, 6 Injured in Shopping Center Shooting,* **WUSA-TV Washington D.C., March 2, 2009**)

Catahoula Parish, LA
2/28/2009
Eight people were shot outside a nightclub along a rural highway. There are four suspects, including a 16-year-old and 18-year-old felon. (*Eight Shot at Nightclub,* **Monroe News-Star, March 2, 2009**)

Houston, TX
2/27/2009
A gunman shot three teenagers and an adult, two outside a high school and two at a nearby bus station. (Jennifer Leahy, *3 students, 1 adult shot after school,* **Houston Chronicle, February 27, 2009**)

Miramar Beach, FL
2/26/2009
Two people were killed and three were wounded when a neighbor opened fire on the victims' townhouse. All five victims were from Chile, but working in the US legally. (*Chillean students on break killed in Fla.,* **Associated Press, February 27, 2009**)

South Miami-Dade, FL
2/25/2009
A man shot and killed his wife and two daughters, before killing himself at their home. (*South Dade dad kills wife, 2 daughters and himself; son escapes,* **Miami Herald, February 25, 2009**)

New Orleans, LA
2/24/2009
Seven people, including a toddler and two teenagers, were shot at a Mardi Gras parade in the middle of the afternoon. All survived. (Emily Smith, *7 shot as maniacs mar the Mardi Gras,* **The Sun, February 26, 2009**)

Newark, NJ
2/24/2009

Five people were injured after being shot at an apartment complex over an argument. (Carly Rothman, *Five shot at Newark housing complex,* **NEW JERSEY STAR LEDGER, February 25, 2009**)

Newark, NJ
2/18/2009
Seven people were shot at a nightclub. (Paul Cox, *Six are wounded in shooting at Newark nightclub,* **NEW JERSEY STAR LEDGER, February 19, 2009**)

New Orleans, LA
2/16/2009
Four people were shot in a home during a robbery attempt. (*4 Injured in New Orleans Shooting,* **WDSU-TV NEW ORLEANS, February 17, 2009**)

Brockport, NY
2/14/2009
Frank Garcia, 35, opened fire in the parking lot of a hospital from which he was recently fired. He shot three people there, killing two, and later shot a married couple execution-style inside their home. Garcia was denied a reported CCW license three times due to past arrests for assault, harassment, and criminal possession of a weapon before being granted a license in April 2007. (Brian Sharp & Meaghan M. McDermott, *Slaying suspect denied gun permit 3 times before getting 2007 OK*, **DEMOCRAT AND CHRONICLE, February 19, 2009**)

North Miami, FL
2/13/2009
A man with an AK-47 assault rifle shot at a group of people standing outside an apartment building, wounding four, including an eight year old boy. (Jose Pagliery, *Four wounded in North Miami AK-47 shooting*, **MIAMI HERALD, February 13, 2009**)

Seat Plesant, MD
2/4/2009
A man shot and injured his fiancée and her parents before shooting and killing himself. A two-year-old girl was also present but not injured. The fiancée had filed protective orders against him before, but rescinded it, and another woman had a pending protective order against him as well. (*D.C. firefighter shoots, injures 3, kills self,* **WASHINGTON TIMES, February 5, 2009**)

Los Angeles, CA
1/26/2009
A man shot and killed his wife and five young children, before killing himself. He and his wife had recently lost their jobs. (*LAPD: Dad Who Killed Family Was in Debt, Heading to Kansas,* **ASSOCIATED PRESS, January 29, 2009,**)

Wichita, KS
1/25/2009
Nine people were shot, two fatally, while attending a wake at a private home. (*Police:2 Shot Dead, 7 Hurt in Wichita, Kan, Wake,* **ASSOCIATED PRESS, January 25, 2009**)

Miami, FL
1/24/2009

A man with an AK-47 assault rifle fired into a crowd of people gathered outside to play dice. Nine people were shot, two teenagers fatally. (Andrew Ba Tran, *Two people dead, seven hurt in Miami AK-47 shooting*, **South Florida Sun-Sentinel, January 24, 2009**)

Portland, OR
1/24/2009
A 24-year-old man with a history of mental illness and hospitalization shot nine people at an under-21 nightclub before shooting and killing himself. Two teenage girls died from their injuries. (Mary Hudetz and Ryan Kost, *Portland shooting susopect left note, but no word on motive,* **Associated Press, January 27, 2009**)

Glendale, AZ
1/10/2009
Four men were shot and wounded outside a townhouse during a fight. (Lisa Halverstadt, *4 men in hospital after shooting in Gelndale,* **The Arizona Repulic, January 11, 2009**)

Chicago, IL
1/9/2009
A gunman began shooting indiscriminately from a car into a crowd of people that were leaving a basketball game at Paul Laurence Dunbar Vocational Career Academy. Five people were injured. (Rupa Shenoy, *5 people shot outside Chicago high school*, **Associated Press, January 9, 2009**)

Greenfield, CA
1/4/2009
Two gunmen approached a group barbecuing in a garage with the door open and opened fire, killing two men and injuring four other people. (*2 killed, 4 injured in Greenfield BBQ Shooting,* **KSBW-TV Central Coast, January 5, 2009**)

Dallas, TX
12/30/2008
Three men in masks shot four people, one fatally, at a shop. (*3 Sought in Dallas Store Shooting that Left 1 Dead,* **Associated Press, December 31, 2008**)

Baltimore, MD
12/28/2008
A gunman approached a group of people standing outside and opened fire, wounding five. (*5 Injured in West Baltimore Shooting,* **WBAL-TV Baltimore, December 29, 2008**)

Denver, CO
12/27/2008
Five people were shot outside a club as it was closing. (Jeremy P. Meyer, *Streets bloodied by club gunfire,* **The Denver Post, December 28, 2008**)

Lynchburg, SC
12/26/2008
One person died and three others were injured after they were shot during an argument at a bar in the early morning. (*Fight at club leads to fatal shooting,* **The Post and Courier, December 28, 2008**)

Covina, CA
12/24/2008
A man dressed in a Santa suit entered his ex-wife's family's holiday party and massacred the party-goers. He shot at least eight people, before he doused the house with flammable liquid and started a fire. A total of nine people were killed in the home, while two others survived being shot. The gunman had planned to continue his massacre elsewhere, but unintentional injured himself in the fire, and ended up shooting and killing himself. (*"Santa" Shooting Rampage Leaves 9 Dead,* **ASSOCIATED PRESS, December 25, 2008**)

Trotwood, OH
12/12/2008
A man with a criminal record shot six people, one fatally, in a crowded bar. The shooter had been was thrown out of the bar earlier that night, but later returned with a gun. (Doug Page, *Witness: A scuffle, then gunfire,* **DAYTON DAILY NEWS, December 13, 2008**)

Jacksonville, FL
12/10/2008
A masked gunman shot five people outside a home. (*Teen Girl Among Five Shot in Northwest Jacksonville,* **WJXT-TV JACKSONVILLE, December 10, 2008**)

Atlanta, GA
12/7/2008
Five people were wounded and one person was killed when a gunman opened fired in a crowded nightclub. Firecrackers were set off just before the shooting, which police speculate may have provoked the shooter. (Andria Simmons, *Police seek gunman in American Legion shootings,* **ATLANTA JOURNAL-CONSTITUTION, December 7, 2008**)

Atlanta, GA
12/2/2008
Four people were injured in a shootout over drugs. At least one person was armed with an AK-47 assault rifle. (Christian Boone & Marcus K. Garner, *Four people injured in Atlanta shootout*, **ATLANTA JOURNAL-CONSTITUTION, December 2, 2008**)

Murfreesboro, NC
11/23/2008
A man shot two people, one fatally, at his former company, United Turf, and then went to a former coworker's home where he shot and killed the coworker. The gunman later shot himself fatally. (Jeff Hampton, Jim Washington, Lauren King, *Man shot slef after killing 2, wounding one, N.C. sheriff says,* **THE VIRGINIAN-PILOT, November 25, 2008**)

Skyway, WA
11/22/2008
Four people were shot, one fatally, in a restaurant parking lot over an argument that erupted inside. (*One person killed in Skyway shooting,* **THE SEATTLE TIMES, November 22, 2008**)

Stockton, CA
11/21/2008
A 20-year-old man died and four other people were injured after being shot on a street corner near an elementary school. (Christian Burkin, *South Stockton sees second homicide in less than a day,* **STOCKTON RECORD, November 23, 2008**)

Los Angeles, CA
11/21/2008
A man with a restraining order against him shot his estranged wife, and their seven year old daughter and nine year old son, before shooting and killing himself. The two kids died from their injuries. (Richard Winton, *Man kills children, himself,* **LOS ANGELES TIMES, November 22, 2008**)

Odenton, MD
11/16/2008
Three men armed with handguns ambushed four other men, who were in a car in a mall parking lot, killing two and seriously injuring two. There was an ongoing dispute between the two groups. (Matt Zapotosky, *Two Men Killed, Two Wounded in Shooting at Odenton Mall,* **WASHINGTON POST, November 17, 2008**)

Saginaw, MI
11/16/2008
A man armed with a rifle shot four women as they sat in a car across the street. Three were seriously injured, two were sisters, and one was pregnant. (*Shooting leaves 4 females injured,* **WEYI-TV MID-MICHIGAN, November 17, 2008**)

Dallas, TX
11/16/2008
Five men were shot, one fatally, in a shootout outside a nightclub. The men had been arguing over a woman inside the club. (**source**)

Kansas City, MO
11/10/2008
Four people were shot, one fatally, after an argument turned violent. (*KC police say 1 dead in quadruple shooting,* **ASSOCIATED PRESS, November 11, 2008**)

Columbus, IN
11/9/2008
One person died and three were injured after being shot at a bar. (Ruthanne Gordon and Jackie Carpenter, *1 dead, 3 injured in Columbus shooting,* **WISH-TV INDIANAPOLIS, November 9, 2008**)

Boston, MA
11/9/2008
One person died and four others were wounded in an overnight shooting. (*Mother Dead, 4 Injured in Shooting,* **WCVB-TV Boston, November 9, 2008**)

Monticello, MS
11/8/2008
Three men armed with two shotguns and an AK-47 assault rifle ambushed four men as they drove by in a car, killing one of the men and injuring the other three. (Marsha Thompson, *Lawrence County lawmen say shooting victims ambushed*, **WLBT - TV 3 JACKSON, November 12, 2008**)

Chicago, IL

11/2/2008
Seven people were wounded in a drive-by shooting. The victims, three men and four women, were shot as they were leaving a social club. (*Chicago Police:7 Injured in Drive-by Shooting Outside Cub,* **Associated Press, November 2, 2008**)

Long Beach, CA
11/2/2008
Five people were found shot to death in a makeshift homeless encampment in the woods between two highways. (*Police Seek Leads in Shooting that Left 5 Homeless Dead,* **Associated Press, November 3, 2008**)

Orlando, FL
11/2/2008
Four people were shot, one fatally, overnight at a McDonald's parking lot. (Bianca Prieto, *Police seek clues in deadly McDonald's shooting,* **Orlando Sentinel, November 4, 2008**)

Marrero, LA
10/30/2008
Four people died after being shot during a robbery attempt at a bar. The two owners, who were brothers, were among the dead. (Cain Burdeau, *4 people slain in botched La. Pool hall robery,* **Associated Press, October 30, 2008**)

Floral City, FL
10/30/2008
A mother shot and killed her three young sons, aged four, two, and 15 months, before shooting herself fatally. She and the kids had recently moved out of her boyfriend's home. (*Mom, 3 Young Sons Shot Dead in Florida Home,* **Associated Press, October 31, 2008**)

Stockton, CA
10/25/2008
Five people were shot in the parking lot of an apartment building after leaving a Halloween party. The incident stems from an argument that began at the party. (*Shooting at Halloween party leaves 5 hurt,* **Stockton Record, October 26, 2008**)

Newark, NJ
10/24/2008
Three people drove around Newark and randomly targeted pedestrians during a shooting spree that lasted for almost an hour. They opened fire at seven different locations, killing two people and wounding four, including two high school students. (*Two Men Arrested in Newark Shootings,* **Associated Press, October 25, 2008**)

West Hartford, CT
10/18/2008
Four people were shot at a baby shower when a fight erupted and turned violent. (*Connecticutt Police Hunt for Motive After Baby Shower Shootout,* **Associated Press, October 20, 2008**)

Detroit, MI
10/16/2008

Three teenagers shot four other teenagers near Henry Ford High School over a fight at school. A sixteen year old died from his injuries. (*3 teens charged in Detroit school shooting,* **ASSOCIATED PRESS, October 19, 2008**)

North Vallejo, CA
10/15/2008
A man on a motorcycle shot three men and a two year old boy who were outside a home. The gunman hit all four victims multiple times, shooting repeatedly with a high-capacity handgun. (Shauntele Lowe, *North Vallejo shooting victims remain hospitalized; suspect at large,* **THE OAKLAND TRIBUNE, October 16, 2008**)

Grand Junction, CO
10/11/2008
A man shot two people outside their home after talking with them and then shot two passersby. Later the gunman shot himself. One person died from his injuries. (*Two killed in Grand Junction shooting,* **ASSOCIATED PRESS, October 12, 2008**)

Columbus, OH
10/9/2008
Three men and a woman died after each was shot multiples times while inside a home. (Theodore Decker, *Police call Hilltop killings executions,* **THE COLUMBUS DISPATCH, October 10, 2008**)

Glenarden, MD
10/4/2008
Four people were shot, one fatally, while standing outside an apartment complex. (Natalie McGill, *Glenarden shooting kills one, injures three,* **GAZETTE.NET, October 6, 2008**)

Columbia, SC
9/28/2008
A 19-year-old shot four people at a club. None of the injuries were fatal. (**source**)

New Haven, CT
9/27/2008
Four people were injured after being shot while attending a memorial service for a man killed by gunfire the previous week. (**source)**

Phoenix, AZ
9/23/2008
A gunman entered a home and shot four people, killing a 17-year-old. (Parker Leavitt, *Teen killed, 3 others injured in Phoenix shooting,* **THE ARIZONA REPUBLIC, September 24, 2008**)

Compton, CA
9/23/2008
Five people were shot, two fatally, near a bus station. (*Two killed in shoopting at bus stop,* **THE LONG BEACH PRESS-TELEGRAM, September 24, 2008**)

Grand Rapids, MI
9/19/2008

Five people on a porch were wounded when a man with a shotgun fired at them from a passing car. (*Five injured in Grand Rapids shooting,* **WZZM-TV GRAND RAPIDS, September 22, 2008**)

Columbus, OH
9/19/2008
Two people were wounded and two people were killed in a shooting. (Theodore Decker, *Two dead, two critical in East Side shooting,* **THE COLUMBUS DISPATCH, September 19, 2008**)

Philadelphia, PA
9/11/2008
Two gunmen attacked an award ceremony for a basketball league, opening fire into the crowd assembled at a playground. They shot five people, two died, including a 19 year old. (*Two Gunmen at Large in Philadelphia After Deadly Youth Basketball Ceremony,* **ASSOCIATED PRESS, September 12, 2008**)

Irvington, NJ
9/8/2008
A gunman shot a woman, her two teenage daughters, and a one-year-old girl before setting their house on fire. A woman and one-year old boy escaped the attack. (*Suspect in 4 N.J. slayings surrenders to police,* **ASSOCIATED PRESS, September 13, 2008**)

Tulsa, OK
9/7/2008
A drive by shooting with an SKS assault rifle left four teenagers wounded and one dead after their car was mistaken for a vehicle belonging to gang members. Three shooters were convicted. (Bill Braun, *Tulsa man convicted of murder, assaults in drive-by shooting case*, **TULSA WORLD, January 22, 2008**)

Tulsa, OK
9/7/2008
A 19 year old with an assault rifle opened fire on a group of teenagers. One teenager was killed and three others were seriously wounded. The shooter followed the victims for several blocks before the shooting. The victims reportedly knew the shooter although they were not considered friends of each other. (*Arrest made in deadly drive-by*, **TULSA WORLD, September 12, 2008**)

Visilia, CA
9/4/2008
Four men standing outside a home were shot. (*Four Visalia men injured in midnight shooting,* **VISALIA TIMES, September 4, 2008**)

Alger, WA
9/2/2008
A man shot eight people, six fatally, including a Sheriff's Deputy, and stabbed two others. The shooter had been released from jail a month earlier, had a protective order issued against him in the past, and was seriously mentally ill. (*6 dead, 2 wounded in Wash. State shooting spree,* **ASSOCIATED PRESS, September 2, 2008**)

Oakland, CA
9/1/2008

One person died and four were injured in a shooting. (*One dead, dour injured in Oakland shooting,* **OAKLAND TRIBUNE, September 1, 2008**)

Camden, NJ
9/1/2008
A man shot and killed his wife, mother-in-law, daughter, and son before killing himself. His seven year old granddaughter and ten yea old grandson managed to escape from the house. (Geoff Mulvihill, *Officials: Man kills 4 and himself in Camden NJ,* **ASSOCIATED PRESS, September 1, 2008**)

Las Vegas, NV
8/31/2008
Someone shot six people when an argument erupted at a party. A 20 year old man died and five other people were injured. (*Shooting Kills 20-year-old Man, Wounds 5 Others,* **KOLO-TV NORTHERN NEVADA, September 1, 2008**)

Columbus, NE
8/19/2008
Five people were wounded in a drive-by shooting. (*Teen Arrested in Columbus Shooting,* **WOWT-TV OMAHA, August 20, 2008**)

Chicago, IL
8/13/2008
Four people were injured when an attempted robbery turned into a shootout between the storeowner and two thieves. The two thieves, a store employee, and a bystander were all injured. (Steve Schmadeke, *Jewelery shop robbery attempt leaves 4 shot,* **CHICAGO TRIBUNE, August 14, 2008**)

Maywood, IL
8/9/2008
Four teenagers were shot, three fatally, as they were sitting in a parked car. (Monifa Thomas, *Three 18-year-old males killed in Maywood shooting,* **CHICAGO SUN-TIMES, August 10, 2008**)

Baton Rouge, LA
8/7/2008
One man died and three others were wounded in a drive-by shooting. (*4 shot, 1 fatally in drive-by,* **BATON ROUGE ADVOCATE, August 8, 2008**)

Roxbury, MA
8/5/2008
Four people were shot on the street. (Milton Valencia and John Guilfoil, *Four teens shot sitting on front steps,* **BOSTON GLOBE, August 6, 2008**)

Niagara, WI
7/31/2008
A man with an assault rifle massacred a group of teenagers, killing three and injuring a fourth. The group was gathered along a river to go swimming when the gunman emerged from surrounding woods and began shooting. (*Niagara, Wisconsin shooting suspect caught*, **CHICAGO TRIBUNE, August 1, 2008**)

Kansas City, MO
7/31/2008
A 22-year-old died and three others were injured when a gunman in a car opened fire into a crowd of people. (Christine Vendel, *Drive-by shooting kills one, followed by house shooting,* **KANSAS CITY STAR, August 1, 2008**)

McKees Rocks, PA
7/27/2008
A man shot four people in a bar. (Gabrielle Banks, *Warrant issued in shooting of 4 in McKees Rocks,* **PITTSBURGH POST-GAZETTE, July 28, 2008**)

Knoxville, TN
7/27/2008
A man carrying a shotgun entered the Tennessee Valley Unitarian Universalist Church and opened fire. Nine people were injured, two fatally. (Elizabeth Ryan, *Chattanooga: Knoxville shooting brings shock tears to local church,* **ASSOCIATED PRESS, July 28, 2008**)

Washington, DC
7/25/2008
Six people were shot on the street. (Clarence Williams, *Six people shot newar North Capitol Street,* **WASHINGTON POST, July 26, 2008**)

Bennett, CO
7/24/2008
A man shot and killed his wife, his three year old daughter, and another woman before killing himself. The man had escaped from prison several days before and was running from police with his family. (Kieran Nicholson, Howard Pankratz, Carlos Illescas, *Escapee kills family, self,* **DENVER POST, July 25, 2008**)

Corpus Christi, TX
7/18/2008
One man died and four others, including three children, were injured when several men shot at them in a residential area. (*Man killed in afternoon shooting identified,* **CORPUS CHRISTI CALLER TIMES, July 25, 2008**)

Atlanta, GA
7/17/2008
Two people were shot inside an apartment, and three more were shot outside the building. One person died. (Mike Morris, *One dead, four wounded in shooting,* **ATLANTA JOURNAL-CONSTITUTION, July 18, 2008**)

Saginaw, MI
7/16/2008
Five people were injured in a drive-by shooting. (Marc Jacobson, *Dangerous Night in Saginaw and Flint,* **WJRT-TV MID-MICHIGAN, July 16, 2008**)

Grandview, MO
7/14/2008

Four teenagers were shot while gathered in a diner parking lot. More than 40 rounds were fired at them. (Kevin Hoffmann, *Two still hospitalized from Grandview shooting,* **KANSAS CITY STAR, July 14, 2008**)

Detroit, MI
7/13/2008
Two men shot a woman and three children as they were standing on their porch. (Tom Grrenwood, *3 children and woman wounded in Detroit shooting,* **DETROIT NEWS, July 15, 2008**)

Cocoa, FL
7/7/2008
Four people were wounded and a nearby house was sprayed with bullets during a drive-by shooting. (*Five Shot in Revenge Drive-by, Police Say,* **WKMG-TV ORLANDO, July 7, 2008**)

Brooklyn, NY
7/6/2008
One woman died and four others were wounded when a shootout erupted at a barbecue. (Jamie Roth, *Family murns loss after Brookyn shooting,* **WABC-TV NEW YORK, July 7, 2008**)

Pompano Beach, FL
7/6/2008
An argument over fireworks at a street party lead to a gunman shooting into the crowd of party-goers, injuring three people and killing one. (*1 dead, 3 injured during shooting in Borward,* **ASSOCIATED PRESS, July 7, 2008**)

Milwaukee, Wi
7/4/2008
A gunman opened fire at a large street party, spraying more than 30 bullets into the crowd. He killed four people and wounded two others. (Carrie Antlfinger, *4 people dead in Milwaukee shooting,* **ASSOCIATED PRESS, July 4, 2008**)

Tracy, CA
6/30/2008
Five people were injured when a gunman fired into a group of people standing outside a house. (*Gang Shooting in Tracy Injures 5,* **KXTV-TV SACRAMENTO, July 1, 2008**)

Raleigh, NC
6/28/2008
Two men shot four people, two fatally. (Beau Minnick, *Two killed in Raleigh shooting,* **WRAL-TV RALEIGH, June 29, 2008**)

Henderson, KY
6/24/2008
A disgruntled employee shot his boss and five other workers at a plastics plant before killing himself. One worker survived the massacre. (Harry Weber, Bruce Schreiner, Rebecca Yonker, *6 Dead in Henderson, Ky., Plastics Plant Shooting,* **ASSOCIATED PRESS, June 25, 2008**)

Chicago, IL
6/23/2008

Four people were injured and a 17-year-old killed in a drive-by shooting. (*Crime Watch,* **CHICAGO TRIBUNE, June 25, 2008**)

Corinth, MS
6/23/2008
Four people were shot at a party. (**source**)

Anderson, NC
6/22/2008
Three men with an AK-47 assault rifle fired on a group of teenagers standing outside an apartment building. Three of the teens were injured and another was killed. (Jane Hindmon, *Police: AK-47 Used In Anderson Shooting*, **WHNS-TV 21 GREENSVILLE, June 25, 2008**)

St Paul, MN
6/22/2008
One man was killed and three others injured when they were shot outside of a nightclub. (Pat Pheifer, *St. Paul club where father of 5 was killed has history of violence,* **STAR TRIBUNE, June 23, 2008**)

Miami, FL
6/13/2008
A man shot six people at a graduation party with an assault rifle. One of the victims died. (*Teen shot and killed while leaving graduation party*, **WSVN - TV 7 MIAMI, June 13, 2008**)

Carrollwood, FL
6/7/2008
A man shot and killed his estranged wife, and two others at her house, including the local fire captain. The gunman then got in a shootout with police, wounding two officers, before he was killed. (Mitch Stacy, *Four killed in related shootings,* **SARASOTA HERALD-TRIBUNE, June 9, 2008**)

Philadelphia, PA
6/7/2008
Two men fired into a crowd of people, injuring four people, including a six year old girl. (*3 adults, 1 child shot outside philadelphia club,* **ASSOCIATED PRESS, June 8, 2008**)

Mountain Grove, MO
6/5/2008
A man shot his estranged wife and two of her relatives before shooting himself. The man and the two relatives all died. (Kate Stacy, *Four Die in Mtn. Grove Murder-Suicide,* **Ozarks First, June 6, 2008**)

Ingram, TX
6/1/2008
A man shot and killed his six and nine year old sons, his wife, and his mother-in-law before killing himself. The shooter had been arrested previously for domestic violence. (Zeke MacCormack, *Ingram family of 5 killed; murder-suicide suspected,* **SAN ANTONIO EXPRESS-NEWS, June 2, 2008**)

North Versailles, PA

6/1/2008
A man shot five people in a bar after arguing with another customer. (*North Versailles Shooting Leaves Five Injured, Including Former Steeler,* **WPXI-TV PITTSBURGH, June 2, 2008**)

Tulsa, OK
5/31/2008
Six teenagers were injured in a drive-by shooting. (*OKC police chief wants tighter gun laws,* **ASSOCIATED PRESS, June 2, 2008**)

Jacksonville, FL
5/29/2008
Five people were injured in a drive-by shooting. (*Police: 5 People Injured in Drive-by Shooting,* **WJXT-TV JACKSONVILLE, May 30, 2008**)

Jackson, MS
5/26/2008
Five people were shot, one fatally, at a Memorial Day barbecue. A man had left the party after arguing with others in attendance only to return with an assault rifle and fire indiscriminately into the crowd. (David Kenney, *Memorial Party Shooting Arrest*, **WLBT - TV 3 JACKSON, May 27, 2008**)

Fort Worth, TX
5/25/2008
Five teenagers were shot when a fight broke out at a party. (*5 shot I what police say was gang incident,* **FORT WORTH STAR-TELEGRAM, May 26, 2008**)

Winnemucca, NV
5/25/2008
A man entered a bar and shot four people, two fatally. There was a history of disagreements between the families of the shooter and the victims. (*Winnemucca Authorities Suspect Revenge Spurred Bar Shootings,* **ASSOCIATED PRESS, May 28, 2008**)

Arleta, CA
5/20/2008
Four men were shot in a drive-by shooting. (Brandon Lowry, Aron Miller, *Night of Valley gun violence kills two, wounds eight,* **Los Angeles Daily News, May 21, 2008**)

Baltimore, MD
5/15/2008
Three people were injured and one killed after being shot in a residential neighborhood. (*One Dead in Wake of Quadruple Shooting,* **WBAL-TV BALTIMORE, May 16, 2008**)

Riverdale, IL
5/13/2008
Five people were shot at a bar after a fight erupted. (*Five injured in suburban Chicago bar shooting,* **ASSOCIATED PRESS, May 14, 2008**)

Saucier, MS
5/6/2008

A man killed his two teenage stepdaughters and one of the girl's boyfriends before killing himself in a domestic dispute. (*Four Dead in Saucier Shooting,* **Associated Press, May 6, 2008**)

Stafford, VA
5/5/2008
A man shot and killed his girlfriend with an AK-47 assault rifle before killing his one-year-old son, two-year-old daughter, and himself with a handgun in their home. (Martin Weil, Elissa Silverman, *4 Fatally Shot in Trailer Park,* **Washington Post, May 6, 2008**)

Big Spring, TX
5/4/2008
A 20 year old pregnant woman and 21 year old man died, and four others were injured, including a three year old girl, in a drive-by shooting. (*Family killed in drive-by shooting in Big Spring,* **Victoria Advocate, May 6, 2008**)

Phoenix, AZ
5/3/2008
Two teens died and five more were wounded when a fight erupted at a party and rival gang members started shooting at each other. (JJ Hensley, *2 dead, 5 wounded in gang-related shooting,* **Arizona Republic, May 4, 2008**)

Torrance, CA
4/28/2008
A man shot his wife, her mother, their five-year-old son, and then himself hours after his wife took out a restraining order against him. Only the wife survived the shooting. (*Police: 3 dead, 1 wounded in murder-suicide,* **Associated Press, April 29, 2008**)

Los Angeles, CA
4/28/2008
Five people were shot, two fatally, in a drive-by shooting. The two who died were in a parked car, and the three who were injured were sitting on a porch nearby. (Francisco Vara-Orta, *Los Angeles; 2 dead, 3 injured in shooting,* **Los Angeles Times, April 29, 2008**)

Easley, SC
4/26/2008
An 18-year-old shot and killed his father, brother, stepmother, and stepsister at their house. He had broken up with his girlfriend and moved back home two weeks earlier. (*Teen Xharged with Killing Four Family Members,* **Associated Press, April 28, 2008**)

Chicago, IL
4/23/2008
Five people were shot to death in a house that was also ransacked. The perpetrators have not been identified. (Sara Olin, Antonio Olivo, Angela Rozas, *5 found slain in South Side home,* **Chicago Tribune, April 24, 2008**)

Jacksonville, FL
4/18/2008
Four men were shot at a nightclub after getting into a dispute with the shooter earlier that evening. *Four Men Shot Outside Club,* **Associated Press, April 18, 2008**)

Peoria, AZ
4/15/2008
Three people were killed and another injured after being shot in their home. (Cecilia Chan, *3 slain, 1 wounded in Peoria shooting incident*, **Arizona Republic, April 15, 2008**)

Fort Worth, TX
4/6/2008
A five-year-old girl and her grandmother died, and three other children and a woman were injured when they were shot while at a child's birthday party at an appartment. A gunman fired many rounds at the children who were playing outside. (Deanna Boyd, *Man held in Fort Worth double-slaying,* **Fort Worth Star-Telegram, April 8, 2008**)

Kansas City, MO
3/31/2008
A 22 year old died and three others were injured when a gunman in a car opened fire into a crowd of people. (**source**)

Richmond, CA
3/30/2008
A man fired multiple times into a crowd of people who were setting up for an event outside a church. One person died and four others were seriously injured. (*One killed, four injured in shooting outside Richmond church,* **Associated Press, March 31, 2008**)

Hyattsville, MD
3/26/2008
Four assailants robbed and shot four men, killing two of them. (Elissa Silverman, CLarence Williams, *2 Men Killed, 2 Hurt in Hyattsville Shooting,* **Washington Post, March 27, 2008**)

Santa Maria, CA
3/20/2008
A man with a semi-automatic handgun shot his father, two employees, and a customer at his father's junkyard. All four victims died. (*4 dead in shooting at auto wrecking yard,* **Associated Press, March 20, 2008**)

Virginia Beach, VA
3/19/2008
A man shot five people, killing two, with an AK-47 assault rifle and .9 mm handgun before killing himself. The man was about to be evicted from his apartment and targeted the apartment complex's employees in his attack. (*Gunman in mass shooting identified*, **WVEC - TV 13 Hampton Roads, VA, March 20, 2008**)

West Palm Beach, FL
3/4/2008
A man walked into a Wendy's restaurant at lunchtime and shot six people before killing himself. One of the victims died. (*Motive Still Mystery after West Palm Beach Wendy's Shooting,* **WPBF-TV West Palm Beach, March 4, 2008**)

Memphis, TN
3/2/2008

A man shot and killed his brother after the two argued, then tried to kill everyone else who was in the house. The man shot and killed three other adults with a semi-automatic handgun, and beat and stabbed four children, killing two of them. In all, six people died and two were injured. (*Victims of Memphis shooting had extensive criminal records…,* **Associated Press, March 8, 2008**)

Emory, TX
3/1/2008
A teenage girl, along with her boyfriend and two other friends, shot and stabbed each member of her family before setting their house on fire. Her mother and two younger brothers died; her father survived despite being shot five times. The girl was unhappy because her parents did not like her boyfriend and were trying to break the two up. (*Police: Boyfriend wanted to kill girl's parents,* **Associated Press, March 3, 2008**)

Los Angeles, CA
2/28/2008
Five children and three adults were injured when a man shot into crowd of people waiting at a bus stop. A nearby school had just let out for the day. (*5 kids among 8 injured in L.A. shooting,* **Associated Press, February 28, 2008**)

Bristol, TN
2/27/2008
A man angry about a breakup with an ex-girlfriend killed her mother, current boyfriend, friend, and neighbor at her appartment. He fled the scene but later killed himself as police closed in on his location. (*Shooting in Downtown Bristol Kills 3, Injures 1,* **Associated Press, February 27, 2008**)

Baldwin Park, CA
2/25/2008
A man with a history of serious mental illness fatally shot his mother with a handgun and then went to his neighbor's home, where he killed a woman and a four-year-old girl, and wounded two other children. (*California Man Charged with Shooting 2 Women, 3 Children,* **Associated Press, February 26, 2008**)

Yorba Linda, CA
2/24/2008
A man shot and killed his wife and three small children, and wounded his stepson before killing himself with a shotgun. (*5 dead, 1 hurt in California shooting,* **Associated Press, February 25, 2008**)

DeKalb, IL
2/14/2008
A man entered a lecture hall at Northern Illinois University and began shooting. He killed five students and wounded 16 before killing himself. (*Gunman Was Once 'Revered' on Campus*, **New York Times, February 15, 2008**)

Canton, OH
2/10/2008
Four people were shot at a bar. (**source**)

Orlando, FL
2/10/2008
Five high school students were injured when someone fired into a crowd at a party. (*5 Shot at Sweet 16 Party,* **WESH-TV Orlando, February 10, 2008**)

Mount Olive, SC
2/9/2008
Four people were injured after an argument between two families erupted into gunfire. (**source**)

Los Angeles, CA
2/7/2008
A man killed three relatives and then entered into a lengthy standoff when police arrived at his house. In the standoff, the gunman killed one SWAT officer and injured another before he was killed. (*Gunman in Los Angeles Shooting Had History of Mental Illness, Police Say,* **Associated Press, February 8, 2008**)

Kirkwood, MO
2/7/2008
A man entered a Kirkwood City Council Meeting and started shooting city officials. In all, the gunman killed six people and injured at least two others. Police later killed the gunman. (*Six dead, two wounded in shooting at Kirkwood council meeting,* **KWMU – St. Louis Public Radio, February 8, 2008**)

Utica, NY
2/3/2008
Five guests were injured when shots were fired at a crowded house party. (Rocco LaDuca, *Police to crack down on late night-night parties after 5 people are shot,* **Utica Observer-Dispatch, February 4, 2008**)

Tinley Park, IL
2/2/2008
Six women were tied-up and then shot at a suburban clothing store. Five of the women died. The gunman escaped and has not been identified. (John Gress, *Five killed in Chicago-area store shooting,* **Reuters, February 2, 2008**)

Cockeysville, MD
2/1/2008
A 15-year-old boy shot his parents and two younger brothers while they were sleeping. (Kasey Jones *Md. Boy Admits Killing Parents, Brothers*, **Associated Press, February 3, 2008**)

Washington, DC
1/22/2008
Four Ballou High School students were shot as they were leaving school at the end of the day. All students are expected to recover. (*3 shot shortly after high school dismissal*, **Associated Press, January 22, 2008**)

Indianapolis, IN
1/14/2008

Two intruders demanding money and drugs repeatedly shot two women and their two young children. All four victims died. (Emily Udell, *2 women, 2 young children killed in Indy shooting,* **ASSOCIATED PRESS, January 15, 2008**)

Washington, DC
1/11/2008
A 17-year-old shot five men between the ages of 17 and 20 from his car. (**source**)

Walnut Hills, OH
1/7/2008
A gunfight in a bar leaves four injured. (*Gunfight in Walnut Hills Injures 3 Bystanders,* **WLWT-TV CINCINNATI, January 8, 2008**)

Long Beach, FL
1/1/2008
Four people were injured when someone shot at a group of partygoers in a Long Beach backyard. (*New Year shooting stuns family,* **LONG BEACH PRESS-TELEGRAM, January 2, 2008**)

Carnation, WA
12/24/2007
A woman and her boyfriend, using large-caliber pistols, shot and killed six members of her family, including her parents, her brother and wife, and their two children, ages three and six. The shootings took place in and around the parents' house on Christmas Eve. (Scott Gutierrez, *Carnation suspects tell officers of victims' frantic, final moments,* **Seattle POST-INTELLIGENCER, December 28, 2007**)

Las Vegas, NV
12/11/2007
Two assailants using 9-millimeter and .45-caliber guns shot six people as they exited a Mojave High School bus. The attack followed a fight at school earlier in the day. (*6 People Shot After Exiting a School Bus in Las Vegas*, **ASSOCIATED PRESS, December 12, 2007**)

Arvada & Colorado Springs, CO
12/9/2007
One man with an assault rifle attacked a missionary training center in Arvada and a church in Colorado Springs. He killed two people and injured two others in Arvada, and killed two and injured three others in Colorado Springs. He died after being shot by a security guard and then shooting himself. (Erin Emery, *Report details church shooting, the document chronicles the days leading up to the Dec. 9 deaths of four young people,* **DENVER POST, March 13, 2008**)

Omaha, NE
12/5/2007
Nine people were shot to death and five others were injured after a 20 year old shooter, armed with a military-style assault rifle, attacked shoppers in a department store in a Nebraska mall. (*The American Way*, **EUGENE REGISTER-GUARD, December 17, 2008**)

Jackson, MO
12/3/2007

A man fatally shot his wife, four-year-old daughter, and 16-year-old stepson before killing himself with .38 caliber handgun in their home. He also shot his 2-year-old daughter. (*Third Victim Dies After Jackson Shooting*, **HEARTLAND NEWS, December 3, 2007**)

Laytonsville, MD
11/22/2007
A man shot and killed ex-wife and their three children at a park before killing himself with a 22-caliber rifle. The victims were each shot multiple times. (*Five dead, including 3 children, in Md. Murder-suicide*, **ASSOCIATED PRESS, November 23, 2009**)

Denver, CO
11/4/2007
A man shot into a crowd of people exiting from a nightclub at closing time, leaving six people injured and one dead. There had been an incident in the bar earlier that night. (*Group shot outside Denver nightclub; 1 killed, 7 wounded,* **ASSOCIATED PRESS, November 4, 2007**)

Federal Heights, CO
10/31/2007
Five people were injured during Halloween party when two men who were arguing began shooting at each other. (Kirk Mitchell, Kieran Nicholson *At least 5 shot at wild Adco party*, **DENVER POST, November 2, 2007**)

Saginaw, MI
10/25/2007
Two Arthur Hill High School students and two adults were shot by another student as they left a middle school football game. *Charges sought in shooting at Saginaw school football game*, **ASSOCIATED PRESS, October 25, 2007**)

Detroit, MI
10/18/2007
Two adults and two children, 5 and 9 years old, were shot and killed as they were sleeping. (*Adults, Children Found Shot Dead in Detroit Home,* **Associated Press, October 18, 2007**)

Cleveland, OH
10/10/2007
A 14 year old student shot two teachers and two students at SuccessTech Academy before killing himself. He had been suspended for fighting earlier in the week and had threatened to harm other students and blow up the school previously. (*Student, 14, Shoots 4 and Kills Himself in Cleveland School*, **NEW YORK TIMES, October, 10, 2007**)

Crandon, WI
10/7/2007
An off-duty Sheriff's deputy killed six and wounded a seventh person when he burst into a pizza party and started shooting with an AR-15 Assault Rifle. The shooter later killed himself as the police closed in. (Todd Richmond, *Crandon mass murder-suicide: Questions linger in killing of seven, officials tight-lipped despite suspect's death*, **ST. PAUL PIONEER PRESS, December 4, 2007**)

Camden, NJ
9/21/2007

A man shot four people, including a three-year-old and a 73-year-old, on a Camden street. (Leo Strupczewski, *Suspect sought in Camden shootout,* **CHERRY HILL COURIER POST, September 23, 2007**)

Detroit, MI
9/17/2007
Five people were shot, four fatally, in a house. (*Four killed in Detroit home shooting*, **UPI, September 18, 2007**)

New Orleans, LA
9/15/2007
At least 28 bullets were fired from an AK-47 at an outdoor birthday party for 5 year old twins in the courtyard of a public housing complex. A 19 year old was killed and three children were wounded, ages 7, 8 and 13. (Mary Sparacello, *Housing Authority reining in parties, Kenner shooting leads to regulations*, **NEW ORLEANS TIMES PICAYUNE, October 11, 2007**)

Miami, FL
9/13/2007
Police spotted a vehicle driving erratically and followed it until it stopped in a residential complex. The driver got out and hopped a fence to the rear of the home; the officers exited their patrol car and went to the front of the home where they were granted permission to search by a female resident. The suspect grabbed a high-powered, military-grade rifle and fired at the police officers through a window, killing one officer, then exited the house and shot three other officers as he escaped. The shooter was caught later that day but would not relinquish his assault rifle so he was shot and killed by police officers. (David Ovalle et. al., *The murder and the manhunt started in a South Miami-Dade townhouse, zigzagged…*, **MIAMI HERALD, September 13, 2007**)

Dallas, TX
8/12/2007
One person was killed and three others wounded in a shooting outside a poetry/coffee shop. The gunman, who used an assault rifle, fled the scene. (Marissa Alanis, *Peacekeeper is killed outside club, police say: Dallas 3 others injured as gunman fires assault rifle into crowd*, **DALLAS MORNING NEWS, August 13, 2007**)

Newark, NJ
8/4/2007
Three Delaware State University students were shot and killed execution style by a 28 year old and two 15 year old boys. The three friends were forced to kneel against a wall behind an elementary school and were shot in the head. A fourth student was found about 30 feet away with gunshot and knife wounds to her head. (*Third Suspect Arrested in Student Killings*, **ASSOCIATED PRESS, August 11, 2007**)

Atlanta, GA
7/23/2007
A man neighbors described as a retired factory worker shot three people to death and wounded two others before killing himself in a southwest Atlanta home. (*Atlanta Shooting Leaves 4 Dead, Including Gunman, 2 Injured,* **ASSOCIATED PRESS, July 23, 2007**)

Philadelphia, PA

7/22/2007
Police said a man opened fire shortly after midnight at Abay Wheelers Bar in the Kingsessing section of the city, killing three men, ages 20, 30 and 31, and injuring a fourth. (Dan Cuellar, *Weekend Violence Leaves 6 Dead,* **WPVI-TV PHILADELPHIA, July 22, 2007**)

Indianapolis, IN
7/16/2007
A man with a history of mental illness plead guilty to wounding four co-workers in a shooting at a factory. He waited for his co-workers to show up for work, and then began to fire. (Jon Murray, *Co-worker pleads guilty in shootings,* **INDIANAPOLIS STAR, July 17, 2007**)

Cleveland, OH
7/4/2007
Enraged by the fireworks being set off at a neighbor's party, Terrence Hough, Jr., 35, confronted them with a handgun. Hough opened fire on the guests, killing three and wounding two others. According to his co-workers at the Cleveland fire department, Hough was a "ticking time bomb" with a prior DUI conviction, yet he reportedly managed to qualify for a CCW license in 2004. Police seized twelve other firearms from Hough's home. (Mike Tobin, *Hough known for bursts of anger*, **CLEVELAND PLAIN DEALER, July 7, 2007**)

Moscow, ID
5/20/2007
A card-carrying member of the Aryan Nation with a lengthy criminal record, Jason Kenneth Hamilton, 36, went on a killing spree with a semi-automatic military rifle. Hamilton shot his wife in their home before opening fire on the Latah County courthouse, killing one police officer and wounding three others. Hamilton then took refuge in a nearby church, killing a church sexton before turning the gun on himself. Hamilton had a long criminal history across four states, including arrests for violent crimes, domestic violence, drugs, and weapons offenses, yet he was licensed to own fully automatic weapons and to carry concealed weapons. (Taryn Brodwater et al., *Shooter linked to Aryans*, **IDAHO SPOKESMAN REVIEW, May 23, 2007**)

Blacksburg, VA
5/16/ 2007
Thirty-three people were killed Monday on the campus of Virginia Tech in what appears to be the deadliest shooting rampage in American history. Many of the victims were students shot in a dorm and a classroom building. Witnesses described scenes of mass chaos and unimaginable horror as some students were lined up against a wall and shot. Others jumped out of windows to escape, or crouched on floors to take cover. (Virginia Tech Shooting Leaves 33 Dead, **New York Times, April 16 2007**)

Kansas City, MO
3/5/2007
One man was killed and three injured during a drive-by shooting of a tire store. The shooters used two .223-caliber assault rifles, one of which had two large drum magazines and could fire 100 bullets without reloading. Police pursued the shooters, who were eventually apprehended, and were shot at with the same assault rifles. The following day, three retaliatory shootings occurred; the day after, one retaliatory shooting occurred in which a woman was shot seven times in the chest and torso. (Christine Vendel, *Heavy firepower in KC: Officers outgunned by suspects*, **KANSAS CITY STAR, March 8, 2007**)

Philadelphia, PA
2/13/2007
A gunman used an assault weapon to kill three and wound another before killing himself. (Larry King & Joseph A. Gambardello, *Investor rage, lethal trap*, **PHILADELPHIA INQUIRER, February 14, 2007**)

Salt Lake City, UT
2/12/2007
A teen opened fire inside the Trolley Square Mall, killing five, before being shot and killed by police. (*Gunman Kills 5 in Shooting Spree at Salt Lake City Mall Before Being Killed by Police,* **ASSOCIATED PRESS, February 13, 2008**)

New Bedford, MA
12/12/2006
Three people were killed and two police officers were injured when a gunman opened fire a strip club; the shooter was fatally shot. One of the weapons used was described as an AR-15. (Jessica Heslam, *Strip club gunman at 'crossroads', killer bid farewell in cell phone messages*, **BOSTON HERALD, December 14, 2006**)

Chicago, IL
12/9/2006
A disgruntled client of attorney Michael McKenna forces his way into a busy downtown office building, where he barricades himself inside, shoots and kills McKenna and two other employees. The shooter was killed after a gunfight with the SWAT team. (*Four Men, Including Gunman, Killed in Chicago Skyscraper Shooting,* **ASSOCIATED PRESS, December 9, 2006**)

Newport, KY
11/19/2006
A fight at a nightclub led to four people being shot that evening. A 23 year old was shot several times and left for dead on a bridge. An hour later, police found a 20 year old man shot dead in his vehicle. Two other people were taken to the hospital with gunshot wounds and police recovered casings from an assault weapon. (*A fight at a Northern Kentucky nightclub lead to a wild shooting spree*, **WLEX - TV 18 LEXINGTON, KY, November 19, 2006**)

Nickel Mines, PA
10/2/2006
A dairy truck driver walked into a one-room Amish schoolhouse with a shotgun, a semi-automatic handgun, and 600 rounds of ammunition, selected all the female students, and shot them execution-style, killing five and seriously wounding six. The man then shot himself, apparently having left suicide notes beforehand. (*Man Shoots 11, Killing 5 Girls, in Amish School*, **NEW YORK TIMES, October 2, 2006**)

Pittsburgh, PA
9/17/2006
Five Duquesne University basketball players are wounded, one critically, after a shooting on campus following a dance, the first such incident in the 128-year history of the University. (*2nd Arrest Is Made in Duquesne Attack*, **NEW YORK TIMES, September 21, 2006**)

Essex, VT
8/24/2006

A gunman shot five people, killing two of them, in a rampage through two houses and an elementary school, before wounding himself. (*Deadly Rampage in Quiet Vt. Town; Man Kills Two, Hurts Two Others, in Shootings at School, Homes*, **Boston Globe, August 25, 2006**)

Seattle, WA
7/28/2006
A man forced his way into the offices of the Jewish Federation of Greater Seattle by putting a gun at the back of a 13-year-old girl to gain entry to the building. The shooter carried two semi-automatic handguns and extra ammunition, shooting six and killing one. (*1 Killed, 5 Wounded in Seattle Jewish Center Shooting,* **Associated Press, July 29, 2006**)

Miami, FL
6/6/2006
Three men were killed and another injured when the van they were riding in was shot numerous times by assault weapons. About 50 rounds were fired into the van. (David Ovalle, *Ambush takes lives of 3 men*, **Miami Herald, June 6, 2006**)

Indianapolis, IN
6/2/2006
Seven family members, four adults and three children, were shot and killed in their home by a robber armed with an assault rifle. Nearly 30 shell casings were found. (Ashley M. Heher, *Suspect in slaying of 7 family members surrenders / Indianapolis police say he had nowhere else to go*, **Houston Chronicle, June 4, 2006**)

Fort Worth, TX
5/9/2006
A 16 year old innocent bystander was shot and killed as he stood outside a convenience store when gang members drove up and fired on other gang members in the vicinity. Five minutes later, one man was shot in the leg and another in the foot during a second drive-by shooting. One hour and a half later, a 50 year old woman was shot in the shoulder by gunfire from a high-powered assault weapon as she stood in her kitchen. (Deanna Boyd, *Teen killed in shooting at convenience store*, **Fort Worth Star-Telegram, May 9, 2006**)

Seattle, WA
3/25/2006
Kyle Huff walked into a party and killed six people in the Capitol Hill neighborhood before killing himself. A 12-gauge Winchester pump shotgun with a pistol-grip and a .40-caliber semiautomatic Ruger handgun were used in the shootings. (*Seven people dead in Capitol Hill shooting,* **Seattle Times, March 25, 2006**)

Tacoma, WA
11/20/2005
A 20 year old male opened fire in a Tacoma mall, wounding six. The shooter took four hostages, all of whom were released unharmed. (*Suspect: 'follow screams', Man opens fire at mall in Tacoma; 6 wounded*, **Akron Beacon Journal, November 22, 2005**)

New Orleans, LA
9/4/2005

Five New Orleans officers were charged with shooting to death two unarmed civilians and wounding four others near the Danziger Bridge seven days after Hurricane Katrina. Five other officers pleaded guilty in the incident.
(*FBI agent tells of Katrina shootings police cover-up.* **WHTC, July 19, 2011.)**

Clayton County, GA
4/23/2005
A high school senior was killed and three others wounded when a teenage assailant fired an AK-47assault rifle into a crowd of partygoers exiting a home. (*Teen faces murder charge*, **ATLANTA JOURNAL-CONSTITUTION, May 28, 2005**)

Red Lake Indian Reservation, MN
3/21/2005
At the time, the worst school-related shooting incident since the Columbine shootings in April of 1999. Ten killed and seven injured in rampage by high school student. (*A Very Quiet Sense of Shock'; Small Community Struggles to Cope*, **WASHINGTON POST, March 23, 2005**)

Brookfield, WI
3/14/2005
A madman fires 22 rounds during a church service. Seven are killed, including the minister and his son, and four others are wounded. (*Police Believe Shooting Motive May Be Related to Church, Service,* **WISN-TV MILWAUKEE, March 15, 2005**)

Atlanta, GA
3/11/2005
Gunman opens fire in a courtoom killing a judge, court reporter, police officer, and bystander. He flees the scene and is suspected of killing a customs officer before he is apprehended. (Shaila Dewan, *Atlanta shooting spree suspect arrested,* **NEW YORK TIMES, March 14, 2005**)

Tyler, TX
2/25/2005
A gunman with a history of domestic violence and a felony conviction, who was reportedly fighting with his ex-wife over child support for their two youngest children, shot over 50 rounds from an SKS assault rifle on the steps of his local courthouse when his ex-wife exited the building. His ex-wife was killed along with a bystander who tried to shoot the gunman. The shooter's 23 year old son and three law enforcement officers were wounded during the shooting, including a 28 year old deputy who was in grave condition. The gunman fled the scene but was pursued and shot by police when he exited his car and shot toward officers. (Bill Hanna & Jack Douglas Jr., *Rampage in Tyler leaves three dead, four wounded*, **FORT WORTH STAR-TELEGRAM, February 25, 2005**. Jack Douglas Jr. & Bill Hanna, *Police order emergency trace on weapon used in shootings* **FORT WORTH STAR-TELEGRAM, February 26, 2005**)

This list is a compilation of mass shootings found in available news outlets. It is not comprehensive.
