# Supplementary material for: Contagion in Mass Killings and School Shootings
Source: PLoS One. 2015 Jul 2;10(7):e0117259. doi: 10.1371/journal.pone.0117259 (PMC4489652; doi:10.1371/journal.pone.0117259)
Supplement: S1 Data — (GZ) [file pone.0117259.s002.gz › data/brady_school_shootings_to_jan_2014.pdf]

Brady Campaign
To Prevent Gun Violence
SENSIBLE GUN LAWS SAVE LIVES

MAJOR SCHOOL SHOOTINGS IN THE UNITED STATES SINCE 1997

Rosewell, New Mexico
1/14/2014
A 12-year old middle school student opened fire in his gym with a shotgun and critically wounded two students in Roswell, N.M., according to authorities.The injured were a 13-year-old girl and a 12-year-old boy. The shooting occurred shortly before class was to begin at Berrendo Middle School. (ABC News)

Centennial, Colorado
12/13/2013
Karl Pierson, an 18-year old student, walked into Arapahoe High School and opened fire, injuring one student before shooting and killing himself. He bought the shotgun legally from a gun store in the community. (Associated Press)

Winter Garden, Florida
12/4/2013
A 15-year-old student is shot at West Orange High School has students were being dismissed for the day. Authorities identified the gunman as a 17-year-old fellow student. He's in custody facing charges of attempted murder, aggravated battery with a firearm, possession of a firearm by a minor and possession of a firearm on school property. (Orlando Sentinel)

Pittsburgh, Pennsylvania
11/13/2013
At least one gunman opens fire on three Brashear High School students as they walked to their cars after classes had ended for the day. One student was grazed in the head, another was struck in the neck and shoulder, and a third was hit in the leg and foot. Six people were taken into custody. Authorities believe the shooting was drug-related. (Wikipedia, List of School Shootings)

Greensboro, North Carolina
11/2/2013
A 21-year-old student is shot at North Carolina A&T State University. The victim was hospitalized with serious, but non-life-threatening injuries. The University locked down for half an hour. No suspects in custody.
(Wikipedia, List of School Shootings)

Sparks, Nevada
10/21/13
A twelve year old student shot and killed a teacher and wounded two other students before killing himself with a handgun on a playground basketball court at Sparks Middle School. (Wikipedia, List of School Shootings)

Austin, Texas
10/15/13
A seventeen year old student killed himself in front of his peers at Lanier High School. (Wikipedia, List of School Shootings)

Santa Monica, California
6/7/13
A lone gunman killed six, injured four in the library of Santa Monica College. The gunman was killed by police officers. The shooter killed his father and brother, leaving their bodies in a house which he had set on fire.
(Wikipedia, List of School Shootings)

Cambridge, Massachusetts
4/19/13
The suspects of the Boston bombing murdered a campus police officer at Massachusetts Institute of Technology. One of the suspects was killed in a gun fight with the police. (Wikipedia, List of School Shootings)

Orlando, FL
3/18/13
At the University of Central Florida, a gunman planned a columbine type killing. He pulled a fire alarm with the intention of gathering people in one place. Before James Oliver Seevakumaran killed anyone, he killed himself. Police found several loaded weapons and four homemade bombs in the possession of the deceased. (Wikipedia, List of School Shootings)
Atlanta, Georgia

1/31/13
A student shot a fellow student in the back of the neck at an Atlanta middle school. A teacher was injured as well because multiple shots were fired. A police officer wrestled the gun away before more damage was done. (Wikipedia, Nytimes)

Midland City, Alabama
1/29/13
A gunman boarded a school bus, killing the driver and kidnapping six children in what is known as the 2013 Alabama bunker hostage crisis. (Wikipedia, List of School Shootings)

Houston, Texas
1/22/13
At Lone Star College, two men argued, one pulled a gun and shot the other, injuring him. He also injured a nearby maintenance man and mistakenly shot himself in the leg.
(ABC News, also Wikipedia)

Chicago, Illinois
1/16/13
A 17 year old boy shot to death after a basketball game at Chicago State University. (Wikipedia, List of School Shootings)

Hazard, Kentucky
1/15/13
Three killed by Dalton Lee Stidham at Hazard Community and Technical College. The deaths include a twelve year old child who died the next day from the wounds. (Wikipedia, List of School Shootings)

St. Louis, MO
1/15/13
Two injured as a part time student at Stevens Institute of Business and Arts shot himself and a school administrator. (Wikipedia, List of School Shootings)

Detroit, Michigan
1/12/13

One sixteen year old boy shot and injured after a basketball game at Osborn High. (Wikipedia, List of School Shootings)

Taft, California
1/10/13
Two injured at the hands of a sixteen year old male student and a shot gun. The shooter, a student of Taft Union High, was convinced by his teacher to stop his shooting before more damage was done. (Wikipedia, List of School Shootings)

Newtown, CT
12/14/2012
26 people were shot and killed at Sandy Hook Elementary School. The gunman, a 20-year-old man, killed 20 young students and 6 staff at the school, a K-4th grade facility. (*26 Dead at Newtown School*, **NBC, December 14, 2012**).

San Bernardino, CA
12/8/2012
Campus police of California State University – San Bernardino shot and killed a man at an off-campus housing facility. A conflict that occurred in the dorm between the man, aged 38, and campus police led to the student's death and the injury of one officer.(*Cal State San Bernardino student shot, killed by campus police*, **ABC, December 8, 2012**)

Baltimore, MD
11/30/2012
One person was shot and injured at Baltimore-area Morgan State University, a school with an enrollment of about 6000 students. The victim was an MSU football player, and was the second person shot on the campus this semester. (*1 shot at Morgan State University*, **CBS News, November 30, 2012**)

Los Angeles, CA
10/31/2012
Four individuals who were not University of Southern California students were injured outside a Halloween party on the USC campus. The violence erupted while a group of more than 100 individuals waited in line. (*4 injured*

at USC campus shooting in Los Angeles, **Yahoo! News, November 2, 2012**)

Baltimore, MD
9/12/2012
One person was shot in the stomach on the campus of Morgan State University. The incident took place in the student center, yet neither the shooter nor the victim were MSU students. The two men were believed to be on campus for a sporting event.
(*Man shot at Morgan State University*, **NBC, September 13, 2012**).

Perry Hall, MD
8/27/2012
A 15-year-old Baltimore County high school student wounded one classmate on the first day of school in Perry Hall, Maryland. The shooting took place in the Perry Hall High School cafeteria in the morning. Teachers in the vicinity were able to rush the shooter before he could fire more shots.
(*Student shot on first day of classes at Maryland school*, **The Huffington Post, August 27, 2012**)

Auburn, AL.
6/11/2012
Three people are dead and three more are wounded in a shooting at a pool party near Auburn University. Two of the slain victims were former players for the school's powerhouse football program. (*Manhunt for suspect after 3 killed in shooting near Auburn University, including two former football players*, **Fox News, June 10, 2012**)

Oakland, CA
4/2/2012.
A 43-year-old former student at Oikos University, a Christian school populated by mostly Korean and Korean-Americans, opened fire on the campus, killing seven people and wounding several others. (*Oikos University Shooting: Suspect, One L. Goh, Detained; At Least 7 Dead*, **ABC News, April 2, 2012**)

Starkville, MS
3/24/2012

Mississippi State University student John Sanderson is shot to death in his dormitory room (Mississippi State University Student John Sanderson Shot to Death in Campus Dorm, *Three Suspects Still at Large*, **Hinterland Gazette, March 25, 2012**)

Jacksonville, FL
3/6/2012
Shane Schumerth, a 28-year-old teacher at Episcopal High School, returned to the campus after being fired and shot and killed the headmistress, Dale Regan, with an assault rifle. (*Digging Deeper: Shane Schumerth*, **Fox 30 Waws, 3/6/2012**)

Chardon, OH
2/27/2012
A 17 year old boy fired 10 shots at a group of students in the Chardon High School cafeteria as school began Monday, then shot an additional student elsewhere in the cafeteria, then proceeded down a hallway, where he shot one more student. He then fled and was arrested nearby, authorities said. Three boys were killed. Another male victim remains hospitalized, while a female victim has been released to her family, officials said. (*Third Teen Dead in School Attack,* **Wall Street Journal, February 29, 2012**)

Port Orchard, WA
2/23/2012
A 9 year old boy boy accidently shot a fellow third-grader. Authorities say the boy brought a .45-caliber handgun he got from his mother's house to an elementary school in Bremerton on Wednesday, and the weapon discharged from inside his backpack just before classes let out, critically injuring an 8 year-old girl. (*Father: 'My kid made a mistake' in school shooting*, **CBS News, February 24, 2012)**

Murfreesboro, TN
2/21/2012
A 14 year old boy was shot twice in the leg on the grounds of Carson Lane Academy. The shooting occurred just after 5 o'clock on a Monday afternoon during an altercation between two groups of kids. (*Police Make Arrests In Murfreesboro School Shooting*, **NewsChannel5, February 21, 2012**)

Brownsville, TX
1/4/2012
A male 8th grade Cummings Middle School student brandished a weapon today at approximately 8 AM in the school's main hallway. School administrators immediately contacted the Brownsville Police Department and BISD Police and Security Services. The school initiated standard lock down procedures. Law Enforcement Officers responded to the scene immediately. The student engaged the officers and was shot. He was transported to a local hospital by ambulance. No other students or employees were injured. (*Student gunman dies after school shooting in Brownsville,* **ValleyCentral.com, January 4, 2012**)

Berkeley, CA
11/15/2011
At 2:17 p.m., the UCPD got a 911 call about a man who reportedly had a gun in a backpack at the Haas School of Business. At 2:19 p.m., three officers arrived at the school and found the suspect in a computer lab on the third floor. The suspect pulled a gun out of his backpack, the police chief said, and displayed it "in a threatening manner." The chancellor said there were four students in the vicinity of the suspect and the officers, and that "it appeared students' lives might be at risk." After the officers told the man "numerous times" to drop his weapon, one of the three officers fired at the suspect, Celaya said. No one else was injured in the incident. (*Campus officials provide details about Haas School shooting,* **UC Berkeley News Center, November 15, 2011**)

Fayetteville, NC
10/25/2011
Two teenagers were in custody in the shooting of a 15-year-old fellow student who was wounded in the neck during a lunch period outside her North Carolina high school, and a sheriff said Monday the pair will be charged. Abercrombie was in stable condition after surgery at Cape Fear Valley Medical Center, according to Butler, who said she was standing in a breezeway outside the school cafeteria when she was shot.(*Cape Fear High School Shooting: 1 Student Shot, Wounded At North Carolina School,* **Huffington Post, October 25, 2011**)

Baltimore, MD
10/14/2011

A man was shot several times in the back in the parking garage on the University of Maryland at Baltimore. The shooting happened at 6pm, and the gunman fled. The man shot was taken to the nearest hospital in critical condition. The school enacted its "shelter in place" emergency protocol, and by 7:30 people were allowed to move about the buildings freely and cars were allowed to leave the parking lot. (*Man shot several times in underground parking garage in Baltimore*, **AP, October 14, 2011**)

Harper Woods, MI
8/26/2011
A 15-year-old girl was wounded when shots were fired at the conclusion of a football game at Chandler Park Academy in Harper Woods. When police arrived, witnesses said at least two people had been shot, but none of the victims remained at the scene. Detroit police contacted Harper Woods authorities to report a 15-year-old girl, who was shot in the stomach in the academy's parking lot after the game, was taken or walked to a nearby house in Detroit. An ambulance was called and transported the teenager to Children's Hospital in Detroit. (*Harper Woods Police Investigating Shooting after High School Football Game,* **Detroit News, August 29, 2011**)

Mandeville, LA
8/8/2011
Three 15-year-old New Orleans-area boys were arrested for allegedly plotting to shoot at least one student at Lakeshore High School and to kill law enforcement officials who responded to the scene. Authorities said the boys named their group "Day Zero." They had reportedly planned to acquire several guns and bring them to the school. Police collected information from computers in the boys' homes after other students told a school administrator about the alleged plot. (*Monday School Shooting Plot Broken Up, say La. Cops, Three Teens Arrested*, **CBSNEWS, August 8, 2011**)

Pearl City, HI
5/23/2011
A student was injured when a .45 caliber Glock semi-automatic pistol accidentally went off inside a middle school. The gun was unintentionally fired by another student who had brought the gun in to school to show off to friends. The bullet was shot through one student's jacket and ricocheted off a wall before inflicting nonlethal injuries on the victim. (*Accidental Shooting in Middle School Injures 1,* **KITV 4 Honolulu, May 23, 2011**)

Cleveland, OH
05/23/2011
A robbery attempt turned violent when a 16-year-old Benedictine High School student, Sophomore Jackrell Russell, was shot in the chest by a robber. The teenager was approached by the suspect who demanded the boy's cell phone and school-issued laptop. Jackrell was able to flag down a passing RTA bus where a passenger called 9-1-1. He was taken to a hospital and stabilized. (*Benedictine Student Shot In Chest During Robbery Attempt Monday Morning, Seeks Safety On RTA Bus,* **CLEVELAND PLAIN DEALER, May 24, 2011**)

Houston, TX
04/19/2011
Officials say three students were injured after a 6-year-old brought a loaded gun to his Houston elementary school that accidentally discharged when it fell out of the child's pocket. None of the injuries appeared life-threatening. *(6-Year-Old Brings Gun To Texas School, 3 Hurt*, **SALON, April 19, 2011**)

Los Angeles, CA
04/15/2011
A teenager was in custody today for allegedly shooting a 15-year-old boy who was shot as he walked to Los Angeles High School. A teenage suspect was later arrested and taken to a juvenile lockup. Investigators say the shooter and the victim, both boys, knew one another. The wounded boy suffered a bullet wound to the buttocks. A motive for the shooting was not disclosed. (*Teenager Taken Into Custody For Los Angeles High School Shooting*, **BEVERLY HILLS COURIER, April 16, 2011**)

Apopka, FL
04/13/2011
One person suffered a non-life-threatening injury after a shooting outside Sheeler Charter High in Apopka. The victim and two others, including one student, were taken into custody. The victim was shot in the abdomen. Investigators believe the suspects, who do not attend the school, came to the school to confront a student. (*2 Teens Facing Charges after Shooting Outside School,* **WFTV, April 13, 2011**)
Nashville, TN,
04/12/2011

A 17-year-old girl was shot and injured when shots were fired into her school bus. Two brothers, 18 and 19-years-old, were charged in the shooting outside Pearl Cohn High School. Police say the suspects' intended target was standing outside the bus at the time of the shooting. (*Teen injured in Pearl Cohn shooting,* **WKRN – TV 2 NASHVILLE, April 13, 2011**)

Opelika, AL
04/06/2011
A 63-year-old woman was killed and three other people were wounded by a gunman who opened fire outside the Higginbotham Academic Center at Union State Community College. Investigators arrested a 34-year-old man who admitted to the shooting which is believed to be domestic related. A 36-year-old woman who was a student at the college was hurt along with a 94-year-old woman and a four-year-old child who was injured by shattered glass. The shooting took place while students were changing classes. (*One Hurt After Shooting At Apopka Charter School For At-Risk Students,* **ORLANDO SENTINEL, April 14, 2011**)

Houston, TX
03/31/2011
One person was killed and five others were injured during a shooting -- believed to be gang-related -- just before 7 p.m. at Worthing High School during a powder puff football game. Eyewitnesses said a car, which police believe carried gang members, drove onto the athletic field and started shooting. Six people were shot, one of whom was pronounced dead at a nearby hospital. The identity of the victim has not been released. The suspects fled the scene but police managed to track down a gold Ford Taurus with blood stains on its doors about five miles from the Texan high school. At least one person was taken into custody. (*One Dead, Five Injured After Houston School Shooting,* **KETK, April 1, 2011**)

Martinsville, IN
03/25/2011
A shooting suspect was apprehended and police searched for a gun hours after a shooting at a middle school in which a student was injured. The shooting took place at Martinsville West Middle School. The victim was shot twice in the stomach. The shooting involved a student who was recently either expelled or suspended from school, authorities said.

(*Student Hurt, Suspect Arrested in School Shooting,* **WRTV-6, March 25, 2011**)

Los Angeles, CA
02/23/2011
A student was arrested for shooting and killing his instructor at the Coast Career Institute vocational school. The shooting took place inside a school classroom right in front of the class. The 22-year-old gunman was training to become a security guard. He fired at least 10-rounds from a semi-automatic handgun at point-blank range then went outside and waited until police arrived. (*Suspect briefly left security guard class before returning and opening fire, police say,* **LOS ANGELES TIMES, February 23, 2011**)

Murfreesboro, TN
02/14/2011
A fight led to a shooting at Middle Tennessee State University. The shooting suspect, a 20-year-old MTSU junior, was arrested. The 20-yead-old victim, a former MTSU student, was shot in the hand when the bullet ricocheted off the ground and hit his thumb. The campus was put on alert for nearly 45-minutes until the gunman was apprehended. (*Shooting at Middle Tennessee State University slightly injures man,* **THE TENNESSEAN, February 15, 2011**)

Youngstown, OH
02/06/2011
One person was killed and 11 were injured in a shooting at an after-hours party near the Youngstown State University campus. The deceased was a university student. Six of the injured were also students at the university. Two suspects were arrested. (*2 suspects charged in Youngstown fraternity shooting,* **CLEVELAND PLAIN DEALER, FEBRUARY 06, 2011**)

Placerville, CA
02/02/2011
A disagreement between a custodian and an elementary school principal over the hiring of a nighttime janitor led to the principal's fatal shooting. The shooter was told to leave Louisiana Schnell Elementary School earlier in the day but later came back to the school, went to the principal's office, and fired two rounds killing the principal. No children were hurt. (*Suspect,*

principal quarreled before Placerville School shooting, **THE HERALD - MONTEREY CO., February 04, 2011**)

Los Angeles, CA
01/18/2011
A 15-year-old girl and a 15-year-old boy were hurt when a 9-mm Beretta inside a fellow student's backpack went off at Gardena High School. Both victims were hit by the same bullet. The bullet hit one victim in the head and the other in the neck and shoulder. The 17-year-old suspect was already on probation for a misdemeanor battery charge. (*Girl critical after Los Angeles school shooting,* **USA TODAY, January 18, 2011**)

Omaha, NE
01/05/2011
A 17-year-old student shot the principal and assistant principal at Millard South High School before fleeing the school and shooting and killing himself. The assistant principal later died of her injuries. The gun used by the teen was a Glock .40 believed to be the service weapon of his father, an Omaha police officer. (*Millard South shooting: Suspension ignited fury,* **OMAHA WORLD-HERALD, January 05, 2011**)

Panama City, FL
12/14/2010
Ex-convict Clay Duke held a Florida school board a gunpoint firing several shots before shooting himself. No one else was hurt. The entire incident was caught on video. (*School board shooting: Clay Duke turns gun on himself after confrontation,* **WASHINGTON POST, December 15, 2010**)

Aurora, CO
12/06/2010
A 17-year-old female student was shot in the torso outside Aurora Central High School as she talked with classmates. Witnesses say two shots were fired into the crowd of students. No one else was hurt. The shooting prompted a lockdown at the high school, five elementary schools and a middle school. A 19-year-old boy was arrested in connection with what police said may have been a gang-related shooting. (*Female student, 17, shot outside Aurora Central High School,* **Denver Post, December 8, 2010**)

Garden City, SC
12/04/2010
A person leaving basketball practice at Groves High School was hit by a bullet when someone fired multiple shots at two cars. The victim was treated and released from a local hospital. The victim, who had been watching basketball practice, was not a student at the school. (Arek Sarkissian II, *Man shot after watching basketball practice at Groves High School, police say,* **SAVANNAH MORNING NEWS, December 7, 2010)**

Chicago, IL
11/29/2010
A 17-year-old Thornridge High School student was shot and injured during an armed robbery outside the school in south suburban Dolton. The shooting occurred after school hours. Police arrested a 14 and a 15-year-old in connection to the crimes. The pair fled after the shooting but authorities apprehended them a short time later. (*Student Shot at South Suburban High School*, **CHICAGO SUN TIMES, November 30, 2010**)

Marinette, WI
11/28/2010
A 15-year-old student shot a film projector and held 23 classmates and a teacher hostage for about five hours at Marinette High School. The shooter had two pistols, knives and more than 200 rounds of ammunition. He fatally shot himself when police broke down the classroom door. (*Answers sought in northern Wis. hostage situation*, **ASSOCIATED PRESS, December 2, 2010**)

Fairfield, CA
10/11/2010
A 29-year-old man was shot to death in the parking lot of Armijo High School. Two people, including a 19-year-old, were arrested. Police said the shooting was not connected to the high school campus. Investigators believe the fight was arranged by gangs intent on settling a feud. (*2nd Suspect Arrested In Fairfield School Shooting,* **CBS 13 SACRAMENTO, November 24, 2010**)

Selma, AL
10/25/2010

Shots were fired into a classroom at Payne Elementary School from outside after school was over for the day. A teacher was in the classroom but was unharmed. Nobody was injured. (*Selma Police Look for School Shooting Suspect*, **WSFA - TV 12 MONTGOMERY, October 26, 2010)**

Carlsbad, CA
10/8/2010
A man jumped the fence at Kelly Elementary School and opened fire on children playing outside at lunchtime. When his gun jammed, nearby construction workers tackled him. Two girls, ages six and seven, were injured. Neighbors report the shooter often screamed obscenities and racial epithets, and that the police had been called repeatedly. (*Workers hailed for halting school shooting suspect*, **ASSOCIATED PRESS, October 11, 2010**)

Elizabeth City, NC
10/3/2010
A student fatally shot another student in the victim's dorm room at Mid-Atlantic University on Sunday afternoon. The University said the shooting stemmed from a conflict between the two individuals. (*Police: NC Bible college student killed in dorm*, **ASSOCIATED PRESS, October 4, 2010**)

Austin, TX
9/28/2010
A 19-year-old student fatally shot himself in the main library at the University of Texas with an AK-47 assault rifle. He ran through parts of the campus and fired several shots into the air before entering the library and killing himself. (Russell Goldman, *Gunman at University of Texas Austin Identified as Sophomore Math Major Colton Tooley*, **ABC NEWS, September 28, 2010**)

Columbia, SC
9/21/2010
A student confronted and shot at a resource officer at Socastee High School. The officer sustained minor injuries as the bullet hit the officer after ricochet off a wall. The student, working with another student, had also planted pipe bombs around the school. The bombs were removed and disarmed by police without injury. (Susanne M. Schafer, *SC principal: 2nd student questioned in shooting*, **ASSOCIATED PRESS, September 22, 2010**)

Moss Bluff, LA
9/16/2010
A student fatally shot himself at St. Theodore/Holy Family School at the start of the school day. (Kate Mundy, *Bishop Releases Statement on School Shooting*, **KATC- TV 3 LAFAYETTE, LA, September 16, 2010**)

Las Cruces, NM
9/7/2010
A 17-year-old fired a handgun during a fight between several teenagers during a volleyball game at Las Cruces High School. (*3 More Teens Arrested In Las Cruces High School Shooting Incident*, **KVIA – TV 7 EL PASO, September 14, 2010**)

Detroit, MI
9/7/2010
A 14-year-old girl and a 16-year-old boy were shot outside Mumford High School on their first day of the school. A fight had began inside the school but spilled outside. (*Mumford High School Shooting: 2 Students Shot In Detroit*, **HUFFINGTON POST, September 7, 2010**)

Omaha, NE
8/18/2010
A 15-year-old fired a gun on a school bus on the way home from Burke High School. Nobody was injured. Officials were unaware of the incident until it was reported by a parent the next day. A 16-year-old was found with a gun at the same school the next day. (*Gun Fired Aboard Omaha School Bus*, **KETV – TV 7 OMAHA, August 19, 2010**)

South Gate, CA
5/18/2010
A 17-year-old shot a 15-year-old student in the abdomen at South Gate High School. The shooter attended a neighboring high school. (*Teen held for LA-area school shooting*, **ASSOCIATED PRESS, May 18, 2010**)

Portsmouth, VA
4/29/2010
A student entered Wilson High School through a back door and fired several shots inside the school. Nobody was injured. (Cheryl Ross & Janie

Bryant, *After shooting, Wilson High students wary about security*, **VIRGINIAN-PILOT, April 30, 2010**)

Baton Rouge, LA
3/23/2010
Four middle school students were arrested for bringing a gun onto school grounds and firing it near a practice field. No one was injured during the incident. (*Deputies arrest 4 students after school shooting*, **WAFB-TV 9 BATON ROUGE, March 26, 2010**)

Columbus, OH
3/9/2010
A disgruntled Ohio State University maintence employee, who had recently been fired from his job, opened fire in a campus facilities building, killing a maintenance manager and wounding another co-worker before he turned the gun on himself. The shooter had been in prison previously. (Leslie Tripp,*Suspect kills self in Ohio State shooting, police say*, **CNN, March 9, 2010**)

Littleton, CO
2/23/2010
A 32-year-old man shot at students as they left Deer Creek Middle School at the end of the day. Two students were injured before a math teacher tackled the shooter. The shooter used his father's rifle in the attack. (Nicholas Riccardi, *Math teacher hailed as hero after Colorado school shooting*, **LOS ANGELES TIMES, February 25, 2010**)

DeKalb, IL
2/19/2010
One student was injured and another placed into police custody after a shooting on the campus of Northern Illinois University. The shoting was caused by an altercation between the shooter and victim. (*NIU Shooting Leaves 1 Hurt, 1 in Custody*, **ASSOCIATED PRESS, February 19, 2010**)

Huntsville, AL
2/12/2010
A professor opened fire 50 minutes into at a Biological Sciences Department faculty meeting at the University of Alabama, killing three colleagues and wounding three others. The shooter had a history of

violence: she shot and killed her brother in 1986, was possibly involved in an attempted mail bombing, and was convicted of disorderly conduct, and assault and battery in an incident in 2002. The gun used in shooting was bought from the shooter's husband in 1989 by a friend in New Hampshire to avoid a waiting period in Massachusetts. (Emanuella Grinberg, *Judge sends Alabama university shooting case to grand jury*, **CNN, March 24, 2010.** Aaron Cooper & Brooke Baldwin, *Police: University shooting suspect was charged after fight in 2002,* **CNN, February 17, 2010**)

Knoxville, TN
2/10/2010
A teacher shot and wounded the principal and assistant principal in their offices at Inskip Elementary School, where all three worked. (Tonja Bur, *Knoxville elementary principal Elisa Luna, injured in school shooting, trasnferred to Atlanta hospital*, **IR-TV 10 KNOXVILLE, February 17, 2010**)

Madison, AL
2/5/2010
A 14-year-old student was shot and killed between classes at Discovery Middle School. The suspect in police custody is a fellow ninth grade student. (Yvonne T. Betowt, M*adison coming to grips with Discovery Middle School shooting*, **THE HUNTSVILLE TIMES, February 7, 2010**)

Livingston, AL
1/20/2010
A high school teacher was shot multiple times outside her school in what police believe was a domestic incident. The suspect was caught 30 miles from the scene after causing a traffic accident. (Wayne Grayson *Livingston teacher shot outside school*, **THE TUSCALOOSA NEWS, January 21, 2010**)

Phoenix, AZ
1/7/2010
A 16 year old former student shot three students outside Esperanza Charter School. The shooter argued with two 19 year olds and then shot them. A 10 year old boy was also grazed with a bullet. Police believe the shooting was gang related. (*Police: Triple shooting near north Phoenix school is gang related*, **ASSOCIATED PRESS, January 8, 2010**)

Shreveport, LA

12/11/2009
A 28 year old man shot an 18 year old female student multiple times outide of Shreveport High School. The shooter waited at the school for her to arrive in the morning and is believed to have been involved with the teenager. The victim is expected to survive. (*Teenage girl shot at Shreveport high school*, **SHREVEPORT TIMES, December 11, 2009**)

Woodbridge, VA
12/8/2009
A 20 year old student fired twice at his math professor during class at Northern Virginia Community College. The shooter then put his gun down, left the classroom and was arrested without incident. Nobody was injured. (Nafeesa Syeed *No one hurt in college classroom shooting*, **ASSOCIATED PRESS, December 9, 2009**)

Brockton, MA
12/2/2009
A 17 year old former student was shot in both legs late in the afternoon at Brockton High School. He was shot near the gymnasium where approximately 100 students were participating in basketball tryouts. (John M. Guilfoil, *Man is shot at Brockton High School*, **BOSTON GLOBE, December 2, 2009**)

Boston, MA
11/17/2009
A 22 year old man was shot while playing basketball in the evening at a elementary school. Police believe the shooting was gang-related. (*Boston police call Dorchester shooting gang-related*, **BOSTON GLOBE, Nove. 18, 2009**)

Dallas, TX
11/16/2009
A Dallas schools maintence man visiting Umphrey Lee Elementary School accidentally shot himself in the leg while talking with a custodian. No children were nearby when the shooting occurred. (Tawnell Hobbs, *DISD employee was accidental shooter at Umphrey Lee*, **DALLAS MORNING NEWS, November 16, 2009**)

Philadelphia, PA

11/6/2009
A 15 year old shot a 17 year old fellow student in the head on the school bus to CEP Miller School before fleeing. He was later arrested and found in possession of a handgun. Police believe the shooter was retaliating for being bullied by the victim. (Allison Steele and Peter Mucha, *Teen held after shooting on Philadelphia school bus*, **PHILADELPHIA INQUIRER, November 7, 2009**)

Pineville, LA
11/1/2009
A 19 year old fatally shot himself in the head with an SKS assault rifle. He was at a party with a group of people who were "playing" with the gun at 3am. (*Pineville man killed in accidental shooting*, **CENTRAL LOUISIANA TOWN TALK, November 2, 2009**)

Memphis, TN
10/24/2009
Four teenagers shot an AK-47 assault rifle in a wooded area behind Craigmont High School while school was in session. Two of the suspects, who were not students at the school, were parked outside the school in the fire lane, and apprehended by police with the weapon and a 30 round magazine. (Ryan Poe, *Teen charged after rifle fired near school*, **MEMPHIS COMMERCIAL APPEAL, October 24, 2009**)

Long Beach, CA
10/20/2009
A 16 year old female student was shot and killed, and two non-students, ages 18 and 20, were injured when someone opened fire as hundreds of people were leaving a Friday night football game at Woodrow Wilson High School. There was a school dance, with approximately 200 students, also happening at the school at the time of the shooting. (*Girl shot dead after Calif. school football game*, **ASSOCIATED PRESS, November 1, 2009**)

Southold, NY
10/8/2009
A 28 year old man fired a bullet that went through a window of Mattituck High School and grazed the head of an 18 year old student sitting in class. The bullet was possibly fired from his home that is located near the school.

(Jennifer Sinco Kelleher, *Man held in Mattituck H.S. shooting, but not charged*, **Newsday, October 9, 2009**)

Henrico, VA
9/16/2009
A 16 year old student with a handgun fired multiple shots in the parking lot of Virginia Randolph Community High School while school was in session. The gunman fled on foot, but was arrested without incident by police a short while later. Nobody was injured. (Bill McKelway, *Student arrested in high school parking lot shooting*, **Richmond Times-Dispatch, September 16, 2009**)

Grand Rapids, MI
9/14/2009
A 16 year old student fired a shot into air in the parking lot of Creston High School. Nobody was injured. The next day another student was caught with a sawed off shotgun in his pants leg at the same school. (*Editorial: Freedom from fear*, **Grand Rapids Press, September 19, 2009**)

Stamford, CT
9/8/2009
A 16 year old shot at a fellow student outside of Stamford Academy, a charter high school. Police believe the shooter and target had a previous dispute. (Chase Wright, *Teen charged in shooting at Stamford Academy*, **Stamford Times, September 9, 2009**)

Atlanta, GA
9/3/2009
Someone fired a gun multiple times during a fight on the campus of Clark Atlanta University, fatally injuring a 19 year old student walking in the area with friends. The victim went to neighboring Spellman College. (*Stray bullet kills college student on Ga. campus*, **Associated Press, September 3, 2009**)

San Bruno, CA
9/2/2009
One person got shot during a fight in a parking lot at Skyline College. (Joshua Melvin and Sean Maher, *Police arrest three men in Skyline College shooting*, **San Mateo County Times, September 3, 2009**)

Houston, TX
7/10/2009
Six people were shot, including one student, in a drive by shooting at a community rally on the campus of Texas Southern University. Police believe the shooting to be gang related. (Juan A. Lozano, *6 shot, wounded in drive-by at Texas Southern*, **Associated Press, July 24, 2009**)

Parkersburg, IA
6/24/2009
A 24 year old man shot and killed his former high school football coach in the weight room of Aplington-Parkersburg High School. Several students were in the weight room at the time of the shooting, but no one else was injured. The shooter was arrested at a nearby home. (Nigel Duara, *Former player charged with killing Iowa coach*, **Associated Press, June 24, 2009**)

Lexington, KY
6/9/2009
A school employee shot and killed another employee at Leestown Middle School. School had ended for the year and no students from the school's summer programs were present during the shooting. The victim and shooter had clashed in the past. (Cu*stodian dead, another sought in Lexington school shooting*, **Associated Press, June 9, 2009**)

Cambridge, MA
5/18/09
A 21 year old man died after being shot at a Harvard University dormitory during an attempted drug robbery. Two men have been arrested in connection with the shooting. Neither the victim nor the assailants were students at Harvard. (Andrew Ryan and Tracy Jan, *2nd man faces murder charge in Harvard dorm shooting*, **Boston Globe, June 11, 2009**)

Larose, LA
5/18/2009
A 15 year old fired a shot at a teacher before fleeing to a bathroom and shooting himself in the head at Larose-Cut Off Middle School. The teacher was not hit. The boy died several days later. (*Sheriff: Teen school shooter's donated organs saves two lives*, **WWL – TV 4 New Orleans, May 27, 2009**)

West Covina, CA
5/4/2009
Two teenagers, ages 15 and 16, were charged with conspiracy to commit murder after plotting to shoot their classmates during a school assembly at Covina High School. Police found two loaded handguns at the home of the 15 year old, and believe the teens stole them from the 16 year old's stepfather. The police began investigating when the stepfather reported his handguns missing over a month ago. (*Teens Plead Not Guilty to Plotting School Shooting*, **KTLA – TV 5 LOS ANGELES, May 4, 2009**)

Sheboygan, WI
5/1/09
A troubled 17 year old shot himself in the stomach in the parking lot of Sheboygan North High School. (*Shooting at Sheboygan High School*, **WGBA – TV 26 GREEN BAY, May 1, 2009**)

Hampton, VA
4/26/2009
An 18 year old former student followed a pizza deliveryman into his old dormitory, and shot the deliveryman, a dorm monitor, and himself at Hampton University. No students were injured and all of those who were hurt are expected to survive. (*3 Va. men injured in shooting at Hampton University*, **ASSOCIATED PRESS, April 27, 2009**)

Dearborn, MI
4/10/2009
A man shot and killed a female classmate and then himself at MacKenzie Fine Arts Center on the campus of Henry Ford Community College. (Melanie D. Scott, *Shots on campus end big dreams*, **DETRORIT FREE PRESS, April 11, 2009**)

Dove Creek, CO
4/9/2009
Two teens, who were arrested in New Mexico for burglary, had planned a shooting spree at Dove Creek High School in Colorado. The teens also planned to shoot the school's principal and the superintendent, as well as the County Sheriff and Undersheriff. Police found three guns in the

teenagers' possession and seven more guns at the younger boy's home. (P. Solomon Banda, *Teens accused of shooting plot at rural Colorado high school; authorities find weapons*, **ASSOCIATED PRESS, APRIL 9, 2009**)

Jacksonville, FL
3/10/2009
At least one shot was fired during a fight that erupted among several students at Ribault High School before classes began for the day. Nobody was injured; two guns were recovered. (*6 students in custody after shot fired*, **UNITED PRESS INTERNATIONAL, March 10, 2009**)

Detroit, MI
2/17/2009
A former student snuck into Central High School and was shot by another non-student at the end of the school day. (Jennifer Mrozowski, *Shooting at Detroit high school leaves victim, suspect hospitalized*, **DETROIT NEWS, February 17, 2009**)

Wake County, NC
2/12/2009
A boy pulled a pistol out of his backpack and accidentally shot a 14 year old classmate in the leg while the two were on the bus to Zebulon Middle School. (Thomasi McDonald and T. Keung Hui, *Teen grazed on Wake school bus: Victim OK; friend's father is charged*, **RALEIGH NEWS & OBSERVER, February 12, 2009**)

El Monte, CA
2/11/2009
A third grader accidentally fired a gun while showing it to friends on the playground of Baker Elementary School. Nobody was injured, but there were approximately 100 other kids on the playground at the time of the shooting. The boy says he took the gun from his grandmother. (*Safety questioned after school shooting*, **KABC- TV 7 LOS ANGELES, February 11, 2009**)

Clayton, NC
1/27/2009
Two teenagers were arrested for firing a gun in the parking lot of Clayton High School. A bullet struck the outside wall of the gymnasium, where a

basketball game was in progress. (*Clayton Police Arrest Suspects In Clayton High School Shooting*, **WNCN – TV 17 NORTH CAROLINA, February 16, 2009**)

Chicago, IL
1/9/2009
A gunman began shooting indiscriminately from a car into a crowd of people that were leaving a basketball game at Paul Laurence Dunbar Vocational Career Academy. Five people were injured. (Rupa Shenoy, *5 people shot outside Chicago high school*, **ASSOCIATED PRESS, January 9, 2009**)

New Castle, DE
1/8/2009
One person was shot and injured outside of William Penn High School after a basketball game. (Vince Lattanzio, *One person Shot at William Penn High in Del*, **WCAU - TV 10 PHILADELPHIA, January 8, 2009**)

North Manheim, PA
12/18/2008
A 17 year old was arrested for plotting to shoot students at Blue Mountain High School. Police found multiple weapons and paramilitary gear at his home. (*Schuylkill County student charged with planning school shooting*, **HARRISBURG PATRIOT-NEWS, December 19, 2008**)

Montco, PA
12/4/2008
A 15 year old was institutionalized after stealing three guns and hundreds of rounds of ammunition from his father, and plotting to shoot other students and himself at Pottstown High School. Police began investigating when the father reported the guns stolen. The boy later admitted to the shooting plot and pled guilty to attempted first degree murder. (*Montco teen accused of school-shooting plot*, **PHILADELPHIA DAILY NEWS, De. 10, 2008**)

Savannah, GA
11/21/2008
A 19 year old student shot a fellow student twice after the two argued at Savannah State University. All staff and students were notified and buildings on campus were locked down until the shooter was arrested.

(*Suspected campus shooter at Savannah State caught*, **Associated Press, November 21, 2008**)

St. George, UT
11/15/2008
A 15 year old student died from injuries after the gun he was holding discharged at Desert Hills High School. The gun was a prop for the school play and was loaded with blanks. (*District wants to know how student got his hands on gun*, **KSL – TV 5 Utah, November 17, 2008**)

Ft. Lauderdale, FL
11/12/2008
A 15 year old female student shot and killed a 15-year-old classmate in a hallway at Dillard High School. The two girls had been friends for years, but had issues recently. The shooter may have gotten the gun from her grandfather, her guardian, who had a concealed carry permit. (*"I wanted her to feel pain like me," accused Dillard High shooter tells police,* **Miami Herald, November 13, 2008**)

Big Bear, CA
10/29/2008
Five teenage boys were arrested for plotting to shoot students, teachers, and staff at Big Bear High School. Other students overheard their plans and alerted authorities. (*5 students held in shooting plot at Big Bear High School*, **Los Angeles Times, October 30, 2008**)

Conway, AR
10/26/2008
Several men in a car drove up to a dormitory at the University of Central Arkansas and opened fire, killing two students and injuring a third person. The police believe the victims were targeted. (*2 killed at Ark. school; officials say campus safe*, **Associated Press, October 27, 2008**)

San Antonio, TX
10/13/2008
A librarian shot and killed a fellow librarian at Northeast Lakeview Community College library, where the two men worked. (*Librarian accused of shooting dead fellow librarian*, **Associated Press, October 14, 2008**)

Willoughby, OH
9/2/2008
A 15 year old student fired two shots in a hallway at Willoughby South High School. It is believed the boy planned to kill himself in front of his girlfriend. (*Teen fires gun in high school, surrenders without bloodshed*, **CLEVELAND PLAIN DEALER, September 2, 2008**)

Portland, OR
8/29/2008
An unidentified shooter fired five or six shots into a crowd leaving a football game at Madison High School. Shortly before the shooting, police arrested a 14 year old for carrying a gun in a separate incident nearby. (*Possible gang-related shooting at Madison High worries police*, **PORTLAND OREGONIAN, September 2, 2008**)

Opelousas, LA
8/26/2008
Three students were arrested after shots were fired at the T.H. Harris campus of Louisiana Technical College. No one was injured. (*3 arrested after shots fired on campus*, **WXVT - TV 15 MISSISSIPPI, August 27, 2008**)

Knoxville, TN
8/21/2008
A 15 year old fatally shot a fellow 15 year old classmate in the cafeteria at Central High School. (*Student killed in shooting in Tenn. School*, **ASSOCIATED PRESS, August 21, 2008**)

Phoenix, AZ
7/24/2008
A former student shot three people in a computer lab at South Mountain Community College. The gunman had a longstanding disagreement with one of the victims. (*Phoenix Shooting: Suspect Arrested*, **ASSOCIATED PRESS, July 25, 2008**)

Boca Raton, FL
4/30/2008
During a fight at a campus party at Florida Atlantic University, a man fired two shots in a crowded room, one grazing the ear of a 21-year-old college student. The shooter claimed that the victim and a friend “were looking at

him like they wanted a fight.” (*FAU Suspect Held on $35,000 Bail*, **SOUTH FLORIDA SUN SENTINEL, May 2, 2008**)

Washington, DC
4/29/2008
A student shot two people at Excel Institute, a vocational school, and then stole two cars as fled from the police. (*2 Shot at NE Vocational School*, **WASHINGTON POST, April 30, 2008**)

Omaha, NE
4/24/2008
An eighth grader was shot in the face during a soccer game at King Science and Technology Magnet Middle School. (*Police: King School Shooting, Druid Hill Scene Likely Related*, **KETV-TV 7 OMAHA, April 25, 2008**)

Hayward, CA
3/31/2008
A 17 year old boy was shot in the leg at Royal Sunset Continuation School. Three other students are believed to have been involved in the shooting. (*Boy shot in the leg at Hayward school*, **ASSOCIATED PRESS, March 31, 2008**)

Chicago, IL
3/29/2008
An 18 year old died after being shot by a 17 year old and a 19 year old outside Simeon Career Academy shortly after Saturday classes let out. There had been a several fights leading up to the shooting. (*Series of fights led to Simeon shooting*, **CHICAGO SUN TIMES, March 29, 2008**)

Chicago, IL
3/7/2008
A 15 year old fatally shot an 18 year old student outside Crane High School. (*Community Outraged After 16th CPS Student Death This School Year*, **WMAQ-TV 5 CHICAGO, March 10, 2008**)

Mobile, AL
3/6/2008

A student shot and killed himself in front of 150 other students in the gym of Davidson High School. (*Shooting Confirmed at Davidson High School in Mobile*, **WVTM-TV 13 BIRMINGHAM, March 6, 2008**)

Miami Gardens, FL
2/28/2008
A bullet grazed the ear of a 17 year old student as he was leaving band practice at Miami Norland Senior High School. (*Bullet Grazes 12th Grader's Ear*, **WPLG-TV 10 MIAMI, February 28, 2008**)

Little Rock, AR
2/27/2008
A student was shot on the campus of the University of Arkansas at Little Rock. (*Student Hurt in University Shooting*, **MORNING NEWS, February 27, 2008**)

Jonesboro, AR
2/23/2008
A student was injured when a bullet ricocheted off a building and hit him in the leg on the campus of Arkansas State University. The shooter has not been identified. (*Investigation Continues into ASU Shooting*, **ASSOCIATED PRESS, February 25, 2008**)

DeKalb, IL
2/14/2008
A former graduate student armed with mulitple guns entered a lecture hall at Northern Illinois University and began shooting. He killed five students and wounded 16 before killing himself. (*Gunman Was Once 'Revered' on Campus*, **NEW YORK TIMES, February 15, 2008**)

Oxnard, CA
2/12/2008
A 14 year old fatally shot another student in the head while in class at E.O. Green Junior High School. The victim had been bullied at school since declaring that he was gay several weeks earlier. (*Shooting sparks call for changes*, **LOS ANGELES TIMES, February 20, 2008**)

Memphis, TN
2/11/2008

A 17 year old shot another student multiple times before handing the gun over to the teacher during gym class at Mitchell High School. The two students had argued over the weekend. There were about 75 students in the room during the shooting. (*Tenn. School Shooting Stemmed From Fight*, **ASSOCIATED PRESS, February 11, 2008**)

Baton Rouge, LA
2/8/2008
A female student killed herself and two others in a classroom at Louisiana Tech. (*Woman Kills 2, Herself at La. College*, **ASSOCIATED PRESS, February 8, 2008**)

Memphis, TN
2/4/2008
A 16 year old student shot another 16 year old in the leg while in class at Hamilton High School. The injury is not critic. (*16-year-old shot, wounded at Memphis high school*, **ASSOCIATED PRESS, February 4, 2008**)

Washington, DC
1/22/2008
Four Ballou High School students were shot as they were leaving school at the end of the day. All students are expected to recover. (*3 shot shortly after high school dismissal*, **ASSOCIATED PRESS, January 22, 2008**)

Las Vegas, NV
1/18/2008
A 16 year old shot at another student outside a basketball game at Cheyenne High School. Nobody was injured in the incident. (*Teen Arrested in Cheyenne High School Shooting*, **KLAS - TV 8 LAS VEGAS, January 19, 2008**)

Charlotte, NC
1/16/2008
A student at Crossroads Charter High School was shot in the parking lot after an early-scheduled dismissal. (*Student Shot at North Carolina Charter School*, **ASSOCIATED PRESS, January 16, 2008**)

Cleveland, OH
1/16/2008

A 16 year old boy pulled a gun from his locker and aimed it into a crowd of students when a fight erupted at South High School. (*Teen Points Gun at Students at South High*, **WJW - TV 8 CLEVELAND, January 16, 2008**)

Putnam City, OK
1/15/2008
A 17 year old was shot three times in a Putnam City High School parking lot after a basketball game. (*Teen released after shooting outside high school*, **ASSOCIATED PRESS, Jan, 16, 2008**)

Asheville, NC
1/10/2008
A 16 year old with a handgun shot at a fellow student at Asheville High School. Nobody was injured in the incident. (*One dead, four wounded in M'ville hotel shooting*, **MUNSTER TIMES, December 30, 2007**)

Gibsonton, FL
12/17/2007
Three East Bay High School students shot two other students as they were walking home from their bus stop. The two groups of students had clashed previously. (*2 high-schoolers shot near Tampa*, **ASSOCIATED PRESS, December 18, 2007**)

Las Vegas, NV
12/11/2007
Two assailants using 9-millimeter and .45-caliber guns shot six people as they exited a Mojave High School bus. The attack followed a fight at school earlier in the day. (*6 People Shot After Exiting a School Bus in Las Vegas*, **ASSOCIATED PRESS, December 12, 2007**)

Oakland, CA
12/11/2007
Three teens were shot after a basketball game at McClymonds High School in a drive-by shooting. (*School looks beyond shooting: Drive-by triggers anti-violence lesson*, **OAKLAND TRIBUNE, December 14, 2007**)

Langrangeville, NY
11/29/2007

Three Hudson Valley High School students were arrested for planning a Columbine-type attack and making threats online. (*Hudson Valley High students arrested, charged with plotting school attack*, **Associated Press, November 28, 2007**)

Holland Patent, NY
11/26/2007
Two Holland Patent High School students shot a gun while on their school bus. Nobody was injured. (*Deputies: Holland Patent shooting no accident*, **TWEAN - TV 10 Syracuse, November 27, 2007**)

Beaufort, SC
11/20/2007
An 18 year old shot a 17 year old student in the Battery Creek High School parking lot after a basketball scrimmage. (*Police still searching for Battery Creek High School shooting suspect*, **Beaufort Gazette, November 22, 2007**)

Saginaw, MI
10/25/2007
Two Arthur Hill High School students and two adults were shot by another student as they left a middle school football game. (*Charges sought in shooting at Saginaw school football game*, **Associated Press, October 25, 2007**)

Portland, OR
10/12/2007
Two teens from Jefferson High School were shot when a gunman fired into a crowd of students that had gathered after the end of their Homecoming Dance. (*Jefferson High boosts security after shootings*, **KGW-TV Portland, OR, October 17, 2007**)

Cleveland, OH
10/10/2007
A 14 year old student shot two teachers and two students at SuccessTech Academy before killing himself. He had been suspended for fighting earlier in the week and had threatened to harm other students and blow up the school previously. (*Student, 14, Shoots 4 and Kills Himself in Cleveland School*, **New York Times, October 10, 2007**)

Norristown, PA
10/10/2007
A 14 year old is arrested for stockpiling weapons and plotting a Columbine-style attack. Police found over 30 weapons in his possession. (*Pa. Student Admits Stockpiling Weapons*, **ASSOCIATED PRESS, October 26, 2007**)

Oroville, CA
9/28/2007
A 17 year old armed with a .22-caliber handgun takes more than two dozen students hostage at Las Plumas High School, holding three of them for an hour before police convince him to surrender. (*Student Takes Hostages*, **LOS ANGELES DAILY NEWS, Sep, 28, 2007**)

Dover, DE
9/21/2007
A freshman at Delaware State University shot and wounded two other students at a campus dining hall. The shooter, who had earlier been in a fight with one of the victims, has been charged with attempted murder. (*Delaware State teen charged with attempted murder*, **CNN, September 24, 2007**)

Newark, NJ
8/4/2007
Three Delaware State University students were shot and killed execution style by a 28 year old and two 15 year old boys. The three friends were forced to kneel against a wall behind an elementary school and were shot in the head. A fourth student was found about 30 feet away with gunshot and knife wounds to her head. (*Third Suspect Arrested in Student Killings*, **ASSOCIATED PRESS, August 11, 2007**)

Dallas, TX
6/29/2007
A 17 year old former high school football player shot and injured two other high school students in the school parking lot. (*2006-2007 School Year Incidents*, **NATIONAL SCHOOL SAFETY AND SECURITY SERVICES, INC, last visited July 23, 2007**)

Huntersville, NC

4/18/2007
A 16 year old male high school student threatened two high school students with a gun in their school's parking lot and then turned the gun on himself, committing suicide, when police subsequently approached him in a nearby gas station. (*2006-2007 School Year Incidents*, **NATIONAL SCHOOL SAFETY AND SECURITY SERVICES, INC, last visited June 19, 2007**)

Blacksburg, VA
4/16/2007
A student killed 32 students and faculty, and wounded 15 more at Virginia Tech. He was armed with a Glock model 19 handgun and a Walther P22 handgun. It is the worst single act of gun violence in American history. (*Gunman Kills 32 at VA Tech in Deadliest Shooting in U.S. History*, **WASHINGTON POST, April 17, 2007**)

Gresham, OR
4/10/2007
Ten high school students were injured when gunshots from a rifle shattered a classroom window. A student who attended the school was later arrested and the rifle was found in a nearby field. (*2006-2007 School Year Incidents*, **NATIONAL SCHOOL SAFETY AND SECURITY SERVICES, INC, last visited June 19, 2007**)

Greensboro, NC
3/24/2007
A freshman was seriously wounded after being shot in the lower back with a .25 automatic pistol while in his dorm room at the University of North Carolina at Greensboro. A 19 year old arrested and charged with the shooting. (*UNGC Police say weekend shooting drug-related*, **GREENSBORO NEWS RECORD, March 29, 2007**)

Midland, MI
3/7/2007
A gunman shot and wounded his teenage ex-girlfriend in a high school parking lot in Midland, before fatally shooting himself. (*U.S. School Shootings*, **CBS NEWS, last visited June 19, 2007**)

Los Angeles, CA
1/26/2007

An 18 year old male was shot in the three times and died while playing basketball in a middle school gym. Police were seeking three suspects who fled after the shooting. (*2006-2007 School Year Incidents*, **National School Safety and Security Services, INC, last visited June 19, 2007**)

Tacoma, WA
1/3/2007
An 18 year old student shot a fellow student in the hallway of Henry Foss High School before classes began. (*Cops Seek Motive in Wash. School Shooting*, **CBS News, January 4, 2007**)

Springfield Township, PA
12/12/2006
A 16 year old male high school shot and killed himself with an AK-47 in the hallway of his high school. The student, reportedly despondent over his grades, had the gun concealed in a camouflage duffle bag and fired one round in the ceiling to warn other students to get out of the way before committing suicide. (*2006-2007 School Year Incidents*, **National School Safety and Security Services, INC, last visited June 19, 2007**)

Clinton, SC
11/9/2006
A North Carolina man, suspected of assaulting his wife, committed suicide after wounding a police officer on the campus of Presbyterian College. (*Man Shoots S.C. College Police Officer Before Killing Himself*, **Associated Press, November 10, 2006**)

Joplin, MO
10/9/2006
A 13 year old boy, obsessed with the Columbine school shootings, brought a MAC-90 semiautomatic assault rifle (a replica of an AK-47) to school, pointing it at students and firing it into the ceiling until the gun jammed. (*Missouri Boy Fires Rifle in his School; All Are Safe*, **New York Times, October 10, 2006**)

Nickel Mines, PA
10/2/2006
A dairy truck driver walked into a one-room Amish schoolhouse with a shotgun, a semi-automatic handgun, and 600 rounds of ammunition,

selected all the female students, and shot them execution-style, killing five and seriously wounding six. The man then shot himself, apparently having left suicide notes beforehand. (*Man Shoots 11, Killing 5 Girls, in Amish School*, **NEW YORK TIMES, October 2, 2006**)

Cazenovia, WI
9/29/2006
A student walked into a rural school with a handgun and a shotgun, and shot the principal several times, killing him. (*Principal Killed by Shot in Struggle With Angry Student*, **NEW YORK TIMES, September 30, 2006**)

Bailey, CO
9/27/2006
A lone gunman enters a high school and holds six female students hostage, sexually assaults them, kills one of them, and then kills himself after a four-hour standoff. (*Student and Gunman Die in Colorado High School Standoff*, **NEW YORK TIMES, September 28, 2006**)

Pittsburgh, PA
9/17/2006
Five Duquesne University basketball players are wounded, one critically, after a shooting on campus following a dance, the first such incident in the 128-year history of the University. (*2nd Arrest Is Made in Duquesne Attack*, **NEW YORK TIMES, September 21, 2006**)

Green Bay, WI
9/14/2006
Two boys, teased at school and obsessed with the mass killings at Columbine, are arrested for amassing an arsenal of guns and bombs and for planning an attack on East High School. (*Schoolmates Made Fun of Boys Charged in Plot; 2 Had Columbine Fascination, Police Say*, **WASHINGTON POST, September 23, 2006**)

Hillsborough, NC
8/30/2006
After shooting his father to death, a student opens fire at his high school, injuring two students. Deputies found guns, ammunition, and homemade pipe bombs in the student’s car. The student had emailed Columbine High’s Principal, telling him that it was “time the world remembered” the

shootings at Columbine. (*Teenager Is Accused of Multiple Shootings*, **NEW YORK TIMES, September 1, 2006**)

Essex, VT
8/24/2006
A gunman shoots five people, killing two of them, in a rampage through two houses and an elementary school, before wounding himself. (*Deadly Rampage in Quiet Vt. Town; Man Kills Two, Hurts Two Others, in Shootings at School, Homes*, **BOSTON GLOBE, August 25, 2006**)

Red Lake Indian Reservation, MN
3/21/2005
At the time, the worst school-related shooting incident since the Columbine shootings in April of 1999. Ten killed and seven injured in rampage by a student at a high school. (*A Very Quiet Sense of Shock'; Small Community Struggles to Cope*, **WASHINGTON POST, March 23, 2005**)

Bellflower, CA
3/16/2005
Another Columbine was averted when two juveniles were arrested for plotting to shoot up their high school. (*2 Boys Charged in Plot; Bellflower Teens Are Accused of Planning Killings at Their School*, **LOS ANGELES TIMES, March 16, 2005**)

Cumberland City, TN
3/2/2005
A school bus driver was shot and killed while driving a school bus carrying 24 students – from kindergarten through 12th grade – by a 14 year old student who had been reported to administrators by the driver for chewing tobacco on the bus. (*School Bus Driver Slain*, **NEW YORK TIMES, March 3, 2005**)

Nine Mile Falls, WA
12/10/2004
A 16 year old high school junior committed suicide with a .38-caliber handgun at his high school's entryway around 1:20 p.m. (*Student's Suicide Stuns Community; Counselors Available as School Officials Strive to 'Restore a Sense of Safety'*, **SPOKANE SPOKESMAN-REVIEW, December 12, 2004**)

Joyce, WA
3/17/2004
A 13 year old student shot and killed himself in a school classroom where about 20 other students were present. The boy reportedly brought a .22-caliber rifle hidden in a guitar case and pulled it out during the 10 a.m. class. (*Student Shoots Himself at School in Washington State*, **ASSOCIATED PRESS, March 17, 2004**)

Philadelphia, PA
2/11/2004
A 10 year old student was shot in the face and died outside a Philadelphia elementary school. A 56 year old female school crossing guard was also shot in the foot as she tried to scurry children across the street as bullets were flying and children were on the playground. (*10, Shot at School - 10-Year-Old Hit by Stray Bullet Outside School, 'This Is a Philadelphia Tragedy'*, **PHILADELPHIA INQUIRER, February 12, 2004**)

Washington, DC
2/2/2004
A 17 year old male high school student died after being shot several times and another student was injured when shots were fired near the school's cafeteria. (*Student Slain in Shooting at Ballou*, **WASHINGTON POST, February 3, 2004**)

Henderson, NV
1/21/2004
Gunman shoots and kills a hostage in his car on school campus. The gunman was allegedly looking for his ex-girlfriend as he searched the school full of children in an after-school program. (*Two Dead After Standoff*, **LAS VEGAS SUN, January 22, 2004**)

Sugar Land, TX
11/8/2003
A 17 year old female high school student was shot and killed as a fight broke out at the side of a stadium at a high school football game. (*Man Charged in Fatal Shooting Outside High School Game*, **ASSOCIATED PRESS, November 10, 2003**)

Cold Springs, MN
9/24/2003
Two students are shot and killed by a 15 year old at Rocori High School. (*Opens Fire in Central Minnesota School; One Killed, Another Critically Wounded*, **ASSOCIATED PRESS, September 25, 2003**)

Hopkinsville, KY
9/16/2003
A 16 year old girl fatally shot another teen and then killed herself as the two sat in a car parked at a shopping center across from their school. (*Police Say Teens' Deaths Near School a Murder-Suicide*, **ASSOCIATED PRESS, September 20, 2003**)

Fort Worth, TX
9/10/2003
A 16 year old boy fatally shot a classmate, then dumped his body in a nearby construction site. (*Teenager Accused of Fatally Shooting Classmate*, **FORT WORTH STAR-TELEGRAM, September 16, 2003**)

San Diego, CA
9/5/2003
A 14 year old boy jogging with his high school cross-country team is shot and killed in an ambush by his father, who then killed himself after a standoff with police. (*San Diego Teen Killed in Ambush by Father*, **ASSOCIATED PRESS, September 5, 2003**)

Red Lion, PA
4/24/2003
Principal of Red Lion Area Junior High is fatally shot in the chest by a 14 year old student, who then committed suicide, as students gather in the cafeteria for breakfast. (*Student Kills Principal at Middle School, Then Self*, **ASSOCIATED PRESS, April 23, 2003**)

New Orleans, LA
4/14/2003
One 15 year old was killed and three students wounded at John McDonough High School by gunfire from four teenagers in a gang-related shooting. (*School Officials Say They'll Bolster Security after New Orleans Shooting Leaves Teen Dead*, **ASSOCIATED PRESS, April 16, 2003**)

Tempe, AZ
1/20/2003
Reported concealed carry permit-holder Bradley William Kennedy was arrested at Arizona State University's law library with a list of judges (meant for court employees only), including their home addresses and telephone numbers. Kennedy was reportedly armed with four handguns and ammunition in violation of campus policy. Kennedy was cited for a concealed weapons permit violation, interference with the peaceful conduct of an educational institution and giving false information to police. (Jim Walsh and Carol Sowers, *List Of Judges Viewed As A Possible Threat; Found In Van Of Armed Dentist Arrested At ASU Law Library*, **THE ARIZONA REPUBLIC, January 30, 2003**)

Tucson, AZ
10/29/2002
Robert Flores, Jr. shot and killed three professors and then himself in a rampage at the University of Arizona School of Nursing, where he was a failing student. Reportedly, he reportedly told classmates about a year before that he had obtained a CCW license. (*3 Professors Shot Dead at University of Arizona*, **WASHINGTON POST, October 29, 2002**)

Bowie, MD
10/7/2002
A 13 year old by was shot and critically wounded by the DC-area sniper outside Benjamin Tasker Middle School. (*Boy Critically Wounded Outside Md. School in Latest Sniper Attack*, **ASSOCIATED PRESS, October 8, 2002**)

New York, NY
1/15/2002
Two students at Martin Luther King Junior High School in Manhattan were seriously wounded when an 18 year old opened fire in the school. (*Two Students Injured in MLK High School Shooting*, **ASSOCIATED PRESS, January 15, 2002**)

Caro, MI
11/12/2001
A 17 year old student took two hostages at the Caro Learning Center with a .22-caliber rifle and a 20-gauge shotgun, before killing himself. (*Teen*

Takes 2 Hostages at School, Kills Self, **ASSOCIATED PRESS, November 13, 2001**)

Ennis, TX
5/15/2001
A 16 year old sophomore upset over his relationship with a girl, took 17 hostages in English class, and shot and killed himself and the girl. (*Student Kills Self After Teacher Tried to Get Him to Give up Gun*, **ASSOCIATED PRESS, May 15, 2001**)

Gary, IN
3/30/2001
A 17 year old expelled from Lew Wallace High School kills classmate. (*Teen Charged in Fatal School Shooting Was Expelled Two Years Ago*, **ASSOCIATED PRESS, April 1, 2001**)

Granite Hills, CA
3/22/2001
One teacher and three students wounded by a student at Granite Hills School. (*Latest School Shooting Has Parents Seeking Alternatives*, **ASSOCIATED PRESS, March 24, 2001**)

Willamsport, PA
3/7/2001
Classmate wounded by a 14 year old girl, in the cafeteria of Bishop Neuman High School. (*One Teen Girl Shoots Another at PA Catholic School*, **PHILADELPHIA INQUIRER, March 8, 2001**)

Santee, CA
3/5/2001
A 15 year old student killed two fellow students and wounded 13 others, while firing from a bathroom at Santana High School in San Diego County. (*Two Dead, 13 Wounded when Gunman Opens Fire at High School*, **ASSOCIATED PRESS, March 6, 2001**)

Baltimore, MD
1/17/2001

A 17 year old student shot and killed in front of Lake Clifton-Eastern High School. (*Student Shot Outside Baltimore High School*, **ASSOCIATED PRESS, January 17, 2001**)

New Orleans, LA
9/26/2000
Two students were wounded in a gunfight at Woodson Middle School. (*Two Students Critically Injured in Middle School Shooting*, **ASSOCIATED PRESS, September 20, 2000**)

Lake Worth, FL
5/26/2000
A 13 year old honor student killed his English teacher on the last day of classes after the teacher refused to let him talk to two girls in his classroom. (*Police: Teacher Fatally Shot by Student on Last Day of Classes*, **ASSOCIATED PRESS, May 26, 2000**)

Prairie Grove, AR
5/11/2000
Seventh grade student injures police officer in a hay field north of the student's school after leaving campus in an apparent fit of rage. (*Student, Police Officer Hurt in Exchange of Gunfire Near School*, **ASSOCIATED PRESS, May 12, 2000**)

Savannah, GA
3/10/2000
Two students killed by a 19 year old while leaving a dance sponsored by Beach High School. (*School Dance Ends in Gunfire, Killing Two Teens, Injuring One Other*, **ASSOCIATED PRESS, March 11, 2000**)

Woodbridge, VA
3/6/2000
Deena Estaban, 42, was charged with bringing a gun onto school property. Estaban, who has a Virginia CCW license, mistakenly brought the gun in a backpack to the elementary school where she is an art teacher. The gun was loaded and had been left unattended in her classroom until another teacher discovered it. Police said that it was likely that students were near the bag during the school day. (*Teacher Unaware of Gun in Her Backpack*, **WASHINGTON POST, March 9, 2000**)

Mount Morris Township, MI
2/29/2000
A 6 year old boy shot and killed a 6 year old girl at Buell Elementary School with a .32 caliber handgun. (*Community Grieves Death of 6-Year-Old*, **ASSOCIATED PRESS, March 1, 2000**)

Fort Gibson, OK
12/6/1999
A 13 year old student, armed with a handgun, opened fire outside Fort Gibson Middle school, wounding four classmates. (*Friends, Neighbors Look for Answers in School Shooting*, **ASSOCIATED PRESS, December 7, 1999**)

Deming, NM
11/19/1999
A 12 year old boy came to school dressed in camouflage and shoots 13 year old girl with a .22 caliber as students were returning from lunch. (*Girl Shot at Middle School, Boy Taken into Custody*, **ASSOCIATED PRESS, November 19, 1999**)

Conyers, GA
5/20/1999
A 15 year old sophomore opens fire with a rifle and a handgun on Heritage High School students arriving for classes, injuring six. (*A Loud Pop, Then Chaos Again*, **ASSOCIATED PRESS, May 20, 1999**)

Littleton, CO
4/20/1999
Students Eric Harris, 18, and Dylan Klebold, 17, killed 15 students and a teacher and wounded 23 with two sawed-off shotguns and a TEC-DC9 before killing themselves at Columbine High School. (*Banned Assault Weapon Used in Littleton School Shooting*, **ASSOCIATED PRESS, April 23, 1999**)

Notis, ID
4/16/1999
Student “rode the bus to school with a shotgun wrapped in a blanket. He pointed the gun at a secretary and students, then shot twice into a door and

at the floor. He had a death list, but told one girl he wouldn't hurt anyone. He surrendered.” (B. Dedman, *Examing the psyche of an adolescent killer*, **CHICAGO SUN-TIMES, October 15, 1999**)

Springfield, OR
5/21/1998
Two teenagers were killed and more than 20 people hurt when a teenage boy opened fire at a high school, after killing his parents. The shooter, 17, was sentenced to nearly 112 years in prison. (*Second Student Dies from Wounds in Oregon School Shooting*, **ASSOCIATED PRESS, May 22, 1998**)

Fayetteville, TN
5/19/1998
Three days before his graduation, an 18 year old honor student, opened fire at a high school, killing a classmate who was dating his ex-girlfriend.(*Police Say Gunman Left Note Prior to Shooting*, **ASSOCIATED PRESS, May 28, 1998**)

Edinboro, PA
4/24/1998
A 15 year old student opened fire at an eighth-grade dance, killing a science teacher. (*3,500 Attend Pa. Teacher Funeral*, **ASSOCIATED PRESS, April 28, 1998**)

Jonesboro, AR
3/24/1998
Two boys, 11 and 13, fired on their middle school from woods, killing four girls and a teacher and wounding 11 others. (*Two Cousins in Camouflage Now Wear Orange Jail Coveralls*, **ASSOCIATED PRESS, March 25, 1998**)

West Paduach, KY
12/1/1997
Three students were killed and five wounded at Heath High School by a 14 year old. (*Shaken Students Pray for Victims of Shootings*, **ASSOCIATED PRESS, December 2, 1997**)

Pearl, MS
10/1/1997

A 16 year old student fatally shot two students and wounded seven others after stabbing his mother to death. (*Teen Passed Note Before Shooting: 'I Am Not Insane. I Am Angry'*, **Associated Press, October 2, 1997**)

Bethel, AK
2/19/1997
A 16 year old took a shotgun to school and killed the principal and a student, and injured two others. (Rhonda McBride, *Bethel remembers, reflects on school shooting anniversary*, **KTUU- TV 2 Alaska, February 19, 1997**)

Tulsa, OK
1/31/1997
Harold Glover shot and killed Cecil Herndon as 250 4-year-old children watched. Police stated that Glover and Herndon showed up at Bunche Early Childhood Development Center and argued about who would take their 4-year-old grandson home from school. Glover was in his car with his wife and grandson when he pulled out his .357 Magnum and shot Herndon, who was standing outside the vehicle, once in the chest. Glover claimed self-defense, stating that Herndon had threatened him with a pocketknife. However, authorities determined that Herndon was not acting in a "life-threatening" manner and that Glover acted without cause. (*Charges Approved for Concealed Gun Licensee; Man Faces Manslaughter Count in School Shooting*, **Tulsa World, February 12, 1997**)
